# Oral tetrahydrouridine and decitabine for non-cytotoxic epigenetic gene regulation in sickle cell disease: A randomized phase 1 study

**DOI:** 10.1371/journal.pmed.1002382

**Published:** 2017-09-07

**Authors:** Robert Molokie, Donald Lavelle, Michel Gowhari, Michael Pacini, Lani Krauz, Johara Hassan, Vinzon Ibanez, Maria A. Ruiz, Kwok Peng Ng, Philip Woost, Tomas Radivoyevitch, Daisy Pacelli, Sherry Fada, Matthew Rump, Matthew Hsieh, John F. Tisdale, James Jacobberger, Mitch Phelps, James Douglas Engel, Santhosh Saraf, Lewis L. Hsu, Victor Gordeuk, Joseph DeSimone, Yogen Saunthararajah

**Affiliations:** 1 Department of Medicine, University of Illinois Hospital and Health Sciences System, Chicago, Illinois, United States of America; 2 Jesse Brown VA Medical Center, Chicago, Illinois, United States of America; 3 Translational Hematology and Oncology Research, Taussig Cancer Institute, Cleveland Clinic, Cleveland, Ohio, United States of America; 4 Case Comprehensive Cancer Center, Case Western Reserve University, Cleveland, Ohio, United States of America; 5 Department of Quantitative Health Sciences, Cleveland Clinic, Cleveland, Ohio, United States of America; 6 Department of Hematology and Oncology, Taussig Cancer Institute, Cleveland Clinic, Cleveland, Ohio, United States of America; 7 Molecular and Clinical Hematology Section, National Institutes of Health, Bethesda, Maryland, United States of America; 8 College of Pharmacy, The Ohio State University, Columbus, Ohio, United States of America; 9 Cell and Developmental Biology, University of Michigan, Ann Arbor, Michigan, United States of America; King's College Hospital, UNITED KINGDOM

## Abstract

**Background:**

Sickle cell disease (SCD), a congenital hemolytic anemia that exacts terrible global morbidity and mortality, is driven by polymerization of mutated sickle hemoglobin (HbS) in red blood cells (RBCs). Fetal hemoglobin (HbF) interferes with this polymerization, but HbF is epigenetically silenced from infancy onward by DNA methyltransferase 1 (DNMT1).

**Methods and findings:**

To pharmacologically re-induce HbF by DNMT1 inhibition, this first-in-human clinical trial (NCT01685515) combined 2 small molecules—decitabine to deplete DNMT1 and tetrahydrouridine (THU) to inhibit cytidine deaminase (CDA), the enzyme that otherwise rapidly deaminates/inactivates decitabine, severely limiting its half-life, tissue distribution, and oral bioavailability. Oral decitabine doses, administered after oral THU 10 mg/kg, were escalated from a very low starting level (0.01, 0.02, 0.04, 0.08, or 0.16 mg/kg) to identify minimal doses active in depleting DNMT1 without cytotoxicity. Patients were SCD adults at risk of early death despite standard-of-care, randomized 3:2 to THU–decitabine versus placebo in 5 cohorts of 5 patients treated 2X/week for 8 weeks, with 4 weeks of follow-up. The primary endpoint was ≥ grade 3 non-hematologic toxicity. This endpoint was not triggered, and adverse events (AEs) were not significantly different in THU-decitabine—versus placebo-treated patients. At the decitabine 0.16 mg/kg dose, plasma concentrations peaked at approximately 50 nM (C_max_) and remained elevated for several hours. This dose decreased DNMT1 protein in peripheral blood mononuclear cells by >75% and repetitive element CpG methylation by approximately 10%, and increased HbF by 4%–9% (*P* < 0.001), doubling fetal hemoglobin-enriched red blood cells (F-cells) up to approximately 80% of total RBCs. Total hemoglobin increased by 1.2–1.9 g/dL (*P* = 0.01) as reticulocytes simultaneously decreased; that is, better quality and efficiency of HbF-enriched erythropoiesis elevated hemoglobin using fewer reticulocytes. Also indicating better RBC quality, biomarkers of hemolysis, thrombophilia, and inflammation (LDH, bilirubin, D-dimer, C-reactive protein [CRP]) improved. As expected with non-cytotoxic DNMT1-depletion, platelets increased and neutrophils concurrently decreased, but not to an extent requiring treatment holds. As an early phase study, limitations include small patient numbers at each dose level and narrow capacity to evaluate clinical benefits.

**Conclusion:**

Administration of oral THU-decitabine to patients with SCD was safe in this study and, by targeting DNMT1, upregulated HbF in RBCs. Further studies should investigate clinical benefits and potential harms not identified to date.

**Trial registration:**

ClinicalTrials.gov, NCT01685515

## Abstract 2

In a clinical trial, Yogen Saunthararajah and colleagues target DNA methyltransferase with small molecules to reactivate fetal hemoglobin which inhibits polymerisation of mutated sickle cell hemoglobin.

## Author summary

**Why was this study done?:**

- Sickle cell disease, one of the most frequent inherited diseases in humans, is driven by aggregation and precipitation of less soluble sickle cell hemoglobin in red blood cells—this destroys red blood cells and blocks blood vessels, causing organ damage, suffering, and early death.
- Fetal hemoglobin, expressed in red blood cells until infancy, mixes with sickle cell hemoglobin and promotes its dissolution. Individuals who naturally express high levels of fetal hemoglobin beyond infancy thus receive some protection from sickle complications.
- To mimic this natural state using drugs, one relevant observation was that fetal hemoglobin is increased during recovery of bone marrow from extreme stress. This led to evaluation and approval of the cytotoxic (cell killing) drug hydroxyurea, the only drug approved to treat sickle cell disease. This approach to fetal hemoglobin induction, however, is limited in potency and sustainability.
- Our goal was to increase fetal hemoglobin by inhibiting an enzyme, DNA methyltransferase 1 (DNMT1), involved in shutting off the fetal hemoglobin gene from infancy onward.

**What did the researchers do and find?:**

- We used the drug decitabine to inhibit DNMT1. However, decitabine is very rapidly inactivated in the body by another enzyme, cytidine deaminase (CDA). We therefore combined it with a CDA inhibitor, tetrahydrouridine (THU). Oral THU and decitabine were given to patients with severe, symptomatic sickle cell disease who had not derived benefit from the standard treatment of hydroxyurea.
- Oral THU-decitabine was safe and well-tolerated by the patients.
- Measurement of decitabine levels in the blood confirmed that THU enabled oral absorption of very small doses of oral decitabine. Measurements of DNMT1 protein levels and DNA methylation confirmed that this decitabine exposure was sufficient to deplete DNMT1 from cells.
- DNMT1 depletion by decitabine produced large increases in fetal hemoglobin and increased numbers of healthy red blood cells.

**What do these findings mean?:**

- Oral THU-decitabine to deplete DNMT1 safely increased fetal hemoglobin and numbers of healthy red blood cells.
- Since this was a first-in-human study, only a small number of patients were treated and for a fairly short time. Further clinical studies are therefore needed.

## Introduction

Sickle cell disease (SCD) is a congenital hemolytic anemia caused by polymerization and precipitation of mutated sickle hemoglobin (HbS, α_2_β^S^_2_) in red blood cells (RBCs). This decreases RBC life span by >90%, causing severe anemia. The unhealthy RBCs also adhere to and occlude blood vessels. Ensuing tissue hypoxia damages all organs, triggers severe pain, and compromises immunity. In low-income countries, most children with SCD do not survive to adulthood. Even in high-income countries, morbidity can be severe and life spans are reduced by several decades—the median life expectancy for people with SCD in the United States is approximately 45 years [1,2].

RBCs at the fetal stage of life contain fetal hemoglobin (HbF, α_2_γ_2_). Normal adult hemoglobin (HbA, α_2_β_2_) polymerizes with HbS, while HbF intercalates with but does not polymerize with HbS [3–5]. HbF thus interrupts SCD pathophysiology at its inception [3,4], and higher HbF correlates with fewer vaso-occlusive pain crises, less renal damage, less pulmonary hypertension, fewer strokes, and longer survival [1,6–12] (reviewed in [5]). That is, any increase in HbF provides some benefit. Those few SCD patients who inherit HbF at 20%–30% of total hemoglobin (hereditary persistence of fetal hemoglobin, HPFH) have essentially normal life spans [13–15]. Decades of research has hence been directed towards pharmacologic recapitulation of this naturally selected protective state [5]. A key observation directing early efforts was that HbF is enriched during bone marrow recovery from extreme stress [16–21]. Cytotoxic (cell killing) drugs can create such stress, leading to evaluation in SCD of the oral ribonucleotide reductase inhibitor hydroxyurea [20–22]. In a pivotal trial, hydroxyurea (15–35 mg/kg) increased HbF for 2 years in approximately 50% of the adult SCD patients treated [22,23]. HbF induction correlated strongly with increased RBC half-life [24,25], fewer pain crises [23], and better quality of life [26]. Trial patients with HbF levels >0.5 g/dL survived longer [8]. Average HbF increases at 2 years, however, were modest (3.6%) [20–23,27]. Moreover, HbF increases were particularly unlikely in patients with the lowest baseline HbF levels and thus at highest risk of morbidity and mortality [23,25,28,29], and even patients with excellent initial HbF inductions demonstrated diminishing inductions over time [23,30]. Lower and less durable HbF increases also correlated with fewer reticulocytes (<300,000 × 10^9^/L) and neutrophils (<7.5 × 10^9^/L) at baseline. This correlation underscores that HbF induction by cytotoxicity requires reserves of hematopoietic precursors sufficient to repeatedly mount recoveries from bone marrow stress that destroys their counterparts [21,23]. Cumulative attrition of these reserves occurs via vaso-occlusion in the marrow and to the kidneys [23,25,28,29]. This is a problem even separate from considerations of sustainable HbF induction via cytotoxicity: in SCD, erythropoiesis has to operate at >10-fold the normal rate to barely sustain hemoglobin levels compatible with life, and dwindling compensatory capacity is a major cause of early death [8,23,31,32]. Therefore, new, non-cytotoxic, durable, and more potent methods of inducing HbF are needed.

The chromatin-modifying enzyme DNA methyltransferase 1 (DNMT1) maintains methylation marks on DNA through cell division. DNMT1 is also a corepressor—it executes the biochemical work of silencing genes for sequence-specific DNA-binding factors, e.g., BCL11A and TR2/TR4, that direct epigenetic silencing of the HbF gene (*γ-globin*, *HBG*) [33–45]. The deoxycytidine analog decitabine can deplete DNMT1—a nitrogen substituted for a carbon in the decitabine pyrimidine ring covalently binds to DNMT1 and causes its degradation [46]. Importantly, the decitabine deoxyribose moiety is unmodified; thus, it can incorporate into the elongating DNA strand during S-phase without terminating chain extension or causing cytotoxicity [47,48]. High concentrations of decitabine, however, do produce off-target anti-metabolite effects and cytotoxicity, in significant part via the uridine moiety degradation products that can misincorporate into DNA or inhibit thymidylate synthase [49,50]. Decitabine regimens approved by the US Food and Drug Administration (FDA) for the treatment of myeloid cancer, having evolved out of the traditional cytotoxic intent of oncology, utilize such cytotoxic doses and require pulse-cycled administration to recover from cytotoxic side effects. We therefore redesigned decitabine application for non-cytotoxic, molecularly-targeted therapy of non-malignant and malignant diseases [43,51–54]. Specifically, we selected demonstrably non-cytotoxic yet DNMT1-depleting doses, used a subcutaneous route of administration to blunt C_max_, and administered these doses frequently and in distributed fashion (2–3X/week) to increase the fraction of target cells exposed in S-phase, as DNMT1-depletion by decitabine is S-phase–dependent [43,51–54]. This approach increased HbF by >10% in SCD patients who had no HbF response to hydroxyurea in the pivotal clinical trial (i.e., a mean change in HbF after 2 years of treatment with hydroxyurea of 0.3%) [42,43,55]. Hindering wide clinical application of decitabine in this way, however, are a number of limitations with its pharmacology: it has a very brief plasma half-life of only minutes [56,57] (a problem since DNMT1-depletion is S-phase, and hence exposure-time, dependent); has negligible solid tissue distribution (a problem when target cells reside in solid tissues, e.g., erythroid precursors in the spleen in β-thalassemia); and hence also has negligible oral bioavailability (a problem for worldwide and long-term therapy). These pharmacology problems have a common cause: rapid deamination/inactivation of decitabine to uridine degradation products by the pyrimidine metabolism enzyme cytidine deaminase (CDA) that is highly expressed in tissues such as the intestines and liver [58–62].

Thus, we combined a CDA inhibitor, oral tetrahydrouridine (THU), with oral decitabine: THU is a uridine analog competitive inhibitor of CDA that, although not FDA-approved for any indication (new chemical entity), has not caused toxicity to animals or to humans in several clinical trials, including a trial with oral administration exceeding a year [40,59–61,63–69]. Here, building on encouraging results from pre-clinical in vivo studies [70], we report the first-in-human clinical translation of oral THU–decitabine for HbF induction in SCD. Specific goals were to evaluate safety of oral THU–decitabine and recommend a phase 2 dose for DNMT1-depletion and HbF induction in SCD [70].

## Materials and methods

### Ethics statement

All research involving human participants was approved by the Cleveland Clinic and University of Illinois at Chicago Institutional Review Boards (IRBs), and all clinical investigation was conducted according to the principles expressed in the Declaration of Helsinki. Written informed consent was obtained from the participants on the IRB approved protocol. The protocol number was Case 10z11. The United States FDA Investigational New Drug number is 112,914.

### Study design

This was a single-blind dose-escalating phase 1 clinical trial with a maximum of 5 decitabine dose levels. Five patients were enrolled at each dose level, randomized with 3:2 odds to study drugs versus placebo (5 cohorts of 5 randomized patients).

This design was not altered during the course of the study.

### Patient population

Written informed consent was obtained prior to treatment in all patients. The treatment population was adult (≥18 years of age) SCD (SS or S-β-thalassemia) patients who despite standard-of-care hydroxyurea for ≥6 months (or being intolerant or unwilling to take hydroxyurea) were at risk of early death as defined by published criteria [8]. These criteria were at least 1 of the following: (i) HbF <0.5 g/dL, (ii) 3 or more pain episodes per year requiring parenteral narcotics, (iii) 1 or more acute chest syndrome episodes, and (iv) total hemoglobin <9 g/dL and absolute reticulocyte count ≤250,000 × 10^9^/L.

### Interventions

Decitabine and THU were synthesized by Ash Stevens (Detroit, MI). Drugs were stored in glass bottles at −20°C. Bottles were opened after equilibration to room temperature. The appropriate amount of drug was weighed out and reconstituted with water for consumption by patients in the clinic within 30 minutes of drug reconstitution. Placebo was an equivalent amount of water without study drug.

**Fig 1:**
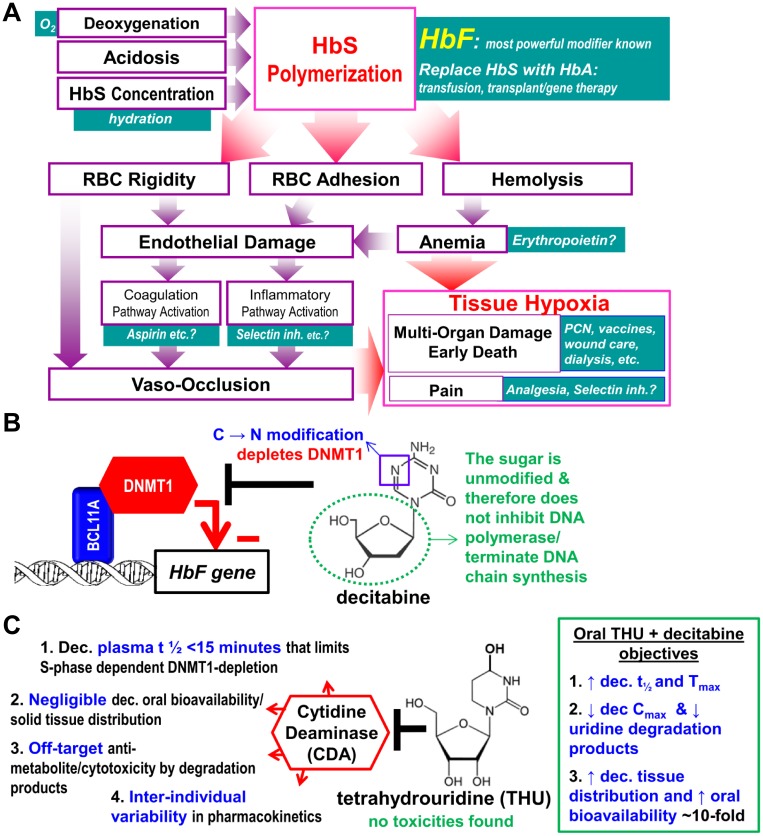
Fetal hemoglobin (HbF) blocks polymerization of deoxy sickle hemoglobin (HbS), the root cause of sickle cell disease (SCD) pathophysiology, and is the most powerful known disease modifier. **(A)** Polymerization of deoxy HbS drives all SCD pathophysiology; In contrast to HbF, normal adult hemoglobin (HbA, ẞ-chains) can participate in polymerization. **(B)** The gene for HbF (*HBG*) is silenced by DNA methyltransferase 1 (DNMT1). Although DNA-binding factors, e.g., BCL11A, direct this silencing, the biochemical work of epigenetic repression is executed by chromatin-modifying enzymes, amongst which DNMT1 is central. Decitabine depletes DNMT1 and can do so without cytotoxicity because in contrast to other cytidine analogues (e.g., cytarabine) the deoxyribose moiety (green dotted circle) is natural, although higher concentrations do cause anti-metabolite effects and DNA damage, in part by degradation into uridine counterparts that misincorporate into DNA. **(C)** Several pharmacologic limitations of decitabine hinder safe, effective, practical clinical translation. The limitations have a common cause, the enzyme cytidine deaminase (CDA). Tetrahydrouridine (THU) inhibits CDA. No toxicities have been found for THU in animals or humans.

**Fig 2:**
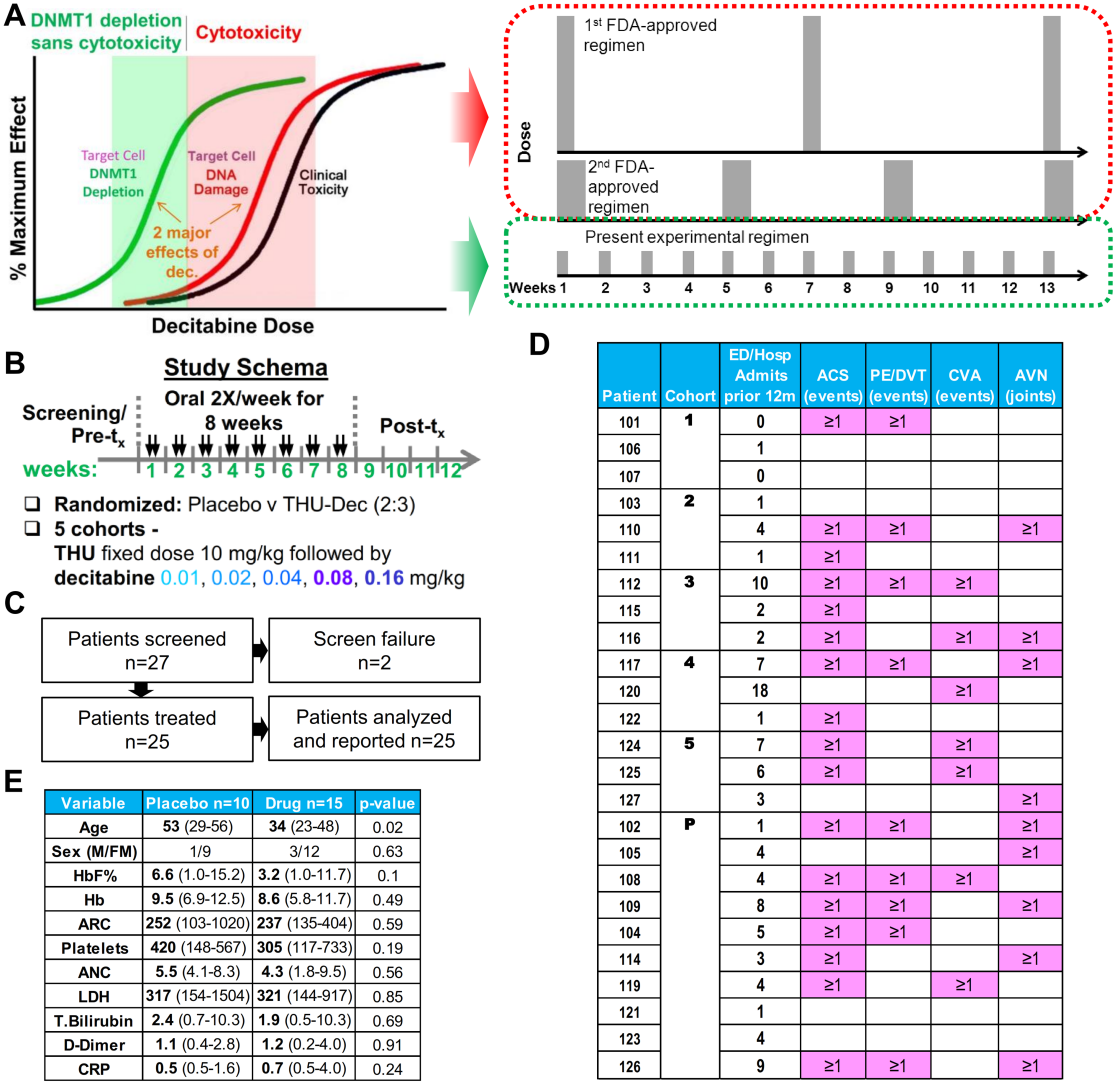
Design rationale, schema, flow, and patient characteristics. (A) Decitabine regimens approved by the US Food and Drug Administration (FDA) to treat myeloid malignancy utilize cytotoxic decitabine doses (red zone), requiring pulse-cycled administration to recover from cytotoxic side effects. This clinical trial instead escalated oral decitabine doses from almost zero (0.01 mg/kg) gradually upward to find the minimum doses required to deplete DNA methyltransferase 1 (DNMT1) without cytotoxicity (green zone) when administered after oral tetrahydrouridine (THU) 10 mg/kg. Because such doses are non-cytotoxic, they can be administered frequently in distributed fashion to increase the fraction of target cells subject to S-phase–dependent DNMT1 depletion. (B) The study schema. (C) Flow of patients through the trial. (D) Patient clinical characteristics at baseline. Abbreviations: ACS, acute chest syndrome; AVN, acute vascular necrosis; CVA, cerebral vascular accident; ED/Hosp, emergency department/hospital; PE/DVT, pulmonary embolus/deep vein thrombosis. (E) Patient demographics and baseline laboratory variables. Median and range showed for continuous variables. *P* values Wilcoxon ranked sums 2-tailed or Fisher exact test.

Fixed-dose oral THU 10 mg/kg was administered 60 minutes before oral decitabine at 0.01, 0.02, 0.04, 0.08, or 0.16 mg/kg (Figs 1, 2A and 2B). Repeat dose administration 2X/week for 8 weeks, instead of single dose administration, was used to assess safety and efficacy—to increase the likelihood that the dose identified for further studies would be safe and efficacious for the intended application of chronic disease modification (Fig 2A and 2B).

Patients at each dose level were monitored weekly to determine if next treatments should be withheld based on laboratory endpoints; in previous clinical trials, the most sensitive indices of decitabine biologic activity were an increase in the platelet count and a decrease in the absolute neutrophil count (ANC). The plan was to use threshold values of these parameters not associated with clinical toxic events, being within the range observed in patients with SCD during their routine care with hydroxyurea and/or splenectomy, to trigger dose modification, and thereby maintain safety [43,51]. These thresholds were ANC <1.5 × 10^9^/L and platelets >1,200 × 10^9^/L. Non-hematologic toxicity ≥grade 3 attributed to study drug was also to trigger a dose modification, as was decitabine C_max_ > 0.2 μM. Drug was to be held until recovery below these thresholds, when drug was to be restarted with a 25% decrease in dose.

Concurrent hydroxyurea therapy was explicitly disallowed, with a requirement for a 28-day washout period from the last hydroxyurea dose to initiation of study drug or placebo.

### Outcomes

The primary endpoint was ≥ grade 3 non-hematologic toxicity. In addition, the a priori study design required dose modification for platelets >1,200 × 10^9^/L, or neutrophils <1.5 × 10^9^/L. Our goal was to provide evidence in favor of a null hypothesis that patients in treated groups (oral THU-decitabine 2X/week over 8 weeks; *n* = 15) do not experience treatment-related events requiring dose modification more than patients in the placebo group (*n* = 10). Secondary endpoints included (i) sickle cell crisis frequency (efficacy), (ii) coagulation (D-dimer) and inflammatory (C-reactive protein [CRP]) pathway activity (efficacy), (iii) HbF levels measured by high-performance liquid chromatography (HPLC) (efficacy and pharmacodynamics of decitabine), (iv) DNA methylation levels at repetitive elements in buffy coat DNA (pharmacodynamics of decitabine), and (v) DNMT1 levels in buffy coat cells (pharmacodynamics of decitabine). A priori secondary endpoints not measured for cost and technical reasons were *CDA* genotype and CDA functional activity in serum.

### Sample size

The study employed a randomized design to identify an oral dose of decitabine that can be administered twice a week in combination with oral THU over an 8-week period without requiring dose modification. We used a standard 3+3 dose escalation design with at most 5 dose levels. There would thus have been 6 in the maximum dose group if a dose-limiting toxicity had been detected. Such a 6-patient group would have detected at least 1 toxicity with a probability of 0.88 if it occurred in 30% of the population; this value is calculated as 1 minus the probability of zero toxicities, i.e., 1 − 0.7^6^. Ten patients were treated with placebos, 2 at each dose level, so the maximum sample size was 15 + 10 = 25. If a dose modification was required because of ANC < 1.5 × 10^9^/L or platelets > 1200 × 10^9^/L or ≥ grade 3 non-hematologic toxicity attributed to the study drug, in 1 or more of the 3 patients who received the study drug at a dose-level cohort, the plan was to accrue another cohort of 5 new patients (3:2 study-drug:placebo) with the treatment dose based on the cumulative dose administered to patients receiving the study drug in the preceding cohort. The Safety and Intent-To-Treat (ITT) populations included all enrolled patients receiving at least one dose of decitabine.

### Randomization procedures

Randomization was at the University of Illinois at Chicago by Dr. Michael Pacini using a randomization table created on www.randomization.com. Patients were randomly allocated to study drug treatment versus placebo.

### Blinding

Patients, but not investigators, were blinded as to the assigned treatment. The experimental treatment was highly diluted in water. Therefore, the method of patient blinding was administration of water placebo with similar appearance, volume, and taste as the study drug.

### Pharmacokinetics

Blood was collected for pharmacokinetic analysis at 0, 2, 4, and 24 hours after the first decitabine dose (week 1). Blood samples were drawn over a period of less than 1 minute into tubes pre-loaded with heparin and THU 10 μg/ml (to prevent in vitro metabolism by CDA) and immediately transferred onto ice. Samples were then centrifuged as soon as possible at 600 g for 5 minutes at 4°C. After separation, plasma was transferred in 0.2 ml aliquots into pre-frozen vials and stored frozen at −80°C until shipment to Ohio State University for analysis (shipment on dry ice) by a liquid chromatography tandem mass spectrometry (LCMS/MS) method that has been previously described in detail for determination of decitabine in human, baboon, mouse, and rat plasma [56,57,70]. Pharmacokinetic data were analyzed using non-compartmental methods or a 2-compartment model with instantaneous/intravenous input using the R package PKLMfit. The model-fitting method allowed estimation of terminal half-lives for some data sets. The AUC_last_ (the area under the curve from the time of dosing to the last measurable concentration) was calculated by the linear trapezoidal method.

### DNMT1 protein measurement by flow cytometry of peripheral blood mononuclear cells (pharmacodynamic analyses)

Phlebotomized whole blood was layered over Histopaque. The interface was collected and washed with phosphate-buffered saline (PBS). Cell suspensions of approximately 200,000 cells were fixed with 4% paraformaldehyde for 30 minutes on ice. Cells were spun down, washed in PBS, and suspended in 70% ethanol and stored at −20°C for subsequent batched analyses. For analyses, patient samples were thawed and centrifuged. All procedures were performed on ice and all centrifugations were done at 400 g for 5 minutes at 4°C. The pellet was hydrated overnight in 1 ml sterile, distilled water to partially reverse the alcohol fixation. After incubation, samples were centrifuged and then resuspended in 1 ml PBS/2% bovine serum albumin (BSA) and incubated for 30 minutes to block nonspecific antibody binding. Following centrifugation, unlabeled anti-DNMT1 antibody [EPR 3522] (0.0625 μg/test; Abcam; catalog no. ab92314) was added in a final volume of 100 μl and incubated for 1 hour. Samples were washed 3 times: each wash was a 10-minute incubation with 1 ml PBS/2% BSA followed by centrifugation. After the third wash, CD64-Alexa Fluor 488 (5 μl/test; BioLegend; catalog no. 305010), Cyclin A2-PE (4μl/test; Beckman Coulter; catalog no. B15092), CD33-APC/Cy7 (5 μl/test; BioLegend; catalog no. 366614), and F(ab′)2-goat anti-rabbit IgG (H+L) Alexa Fluor 647 (0.0938 μg/test; Life Technologies; catalog no. A21246) were added in a final volume of 100 μl and incubated for 1 hour. After the incubation and without washing, 3.5 ml PBS containing 0.5 μg/ml DAPI was added to each sample. All samples were analyzed on an Attune NxT Acoustic Focusing Cytometer (Life Technologies) at a flow rate of 500 μl per minute. Compensation was performed with CompBeads Set Anti-Mouse Ig, κ (BD Biosciences; catalog no. 552843) and Flow Cytometry Protein G Antibody Binding Beads (Bangs Laboratories, Inc.; catalog no. 554/11863).

#### Data analysis

Data were normalized to the instrument using 8-peak SPHERO Rainbow Calibration Particles (Spherotech; catalog no. RCP-30-5A). WinList 3D v8.0 (Verity Software House) was used for post-acquisition analysis. Doublet discrimination was performed using the DAPI area and peak signals. Gated singlet events were displayed in a bivariate plot of cyclin A2 versus DNA content to identify S + G2 + M phase cells as both cyclin A2 positive and >2C DNA. These color-evented cells were used as a guide to set the boundary between DNMT1 positive and negative events. The median DNMT1-negative value was subtracted from the median DNMT1-positive value. This net value was further processed by normalization using linear regression of 8 peak bead sets between runs performed on different days.

### Methylation level of LINE-1 repetitive element CpG by pyrosequencing

DNA was purified from peripheral blood mononuclear cells (isolated by Ficoll-Hypaque density centrifugation) using the Wizard Genomic DNA Purification Kit (Promega). DNA was bisulfite-converted using EZ DNA Lightning Methylation kit (Zymo Research). The PCR primers were 10 pmol of 5′- TTTTTTGAGTTAGGTGTGGG-3′ and 10 pmol of biotinylated-5′-TCTCACTAAAAAATACCAAAC-3′. PCR cycling conditions were: cycle temperature of 94°C for 30 seconds, 60°C for 30 seconds, and 72°C for 30 seconds, for 45 cycles to consume all the biotinylated primers. The biotinylated strand was captured on streptavidin sepharose beads (Amersham Biosciences, Uppsala, Sweden) and annealed with the sequencing primer 5′-GGGTGGGAGTGAT-3′. The methylation degree of long interspersed nuclear elements (LINE-1) was computed at 3 CpG sites pyrosequenced with the Pyromark Q24 Pyrosequencer (Qiagen) using the dispensation order GTCGATTAGTAGTCAGTCGTATTGTATC.

### Clinical pathology tests

Blood counts, blood chemistries, total bilirubin, HbF, LDH, and D-dimer levels were standard clinical pathology tests through the CLIA-certified Clinical Pathology Laboratory at the University of Illinois at Chicago.

### Measurement of HbF levels

Analysis of globin chains was performed on a TSP Spectra HPLC system using a LiChristopher 100 RP-8 column and a gradient of acetonitrile-methanol-NaCl as per the standard CLIA-certified Clinical Pathology Laboratory methods at University of Illinois at Chicago. For quantification of F-cells by flow cytometry, peripheral blood samples were fixed and stained with phycoerythrin-conjugated anti-HbF (Caltag) per manufacturer’s instructions. A normal adult negative control and cord blood positive control were run with each Becton-Dickinson FacsCalibur (Sunnyvale, CA) analysis.

### Statistics

For statistical comparisons of adverse events (AEs) between placebo and each dose group, we performed exact tests of the null hypothesis that the rate parameters of Poisson distributed AEs are equal. Relative risks are given as the ratio of such rate parameters. Thus, differences are insignificant if relative risks confidence intervals include 1. The R function poisson.test(), an exact test, was used. Pain, the most frequent AE, was analyzed similarly.

For time course analyses of HbF and total hemoglobin endpoints in placebo versus each dose group, linear functions of time were fitted to each patient’s time course of measurements on-treatment. The slopes of such fits versus the dose that the patient received were fitted to linear dose-response to generate *P* values of such slopes of slopes, i.e., lines in dose-response plots. This approach controls for inter-patient differences in initial values as per the planned mixed-effects modeling.

## Results

### Patient flow and characteristics

The trial protocol is provided as S1 Text, the CONSORT statement as S2 Text, and the patient flow is shown in Fig 2C. Twenty-seven patients were screened and 25 eligible patients enrolled at the University of Illinois at Chicago with the first patient starting the study drug on Sept 5, 2012 and the last patient receiving the last dose of the study drug on Jan 6, 2016. Patients met inclusion criteria for high-risk disease despite standard of care, and hydroxyurea treatment was discontinued at least 28 days prior to initiation of the study drug or placebo [8] (Fig 2D). Placebo-treated patients were older than study drug–treated patients, a difference produced randomly. Median age of the 4 male and 21 female patients was 34 years (range 23–56) (Fig 2E). Baseline laboratory values reflected hemolytic anemia and were similar in drug- and placebo-treated patients (Fig 2E).

### AEs

**Fig 3:**
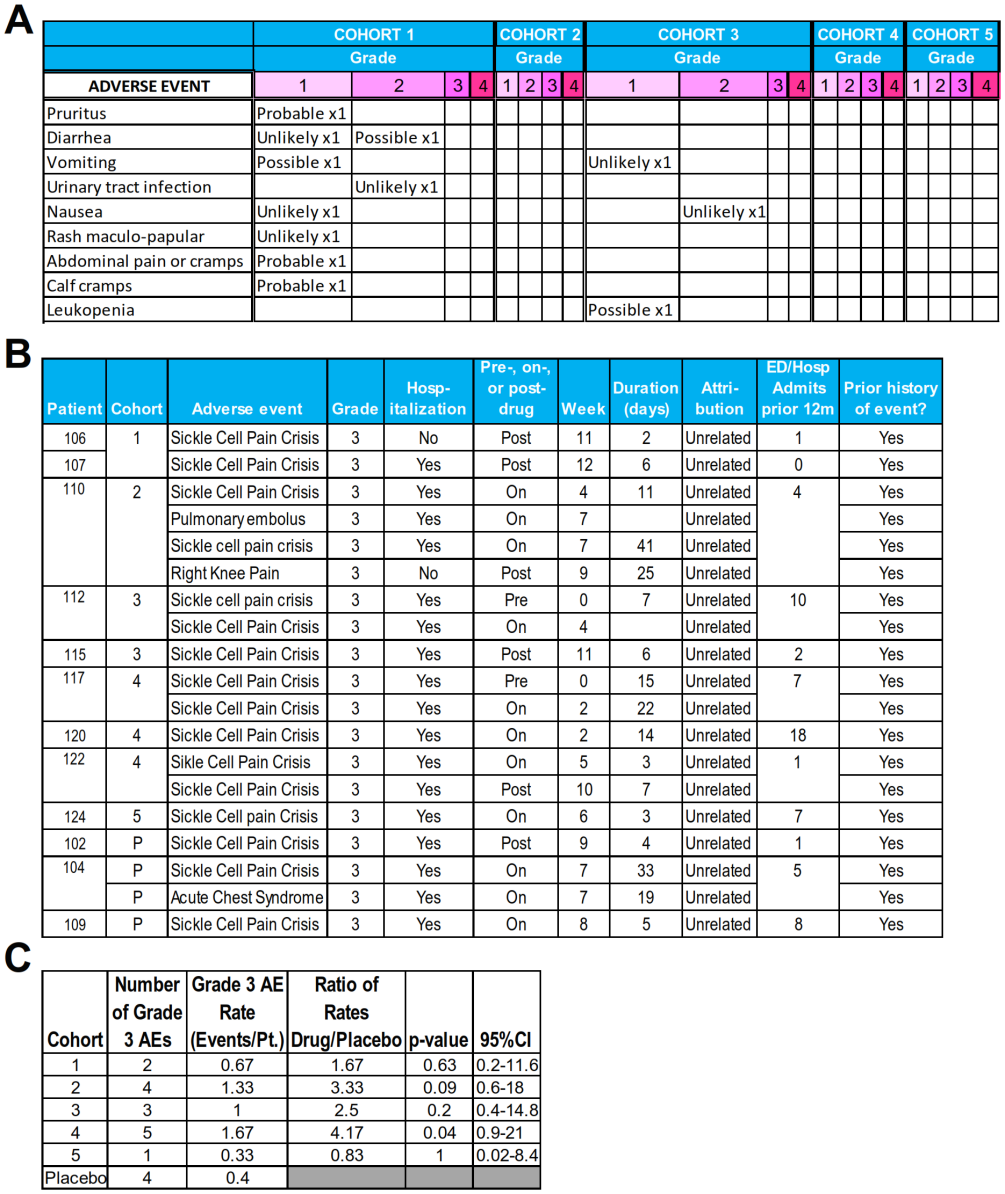
Adverse events (AEs). **(A)** AEs with possible relatedness to the study drug (AEs other than “unrelated”) per judgement of the treating clinical teams. **(B)** All grade 3 AEs occurring in study drug—and placebo-treated patients. These grade 3 AEs were sickle cell complications judged to be unrelated to the study drug by the treating clinical teams. **(C)** Statistical comparison of grade 3 AEs between placebo and decitabine dose-level patients. R function poisson.test(), an exact test, was used. Abbreviation: ED/Hosp, emergency department/hospital.

The primary endpoint of ≥ grade 3 non-hematologic toxicities was not triggered, nor did any patients trigger the platelets >1,200 × 10^9^/L, or neutrophils <1.5 × 10^9^/L thresholds requiring dose modification. There were no grade 4 AEs (Fig 3A).

This study was not designed or powered to demonstrate clinical benefit (e.g., reduction in grade 3 AEs from SCD). Such grade 3 AEs unrelated to the study drug occurring in study drug–treated patients were sickle cell vaso-occlusive pain crises (X14) and a pulmonary embolus (Fig 3B). Of these 15 events in study drug–treated patients, 2 events occurred prior to study drug administration (between study enrollment and initiation of drug), and 6 events occurred after discontinuation of study drug, including 3 events in week 3 or 4 of post-drug follow-up (Fig 3B). The pulmonary embolus occurred in a patient with prior history of this complication and without baseline or on-treatment platelet count elevations (a cohort 2 patient). All patients except for one had required emergency room or hospital admission for sickle cell vaso-occlusive pain crises between 1 and 18 times in the 12 months prior to study enrollment (Fig 3B). By including the event occurring prior to study drug administration, the rate of grade 3 sickle cell vaso-occlusive pain crises was statistically significantly increased in cohort 4 versus placebo patients: cohort 4 patients had a rate of this complication in the 12 months preceding study enrollment that was more than 2-fold greater than the rate in placebo-treated patients (Fig 3B).

**Fig 4:**
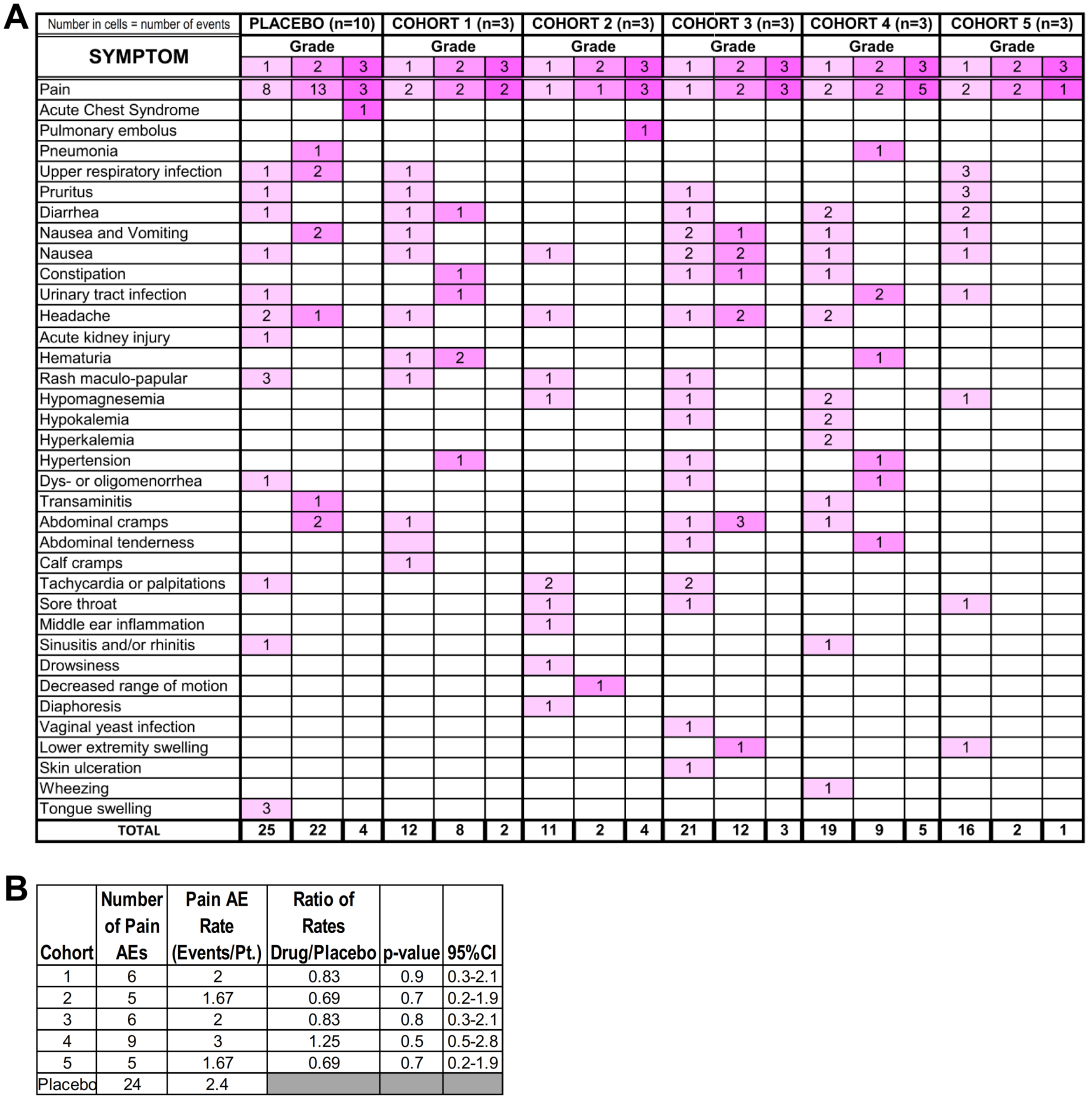
Adverse events (AEs) in placebo and decitabine dose-level patients (cohorts 1–5). **(A)** All AEs as per Common Terminology Criteria for Adverse Events (CTCAE) v 4.0. There were no grade 4 AEs. **(B)** Statistical comparison of all pain AEs in drug-treated versus placebo cohorts. R function poisson.test(), an exact test, was used.

The most frequent AE of any grade in placebo- and study drug–treated patients was pain from vaso-occlusive sickle cell crisis (Fig 3A). The rates of vaso-occlusive pain crisis AEs of all grades appeared lower in THU-decitabine dose level versus placebo-treated patients except in cohort 4, as noted above (Fig 4). The overall pattern of AEs was similar in placebo- and study drug–treated patients (Fig 4).

No patients discontinued the study drug or placebo because of AEs.

### Decitabine pharmacokinetics

**Fig 5:**
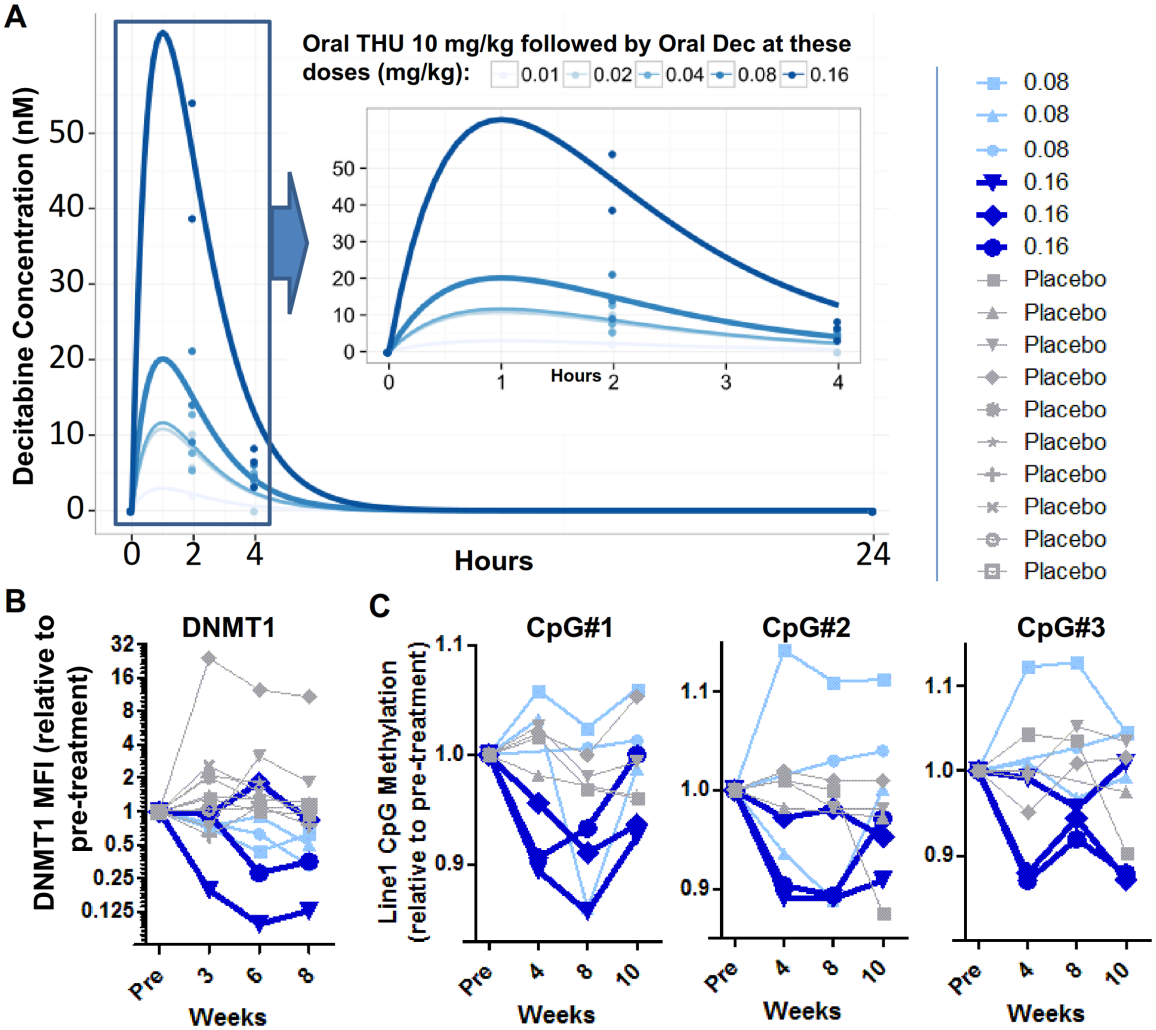
Pharmacokinetics (PK) and pharmacodynamics (PD) of oral tetrahydrouridine (THU)-decitabine. **(A)** Decitabine PK. Samples for PK analysis were obtained in 12 of the 15 patients who received the study drug. Times are listed in hours after administration of oral decitabine. Data points are measured values and curves are fits using the R package PKLMfit. The inset shows a close-up of hours 0–4. Decitabine was quantified by a validated liquid chromatography tandem mass spectrometry (LCMS/MS) method. **(B)** DNMT1 protein levels in peripheral blood mononuclear cells (PBMC) measured by flow cytometry in cohorts 4 and 5 (decitabine 0.08 mg/kb and 0.16 mg/kg, respectively) and in all placebo-treated patients. Analyses were blinded to treatment assignment. Shown are means of 2 independent measurements. **(C)** Methylation of long interspersed nuclear elements (LINE-1) repetitive element CpGs (3 individual CpGs) in PBMC in cohorts 4 and 5 placebo and drug-treated patients. Analyses were blinded to treatment assignment. Measurements were made by pyrosequencing. Shown are means of 2 independent measurements.

Samples for decitabine pharmacokinetic measurements by LCMS/MS were obtained immediately prior to and at 2, 4, and 24 hours after decitabine administration in 12 of 15 study drug–treated patients (dictated by venous access). Decitabine was detected in the plasma even at the lowest decitabine dose level of 0.01 mg/kg, and a dose-dependent increase was observed (Fig 5A). The highest dose of oral decitabine, 0.16 mg/kg (cohort 5), produced decitabine plasma concentrations at 2 hours from 39–54 nM (C_max_). The dose level below this, 0.08 mg/kg (cohort 4), produced C_max_ of 9–21 nM (Fig 5A).

### Molecular pharmacodynamics

The intended molecular pharmacodynamic effect with oral THU-decitabine therapy is DNMT1 depletion. DNMT1 protein levels were measured by flow cytometric analysis of peripheral blood mononuclear cells obtained at baseline, 3, 6, and 8 weeks after initiation of treatment. DNMT1 protein levels decreased by >75% from baseline in 2 of the 3 decitabine 0.16 mg/kg–treated patients (cohort 5), and by approximately 50% in all 0.08 mg/kg–treated patients (cohort 4), but not in patients with lower decitabine doses or in placebo-treated patients (Fig 5B). An expected consequence of DNMT1 depletion is reduction in DNA methylation at LINE-1 repetitive element CpGs, a measurement that has been used in other clinical trials of DNMT1-depleting drugs [71]. LINE-1 CpG methylation was measured in peripheral blood mononuclear cells obtained at baseline, 4, 8, and 10 weeks after initiation of therapy. LINE-1 CpG methylation decreased consistently by approximately 10% with decitabine at 0.16 mg/kg (cohort 5), but not with lower decitabine doses or in placebo-treated patients (Fig 5C).

### Induction of HbF

**Fig 6:**
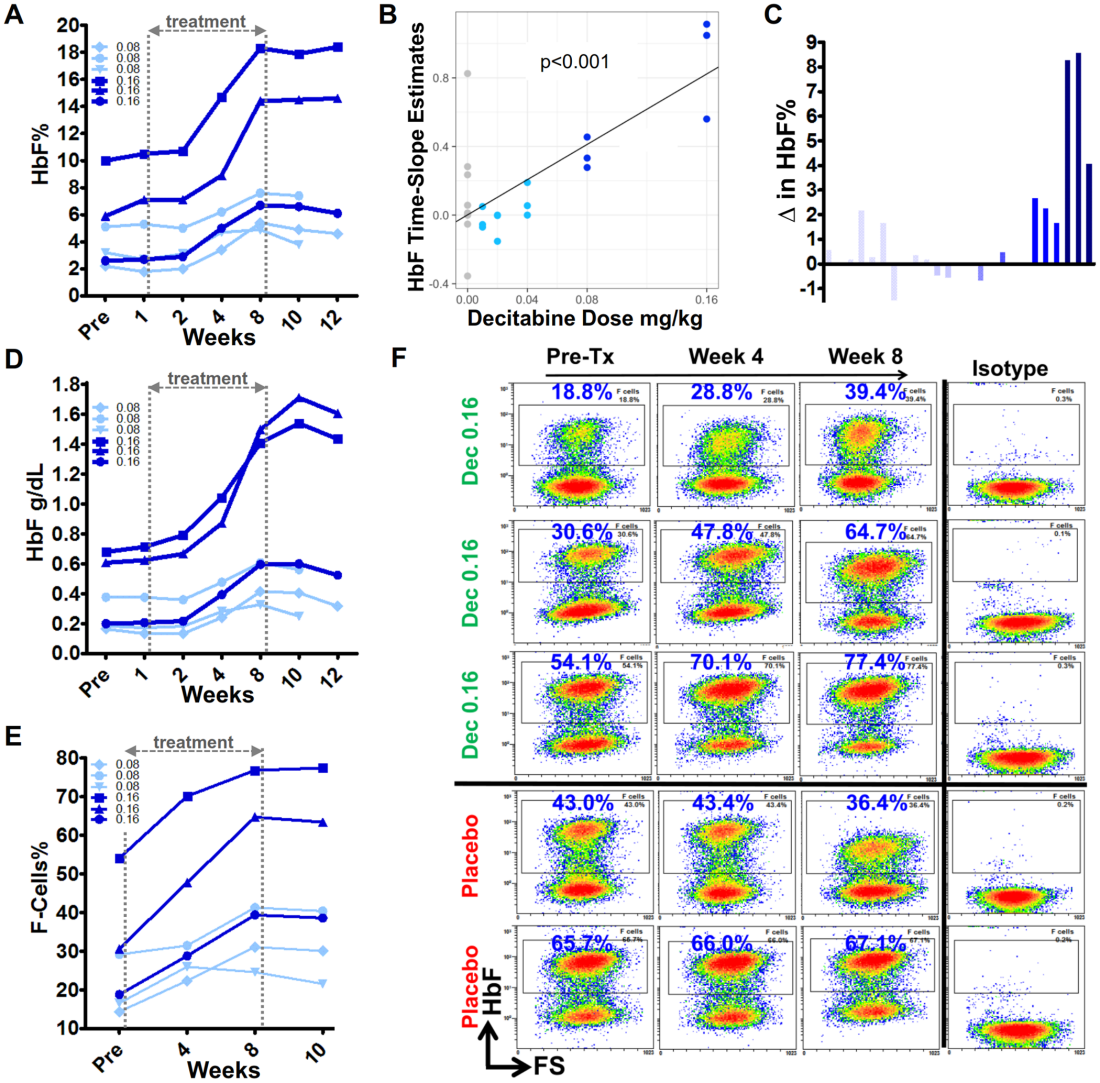
Fetal hemoglobin (HbF) induction in cohort 4 and 5 patients. **(A)** HbF percentage (HbF%) over time in cohorts 4 and 5 (decitabine 0.08 mg/kg and 0.16 mg/kg, respectively) patients. **(B)** Statistical analysis of the rates of change in HbF% with increasing doses of decitabine (0 = placebo); *P* value determined by linear regression. **(C)** Change in HbF% from pretreatment to week 8 or 10. **(D)** Absolute HbF levels. **(E)** Proportion of red blood cells (RBCs) expressing high levels of HbF (F-cells) in cohort 4 and 5 patients. **(F)** Raw F-cell flow cytometry data for cohort 5 tetrahydrouridine (THU)-decitabine—and placebo-treated patients. Abbreviation: FS, forward scatter.

The primary efficacy objective with non-cytotoxic DNMT1 depletion in patients with SCD is to increase HbF expression in erythroid precursors, and thereby to decrease HbS polymerization and stop the SCD pathophysiological cascade at its inception (Fig 1). HbF was measured by HPLC in the Clinical Pathology Laboratory and also by flow cytometric quantification of HbF content in individual RBCs. HbF percentage (HbF%) increases by HPLC were observed with decitabine 0.08 mg/kg and 0.16 mg/kg (cohorts 4 and 5, respectively), but not with other decitabine dose levels or placebo (Fig 6A–6D, S1 Fig, S1 Data). Rates of increase in HbF% (time-slope estimates) increased with dose (*P* < 0.001) (Fig 6B). The largest HbF increases were in cohort 5 with increases of 4%, 9%, and 9%, corresponding to absolute HbF increases of 0.4, 0.85, and 1.1 g/dL from baseline (Fig 6A–6D). Upon study drug discontinuation, HbF plateaued for 2 weeks and then began to decline (Fig 6A and 6C). By flow cytometry, major increases in RBC enriched for HbF (F-cells) were observed in cohorts 4 and 5, with the largest F-cell increases in cohort 5 patients, up 2.1-, 2.1- and 1.4-fold, thereby reaching levels up to approximately 40%, 65%, and 80% of total RBCs, respectively (Fig 6E and 6F, S2 Fig). F-cells did not increase with placebo (Fig 6E and 6F). F-cells plateaued in the 2 weeks after study drug discontinuation (Fig 6D).

### Total hemoglobin and other hematology/efficacy parameters

**Fig 7:**
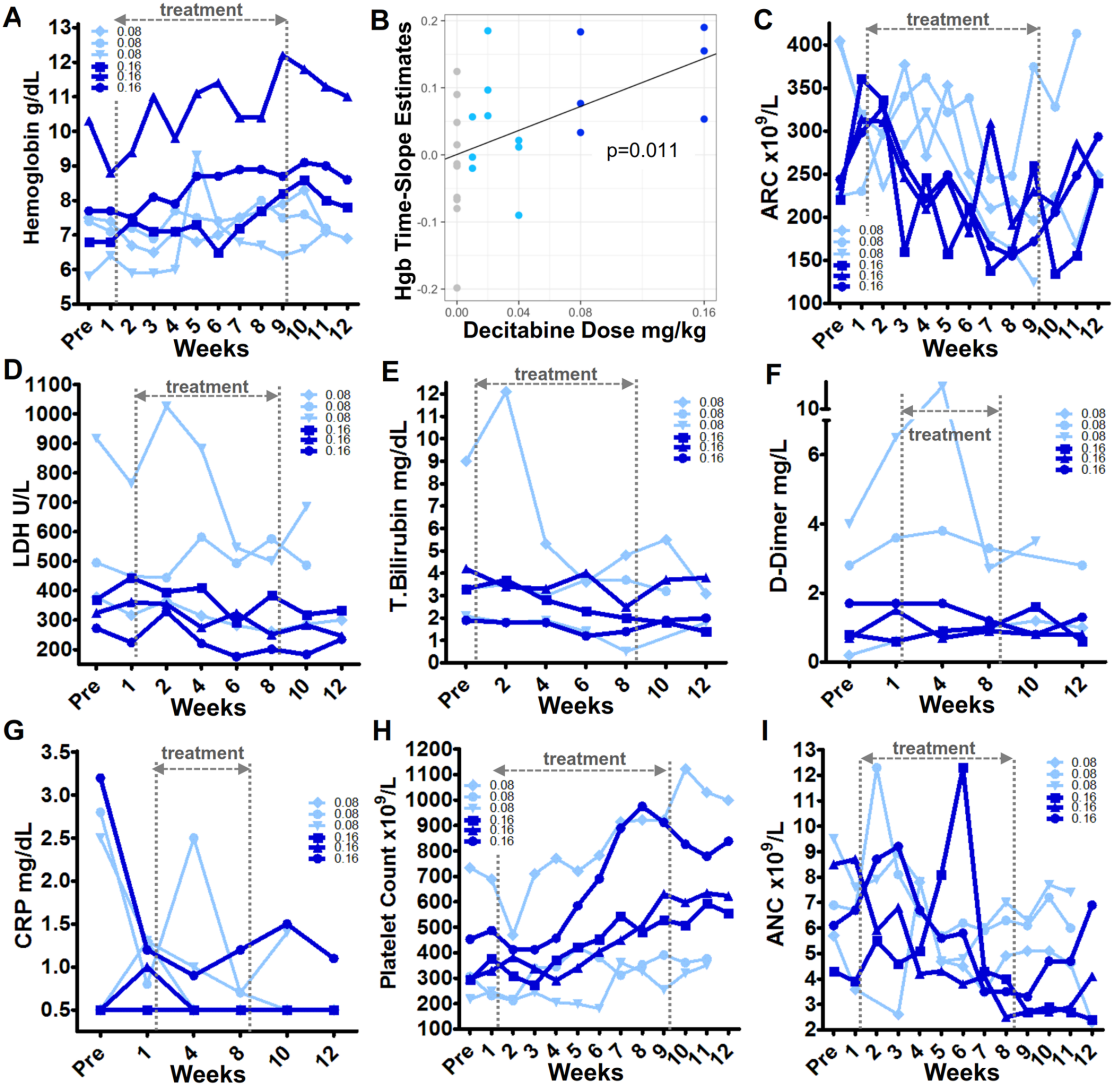
Blood counts and other parameters in cohort 4 (decitabine 0.08 mg/kg) and 5 (decitabine 0.16 mg/kg) tetrahydrouridine (THU)-decitabine–treated patients. **(A)** Total hemoglobin. **(B)** Statistical analysis of the rates of change in total hemoglobin (Hbg) with increasing doses of decitabine (0 = placebo); *P* value determined by linear regression. **(C)** Absolute reticulocyte counts (ARC). **(D, E)** LDH and total bilirubin. These are biomarkers of hemolysis. **(F)** D-dimer. A biomarker of coagulation activation. **(G)** C-reactive protein (CRP). A biomarker of inflammation. **(H)** Platelets. **(I)** Absolute neutrophil counts (ANC).

Non-cytotoxic DNMT1 depletion by decitabine in vitro and in vivo is known to bias commitment decisions of multi-potent hematopoietic precursors towards erythroid and megakaryocyte lineage-fate and away from granulocyte-monocyte fate [43,51,53,72]. That is, non-cytotoxic DNMT1 depletion is expected to increase hemoglobin and platelets and concurrently decrease neutrophils. Hemoglobin levels increased in all cohort 4 and 5 (0.08 and 0.16 mg/kg, respectively) patients (in one cohort 4 patient, an isolated large increase in hemoglobin and ANC coincided with a pneumonia diagnosis and dehydration) (Fig 7A, S1 Fig, S1 Data). The total hemoglobin increases from baseline to maximum during 8 weeks of treatment at 0.16 mg/kg dose (cohort 5) were 1.2, 1.8, and 1.9 g/dL (Fig 7A). Rates of increase in total hemoglobin (time-slope estimates) increased with dose (*P* = 0.01) (Fig 7B). Total hemoglobin increased even as reticulocyte counts decreased; that is, better quality and efficiency of HbF-enriched erythropoiesis permitted increases in hemoglobin with fewer reticulocytes (Fig 7C). Also indicating better RBC quality were biomarkers of hemolysis, thrombophilia, and inflammation, namely serum LDH, total bilirubin, D-dimer, and CRP levels, all of which improved in cohorts 4 and 5 (Fig 7D–7G, S1 Fig, S1 Data).

In parallel, as expected with non-cytotoxic DNMT1 depletion, platelets increased concurrently with neutrophil decreases (Fig 7H and 7I). Platelet and neutrophil counts on therapy did not reach a priori–defined triggers for dose modification: neither a platelet count of >1,200 × 10^9^/L, nor an ANC <1.5 ×10^9^/L, were reached, as the highest platelet count on therapy was 1,122 × 10^9^/L in a patient with a pretreatment count of 733 × 10^9^/L, and the lowest ANC on therapy was 1.6 × 10^9^/L in a patient with a pretreatment count of 1.8 × 10^9^/L (Fig 7H and 7I). In contrast to the clear trends in myeloid lineages, there was no clear pattern of change in absolute lymphocyte counts on therapy (S1 Fig).

All blood counts reverted towards baseline 2 weeks after discontinuation of study drug (Fig 7).

## Discussion

This first-in-human clinical trial evaluated safety of oral THU-decitabine, and identified an appropriate dose, for DNMT1 depletion and HbF induction in SCD [70]. Oral THU-decitabine was safe and well-tolerated in this study and produced a wide decitabine concentration-time profile (low C_max_, long T_max_) ideal for non-cytotoxic DNMT1 depletion, since DNMT1 depletion can occur at low nanomolar concentrations but depends on exposure timing [46,73–75]. Increases in fetal and total hemoglobin expected to be clinically significant were produced at the highest decitabine dose administered, 0.16 mg/kg, accompanied by improvements in laboratory biomarkers of hemolysis, coagulation, and inflammation. The side effects were a concurrent increase in platelets and decrease in neutrophils, expected with non-cytotoxic DNMT1 depletion.

The main limitation was narrow ability to document clinical benefits, because this was a first-in-human study with a single-blind design, with only a small number of patients treated at each decitabine dose level, and for only 8 weeks. Non-blinding of investigators could produce over- or under-reporting of AEs. The major rationale for not blinding investigators to treatment assignment was the intent to use intra-patient dose modification for decitabine C_max_ > 0.2 μM, study drug–related ≥ grade 3 toxicity, platelets > 1,200 × 10^9^/L, or neutrophils < 1.5 × 10^9^/L. In the study, intra-patient dose modification was not triggered.

Doses of oral decitabine administered after oral THU were escalated from a very low starting level (0.01 mg/kg), to find the minimal decitabine doses active in depleting DNMT1 without cytotoxicity. Our specific pharmacokinetic objectives were to (i) distribute decitabine through the most CDA-enriched tissues, intestines and liver, which need to be surmounted for oral bioavailability; (ii) extend decitabine half-life/T_max_ to hours, instead of minutes, to increase S-phase–dependent DNMT1 depletion; (iii) avoid off-target cytotoxicity by maintaining C_max_ in the range of >5 nM and <200 nM (nucleotide pool imbalance, DNA-damage, and cytotoxicity correlates with C_max_ > 500 nM (reviewed in [76])). These pharmacokinetic objectives were met: mini-doses of oral decitabine administered 60 minutes after THU 10 mg/kg traversed the intestines and liver to produce systemic exposure with a wide decitabine concentration-time profile (C_max_ approximately 50 nM at 0.16 mg/kg decitabine, T_max_ of hours instead of minutes). By contrast, intravenous administration of decitabine produces a very high C_max_ and very brief half-life of minutes. Although continuous intravenous or subcutaneous infusion can in principle lower C_max_ and extend T_max_, such approaches are impractical, expensive, and do not solve the problem of negligible distribution into CDA-rich solid tissues—target cells residing in such tissues remain unexposed to treatment [62,77,78]. Increasing intravenous or subcutaneous dose is not a solution for uneven tissue distribution, since toxic exposures are produced in sensitive CDA-poor tissues (e.g., bone marrow) while negligible distribution into CDA-rich tissues persists [62,77]. Similarly, attempting to overcome the intestinal/liver CDA first-pass barrier with high oral doses risks luminal drug concentrations toxic to intestinal enterocytes while systemic exposures remain suboptimal [70,79]. Subcutaneous administration blunts C_max_ but does not resolve short half-life and uneven tissue distribution problems [70].

The molecular pharmacodynamic objective of DNMT1 depletion and its corollary, hypomethylation of repetitive element LINE-1 CpG, were produced in peripheral blood mononuclear cells most consistently by oral decitabine 0.16 mg/kg (approximately 5 mg/m^2^). The degree of LINE-1 CpG hypomethylation generated was comparable to that produced by intravenous decitabine regimens FDA-approved to treat myeloid malignancies, that infuse an appoximately four-fold higher intravenous daily dose of 20 mg/m^2^/day for 5 days. However, instead of being pulse-cycled, the hypomethylation with oral THU-decitabine is sustained week to week [80,81]. Also contrasting with intravenous infusion, decitabine exposure was throughout CDA-rich solid tissues, demonstrated by its distribution through intestine and liver into plasma where it was measured. This is clinically pertinent beyond oral bioavailability, since target cells may reside in CDA-rich tissues, e.g., erythroid precursors in the spleen in β-thalassemia [62,77,80].

We previously compared head-to-head in the same non-human primates (*n* = 14) HbF induction by hydroxyurea or cytarabine versus 5-azacytidine (a prodrug of decitabine); 5-azacytidine produced 2 to 20-fold greater increases in HbF than cytarabine or hydroxyurea, without the cytotoxic side effects of cytarabine and hydroxyurea and with consistent effects in older animals less able to respond to cytarabine or hydroxyurea [21]. From this, we repositioned parenterally administered decitabine for non-cytotoxic DNMT1 depletion in SCD patients who had not benefitted from hydroxyurea. In these trials, HbF increases plateaued at >10% from baseline after 12 weeks of treatment, including in patients with essentially 0% HbF increases after 2 years of monitored treatment with hydroxyurea in the pivotal clinical trial [42,43]. The observations here are in line with these previous observations: 8 weeks of treatment with oral THU-decitabine 0.16 mg/kg 2X/week increased HbF 4%–9% from baseline, again in patients who had not benefitted from hydroxyurea. F-cells increased by >2-fold, up to approximately 80% of the total RBC population. Total hemoglobin increased 1.2–1.9 g/dL even as reticulocyte counts decreased, reflecting the better efficiency and quality of HbF-enriched erythropoiesis, also shown by improvements in biomarkers of hemolysis, thrombophilia, and inflammation (LDH, bilirubin, D-dimer, CRP). By way of comparison, transfusion of 1 unit of blood increases hemoglobin by approximately 1 g/dL, and hydroxyurea in the pivotal trial increased total hemoglobin by an average of 0.6 g/dL. Previous parenteral decitabine trials evaluated RBC adhesion to thrombospondin and laminin, RBC hemoglobin concentration, and RBC phosphatidylserine exposure, documenting improvements in these parameters as well [43,51].

In parallel, as expected with non-cytotoxic DNMT1 depletion, platelet counts increased while neutrophil counts concurrently decreased [43,51–53], although remaining within ranges observed in SCD or β-thalassemia patients receiving standard-of-care interventions such as splenectomy or hydroxyurea. Platelet increases and neutrophil decreases are dose/frequency–limiting parameters. In a previous clinical trial in which subcutaneous decitabine was administered up to 3X/week, the more frequent administration, expected to produce DNMT1 depletion in a greater fraction of the erythroid precursor population, did produce even larger increases in HbF and total hemoglobin; however, there were correspondingly greater platelet increases and neutrophil decreases [43]. Platelet increases in SCD, already a thrombophilia, are a concern. The trade-off with better RBC quality, however, is worth closer examination. Several groups have found that platelet activation in β-hemoglobinopathies is by diseased RBC and the endothelial damage they cause [82–94]. In thrombophilic myeloproliferative neoplasms also, thrombotic risk has been found to be a function of qualitative defects in RBC and platelets and not higher platelet counts (reviewed in [95]). Similarly, no link between higher platelets and thrombosis was observed in series of post-splenectomy β-thalassemia patients [91,92], nor during 12–15 years of follow-up of >5,000 normal individuals [96]. The improvement in RBC quality produced by HbF induction thus explains improvements in thrombophilia biomarkers (D-dimer, Von Willebrand factor [vWF] propeptide, RBC adhesion to thrombospondin and laminin, SVCAM1 [endothelial damage biomarker]) in this and previous studies of decitabine, despite concurrent platelet count increases to >800 × 10^9^/L [43]. In short, an association between platelet count and thrombosis risk, though intuitive and possible, is not clear cut in ex and in vivo studies, while qualitative RBC defects, improved by HbF induction, are linked (reviewed in [83]). Future trials of non-cytotoxic DNMT1 depletion will need to continue to carefully monitor the risks/benefits of better RBC quality and higher platelets.

Viscosity that increases with hematocrit also contributes to thrombophilia [97]. That non-cytotoxic DNMT1 depletion produces large increases in total hemoglobin could thus mean that this approach is less suited to SCD subtypes with relatively high hematocrits at baseline, e.g., S-β^+^-thalassemia or S-C disease, even if RBC quality is improved concurrently by HbF induction. This question will need evaluation in future clinical trials.

Another risk is that high concentrations of decitabine can be DNA damaging and mutagenic and hence potentially pro-oncogenic. DNA damage can also be cytotoxic; this is a concern independent of potential oncogenicity because of the extraordinary demands on SCD bone marrow to compensate for severe hemolytic anemia—dwindling of such compensation by vaso-occlusive damage and age contributes to early death [8,23,31,32,98]. A major rationale for oral THU-decitabine, therefore, is creating decitabine pharmacology that reduces off-target anti-metabolite effects, DNA-damage, and cytotoxicity. First, combination with THU decreases formation of uridine degradation products that contribute significantly to off-target DNA damage and cytotoxicity [49,50]. Second, oral THU-decitabine produces a low C_max_–extended T_max_ concentration-time profile that is conducive to DNMT1 depletion without measurable DNA damage or cytotoxicity, shown extensively in vitro (reviewed in [76]), in non-human primates [21,70], and in clinical trials in which very low doses of decitabine were administered subcutaneously by metronomic schedules [43,51,52]; the clinical trials documented non-cytotoxic DNMT1 depletion by a number of assays including bone marrow evaluation of DNMT1 protein levels, cellularity, γH2AX, and sub-G1 fraction, and peripheral blood evaluation of erythrocyte micronucleus and VDJ recombination assays [43,51,52].

Also regarding concerns of oncogenicity, DNMT1 is highly validated as a molecular target to prevent and treat cancer, offering a p53-independent mechanism of action distinct from conventional anti-metabolite therapy and preserving normal dividing cells (excellent therapeutic index) [43,51–54,76,99–103] (reviewed in [104]). These properties of DNMT1 as a molecular target for oncotherapy likely explain why decitabine and the related 5-azacytidine are the only drugs FDA-approved to treat all subtypes of myelodysplastic syndromes, a type of myeloid malignancy usually seen in the elderly in which better outcomes depend on improving blood counts [52,76,99,100,105,106].

Oral THU-decitabine, like standard-of-care hydroxyurea, could be teratogenic and should be restricted accordingly.

Many patients with myeloid malignancies have been treated chronically for years with metronomic very-low-dose decitabine without significant side effects [52]. Although THU is a new chemical entity that is not FDA-approved for any indication, there have been no side effects observed in clinical trials, some with treatment durations up to and exceeding a year (reviewed in [70,107]). The absence of side effects with THU likely reflects the adaptive network structure of pyrimidine metabolism that is robust to inhibition of CDA; THU has been shown to have no effect on nucleotide pool sizes [108]. Also supporting that its inhibition can be non-detrimental to normal physiology is large natural variation in CDA levels between species [109]. Mostly, THU has been used to try to increase anti-metabolite, DNA-damaging effects of coadministered cytotoxic cytidine analogues, an approach that increases systemic toxicity. Here, by contrast, the intent was to create lower C_max_, extended T_max_, more equitable tissue distribution and decrease uridine degradation products of decitabine for non-cytotoxic (non-toxic), molecularly targeted therapy goals (reviewed in [76,99]).

Fetal hemoglobin induction involves chromatin remodeling of the HbF gene locus (*HBG*) [110]. Cytotoxicity generates this remodeling indirectly, via bone marrow stress [21,110,111] (S3 Fig). The inefficiency of this approach versus directly inhibiting epigenetic enzymes is underscored by greater HbF increases produced by <1/1,000th the molar amount of decitabine versus hydroxyurea in the same patients (decitabine approximately 0.2 mg/kg 2X/week versus hydroxyurea approximately 20 mg/kg daily) [43,112] (S3 Fig). Several other drug development efforts are thus also directed towards direct inhibition of epigenetic enzymes that silence HbF (*HBG*). Besides DNMT1, enzymes targeted include histone deacetylases (HDAC), KDM1A/LSD1, and PRMT5. DNMT1-depleting and HDAC-inhibiting drugs are already FDA-approved for other clinical indications. Potential application of marketed HDAC inhibitors for HbF induction is limited by pleiotropic roles of HDAC in cells outside of chromatin, rendering it difficult to separate anti-metabolite/cytotoxicity from epigenetic effects [113–117]. The limitations of marketed parenteral decitabine also apply to 5-azacytidine, a decitabine prodrug. By depleting DNMT1 protein, decitabine and 5-azacytidine disrupt its scaffolding functions for other epigenetic enzymes such as LSD1/KDM1A [118,119]. This potent epigenetic effect, and its known safety profile, justify efforts to improve decitabine pharmacology and accessibility.

HbF is the most powerful known natural modulator of the root cause of SCD pathophysiology. This clinical trial affirms that substantial HbF and total hemoglobin increases can be produced by inhibiting an epigenetic enzyme that mediates HbF silencing and by rationally avoiding cytotoxicity. The oral route of administration and safety profile of the agent used for this purpose, oral THU-decitabine, could have global health implications. Further clinical development and evaluation are thus warranted.

## Supporting information

S1 Data All patient laboratory data. (XLSX)

S1 Fig Changes in hematologic, hemolysis, thrombophilia, and inflammation biomarkers on therapy. Plots of median values in decitabine dose level and placebo cohorts. **(A)** HbF%. **(B)** Total hemoglobin. **(C)** Absolute reticulocyte counts (ARC). **(D)** Serum lactate dehydrogenase levels (LDH). **(E)** Total bilirubin levels. **(F)** D-dimer levels. **(G)** Serum C-reactive protein levels (CRP). **(H)** Platelet counts. **(I)** Absolute neutrophil counts (ANC). **(J)** Absolute lymphocyte counts (ALC). **(K)** Total white blood cell counts (WBC).
(TIF)

S2 Fig F-cells in cohort 5—Original flow cytometry data. Peripheral blood samples were fixed and stained with phycoerythrin-conjugated anti-HbF (Caltag) per manufacturer’s instructions. Analysis on a Becton-Dickinson FacsCalibur (Sunnyvale, CA).
(TIF)

S3 Fig Fetal hemoglobin induction requires chromatin remodeling, including DNA hypomethylation, of the HbF gene locus (γ-globin, *HBG*). Bone marrow stress, e.g., from cytotoxic drugs such as hydroxyurea, can create such remodeling during the recovery phase by surviving erythroid precursors [21,110,111]. An alternative approach is to remodel the HbF locus directly, e.g., by directly inhibiting DNMT1 using decitabine. The relative efficiencies of these approaches are illustrated by the greater HbF increases produced in the same non-human primates or human patients by decitabine approximately 0.2 mg/kg 2X/week, versus hydroxyurea approximately 20 mg/kg daily. That is, the molar amount of decitabine administered per week is <1/1,000th the amount of hydroxyurea administered per week [21,43].
(TIF)

S1 Text Clinical protocol document. (DOC)

S2 Text CONSORT statement. (DOC)

## Abbreviations

AEs: adverse events
ANC: absolute neutrophil count
CDA: cytidine deaminase
CRP: C-reactive protein
DNMT1: DNA methyltransferase 1
F-cells: fetal hemoglobin-enriched red blood cells
FDA: United States Food and Drug Administration
HbA: normal adult hemoglobin
HbF: fetal hemoglobin
HbS: sickle cell hemoglobin
HDAC: histone deacetylases
HPFH: hereditary persistence of fetal hemoglobin
HPLC: high-performance liquid chromatography
LCMS/MS: liquid chromatography tandem mass spectrometry
LINE-1: long interspersed nuclear element
RBC: red blood cell
SCD: sickle cell disease
THU: tetrahydrouridine
