# Supplementary figures and images for: Oral tetrahydrouridine and decitabine for non-cytotoxic epigenetic gene regulation in sickle cell disease: A randomized phase 1 study
Source: PLoS Med. 2017 Sep 7;14(9):e1002382. doi: 10.1371/journal.pmed.1002382 (PMC5589090; doi:10.1371/journal.pmed.1002382)

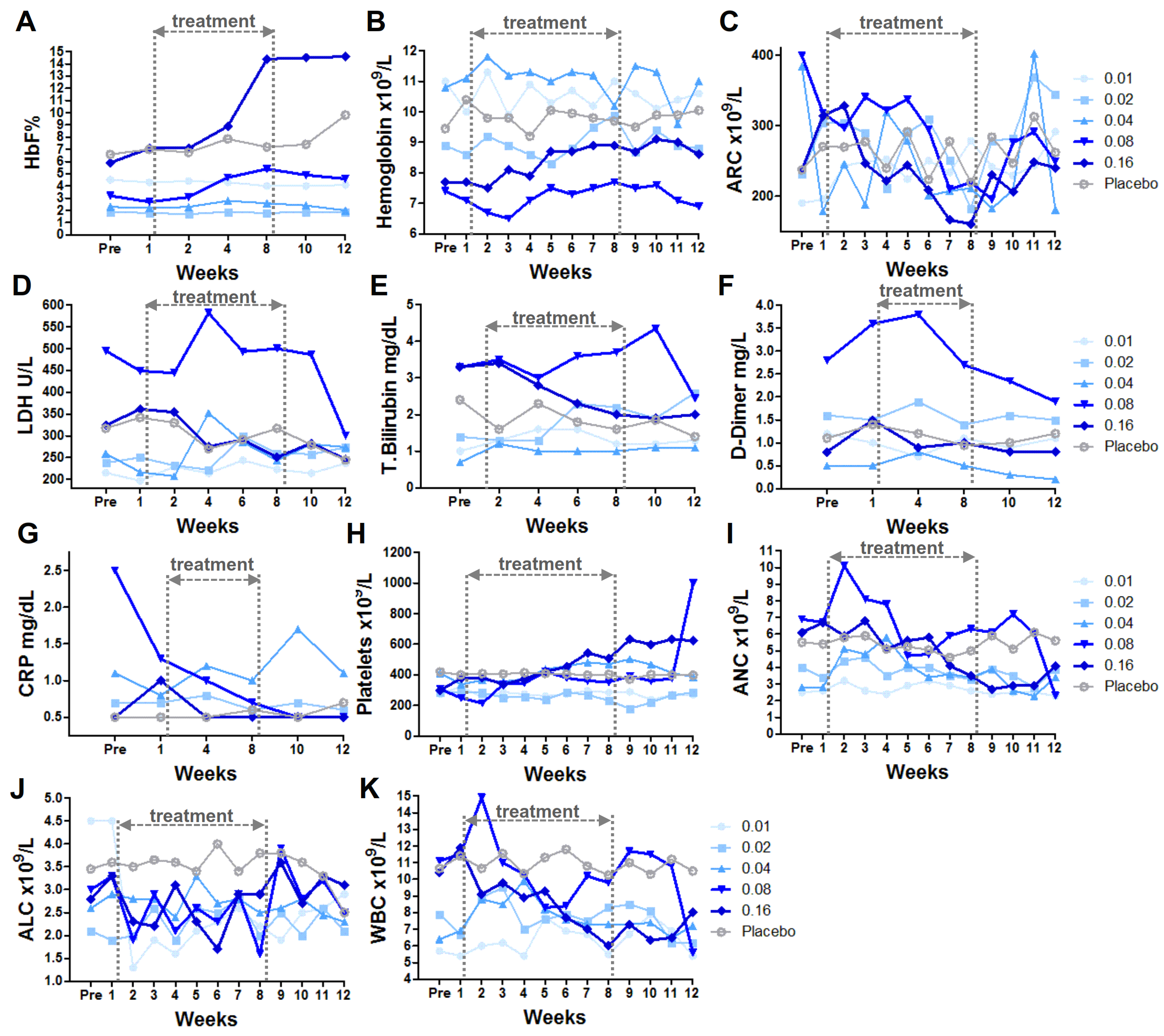

Supplement: S1 Fig — Plots of median values in decitabine dose level and placebo cohorts. (A) HbF%. (B) Total hemoglobin. (C) Absolute reticulocyte counts (ARC). (D) Serum lactate dehydrogenase levels (LDH). (E) Total bilirubin levels. (F) D-dimer levels. (G) Serum C-reactive protein levels (CRP). (H) Platelet counts. (I) Absolute neutrophil counts (ANC). (J) Absolute lymphocyte counts (ALC). (K) Total white blood cell counts (WBC). (TIF) [file pmed.1002382.s002.tif]

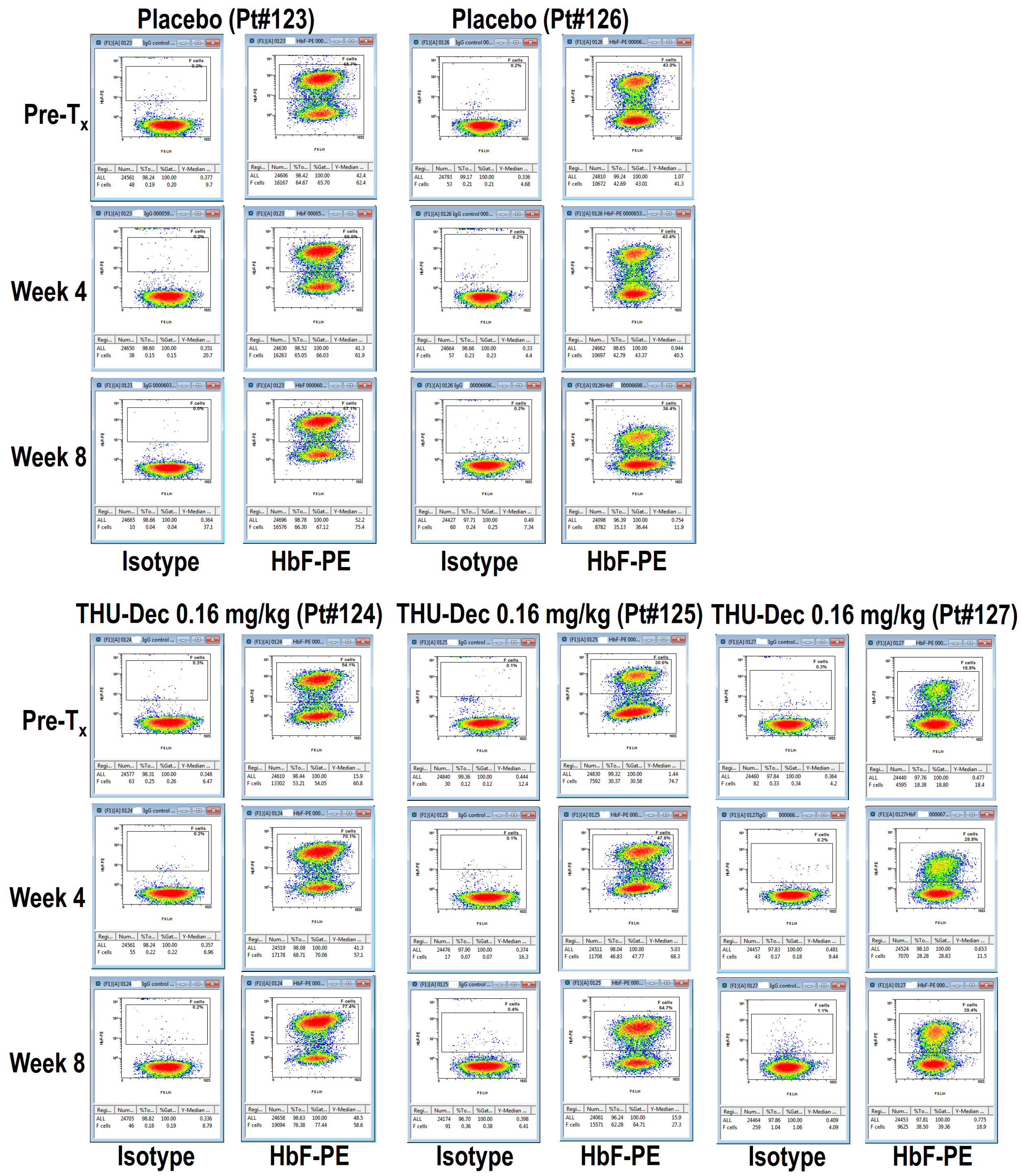

Supplement: S2 Fig — Peripheral blood samples were fixed and stained with phycoerythrin-conjugated anti-HbF (Caltag) per manufacturer’s instructions. Analysis on a Becton-Dickinson FacsCalibur (Sunnyvale, CA). (TIF) [file pmed.1002382.s003.tif]

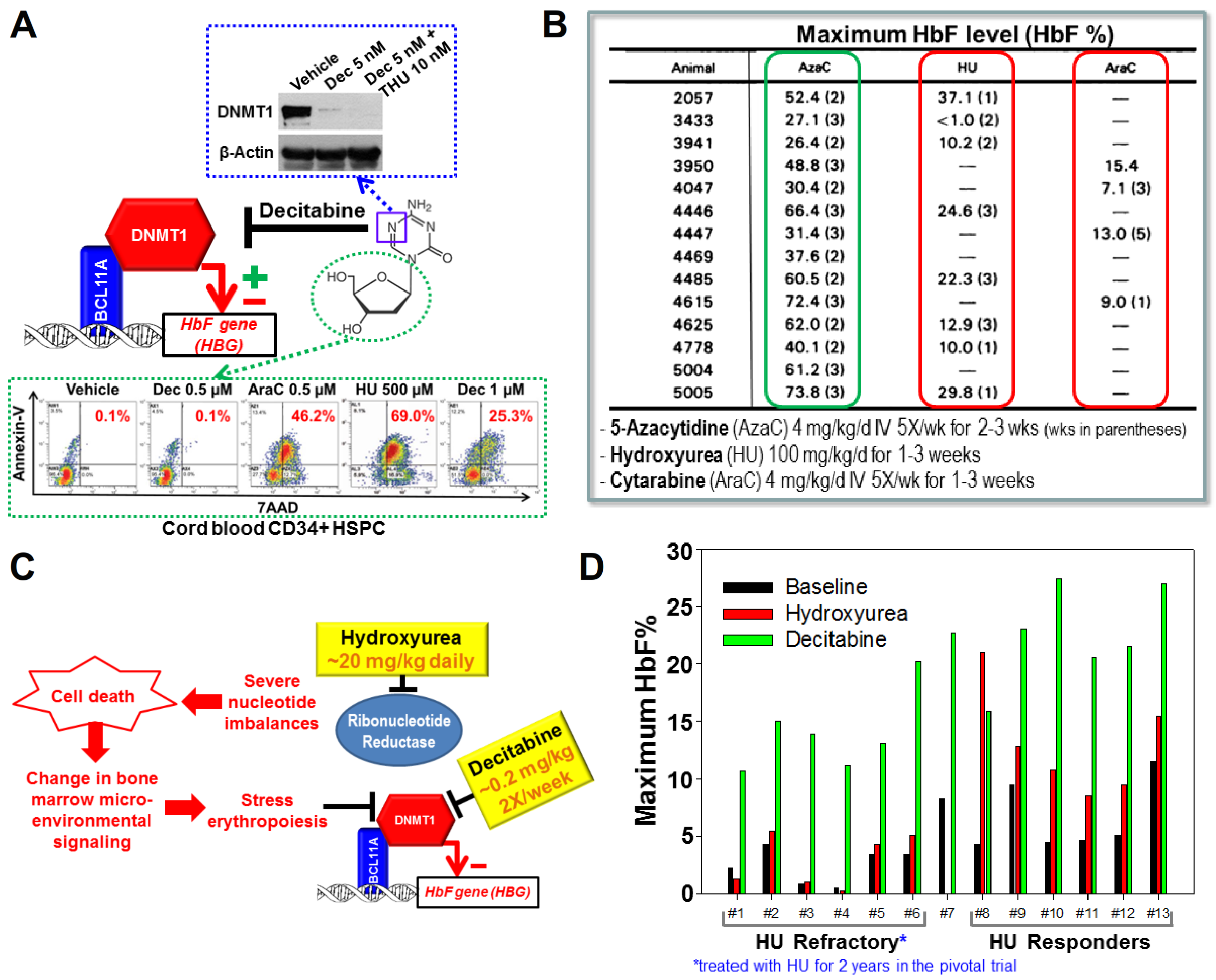

Supplement: S3 Fig — Bone marrow stress, e.g., from cytotoxic drugs such as hydroxyurea, can create such remodeling during the recovery phase by surviving erythroid precursors [21,110,111]. An alternative approach is to remodel the HbF locus directly, e.g., by directly inhibiting DNMT1 using decitabine. The relative efficiencies of these approaches are illustrated by the greater HbF increases produced in the same non-human primates or human patients by decitabine approximately 0.2 mg/kg 2X/week, versus hydroxyurea approximately 20 mg/kg daily. That is, the molar amount of decitabine administered per week is <1/1,000th the amount of hydroxyurea administered per week [21,43]. (TIF) [file pmed.1002382.s004.tif]
