# Supplementary material for: Oral tetrahydrouridine and decitabine for non-cytotoxic epigenetic gene regulation in sickle cell disease: A randomized phase 1 study
Source: PLoS Med. 2017 Sep 7;14(9):e1002382. doi: 10.1371/journal.pmed.1002382 (PMC5589090; doi:10.1371/journal.pmed.1002382)
Supplement: S1 Text — (DOC) [file pmed.1002382.s005.doc]

| CLINICAL PROTOCOL (CASE 10Z11) |
| --- |

| PROTOCOL TITLE: | Phase 1 Study of Oral Decitabine and Tetrahydrouridine (THU) in Patients with High Risk Sickle Cell Disease |
| --- | --- |
| Sponsor-Investigator | Yogen Saunthararajah, MD |
| Version: | 10.0 |
| IND NUMBER: | 112914 |
| DATE: | March 20, 2015 |
| PRINCIPAL INVESTIGATORS FOR EACH STUDY SITE: | Cleveland Clinic:  **Yogen Saunthararajah, MD**  University of Illinois at Chicago:  **Robert Molokie, MD** |
| STATISTICAL ANALYSIS: | **Tomas Radivoyevitch, PhD**, Cleveland Clinic |
| STUDY SITES: | Cleveland Clinic  University of Illinois at Chicago |

1. synopsis

| **Title:** Phase 1 Study of Oral Decitabine and Tetrahydrouridine (THU) in Patients with High Risk Sickle Cell Disease (SCD). |
| --- |
| **Overview:** Survival for SCD patients is 25-30 years less than the African-American population in general ^1^. The ribonucleotide reductase inhibitor drug hydroxyurea (HU) is the only drug currently available for disease modification^2^. The cytostatic/cytotoxic mechanism of action of HU is used to indirectly reactivate fetal hemoglobin (HbF) expression; induced HbF intercalates and interferes with sickle hemoglobin (HbS) polymerization, to increase HbS solubility and prevent sickling^3;4^. However, induction of HbF by indirect, cytostatic/cytotoxic actions of HU is inefficient, and HbF levels are not increased in approximately 40% of HU compliant patients^5-7^. Even in HU-responders, the increase in HbF is often small, a significant limitation since there is a continuous inverse relationship between HbF and pain crisis^8^, early death^1^, and possibly stroke and pulmonary hypertension^7^. Furthermore, HU is used at doses that are genotoxic, teratogenic and testicular toxic, and can compound the bone marrow damage that contributes to early death in SCD^5;9-12^. Methylation of cytosines that precede guanines mediates gene-silencing at many genes including the γ globin gene (*HBG)* that produces HbF^13;14^. The role of DNA methylation and epigenetics in *HBG* silencing explains why the cytosine analogue decitabine, which irreversibly binds and depletes DNA methyl-transferase 1(DNMT1) and thereby hypo-methylates the *HBG* promoter, can increase HbF by large amounts, even in severely ill and HU refractory patients^15;16^. Certain aspects of decitabine pharmacology and mechanism of action influence its clinical activity: unlike cytosine analogues such as cytarabine or gemcitabine, the sugar moiety of decitabine is unmodified. Therefore, at low concentrations, decitabine does not terminate DNA chain synthesis^17;18^, and can deplete DNMT1 without causing significant DNA damage or cytotoxicity, both *in vitro* and *in vivo*^15;17-21^. However, at high concentrations, similar to other nucleoside analogues, decitabine is cytotoxic. Another important aspect of decitabine action is that it is S-phase specific, hence, exposure timing critically influences efficacy**^19;22^**. Considering these properties of decitabine, for the objective of non-cytotoxic DNMT1-depletion, the ideal decitabine concentration-time profile is low peak drug levels but extended time above minimum concentrations required to deplete DNMT1. O**ral administration of decitabine could be more likely to produce this concentration-time profile than parenteral administration, and would have major logistical advantages for the clinical goal of long-term non-cytotoxic epigenetic therapy**. A significant physiologic barrier to decitabine oral bioavailability is the enzyme cytidine deaminase (CDA), that is highly expressed in the gut and liver of humans and mice, and metabolizes cytidine, deoxycytidine and analogues thereof into uridine counterparts that cannot incorporate into DNA**^23-26^**. CDA drastically decreases the half-life of decitabine **to <20 minutes** *in vivo***^27;28^**, from **5-16 hours** **at 37^0^C *in vitro*^27^**. Furthermore, non-synonymous single nucleotide polymorphisms (SNPs) in *CDA* produce person to person variability in CDA enzyme activity^29-32^, and consequently, clinically significant variation in cytosine analogue pharmacokinetics, toxicity and efficacy that could be amplified with oral administration^30;33-36^. The uridine analogue tetrahydrouridine (THU) is a competitive inhibitor of CDA. THU has been used as a CDA inhibitor in combination with cytosine analogues pre-clinically and clinically for some decades, without documentation of toxic side-effects^24;25;37-45^. In both baboons and mice, oral administration of THU to inhibit CDA prior to oral decitabine extended decitabine absorption time and widened the concentration-time profile, reflected in mice by a 9-fold increase in decitabine AUC_total_ compared to a 2.5-fold increase in decitabine C_max_. **Since DNMT1 depletion by decitabine can occur at very low drug levels but depends on exposure timing^22;46-48^, t**he wider concentration-time profile achieved with oral THU-decitabine is suited to the goal of longer time above minimum concentrations required for S-phase specific depletion of DNMT1, while avoiding high peak decitabine levels that can cause DNA damage and cytotoxicity. The baboon model has been accurate and useful in predicting, by body surface area scaling, a safe human equivalent dose for SC decitabine treatment and for combination oral 5-azacytidine and oral THU therapy^15;24;25;39;49-53^. Therefore, **THU** dose (400 mg/m^2^) and timing (60 minutes before decitabine) that are likely to be useful for human translation were identified by studies in baboons. In the baboons, THU also decreased inter-individual variability in pharmacokinetics seen with decitabine alone, another effect that may enhance safety and efficacy. In baboons, repeat administration of oral THU-decitabine using a decitabine dose that would produce peak decitabine concentrations <<0.2µM, was not myelotoxic, hypomethylated *HBG* promoter CpG, and produced large cumulative increases in HbF expression by red cells. In conclusion, the pre-clinical studies suggest that preceding administration of THU substantially increases oral bioavailability of decitabine, favors a concentration-time profile suited to DNMT1 depletion with less cytotoxicity, and decreases inter-individual variability. These properties of the combination could aid the goal of more accessible, safe and efficacious DNMT1-targeted therapy. Hence, this phase 1 clinical trial examines the safety of combination oral THU and decitabine and identifies the dose of decitabine to be used in subsequent phase 2 studies. |
| **Objective:** To identify the dose of oral decitabine that can be safely co-administered with oral THU in a subsequent Phase 2 study. The overall purpose is to develop disease modifying treatment for SCD that is less cytotoxic than the current standard of care, and which can directly and more efficiently reactivate HbF expression by epigenetic mechanisms. |
| **Study Design:** This is a single-blind phase 1 clinical trial with a maximum of five decitabine dose levels, in combination with a fixed dose of THU. Repeat dose, instead of single dose administration, is used to assess safety, increasing the likelihood that the dose that is identified for further studies in this phase 1 study will be safe in phase 2. To enhance safety during conduct of this study, there is intra-patient dose modification, with interruption of study drug administration for laboratory value thresholds that should precede clinical events. Furthermore, information from the preceding dose level is used to select the next dose, hence immediately and efficiently incorporating lessons learned. At each dose level, five subjects are enrolled, with a 3/2 randomization to drug versus placebo, enabling more rigorous statistical and scientific interpretation of results. |
| **Study Population:** The treatment population are adult SCD patients, at risk of early death as defined by published criteria,^54^ despite standard of care HU. Since such patients are not benefiting from HU, participation in this study will not interrupt potentially effective standard of care:   - HbF <0.5 g/dL after ≥6m of HU therapy, OR - 3 or more pain episodes per year requiring parenteral narcotics after >6months of HU therapy, OR - 1 or more acute chest syndrome episodes after ≥6m of HU therapy, OR - Hemoglobin <9 g/dL and ARC ≤250,000/mm^3^ after ≥6m of HU therapy, OR   HU‑intolerant SCD, defined as unable or unwilling to tolerate HU due to hematological or other toxicities. |
| **Intervention and Criteria for Evaluation (End-Points):**   - Dose escalating safety trial with 5 patients per dose level (5 decitabine dose levels: 0.01, 0.02, 0.04, 0.08, 0.16 mg/kg), randomized 3/2 to drug versus placebo with 8 weeks of treatment and 4 weeks of follow-up (to evaluate safety with repeat dose administration). A fixed dose of oral THU is used 10 mg/kg (400 mg/m^2^), based on pre-clinical studies in baboons and previous clinical trials - *Pharmacokinetics*: Blood is collected for pharmacokinetic analysis at 0, 2, 4, and 24 hours after the first decitabine dose (week 1) (see study schema). - *Safety and dose modification*: Q1 wk assessment of blood counts/toxicity before administration of study drug. Hold study drug if hematologic thresholds for dose modification are triggered (ANC <1.5 x 10^9^/L, or platelets >1200 x 10^9^/L), or decitabine C_max_ >0.2 µM, or ≥grade 3 non-hematologic toxicity occurs. After recovery below these thresholds, restart drug with a 25% decrease in dose. If after decrease in dose, toxicity recurs, repeat this process. Consider withdrawal from the study for ≥grade 3 non-hematologic toxicity (judgement to be exercised by treating physician).   *Pharmacodynamics*: Methylation, DNMT1, CDA expression and activity analysis of buffy coat peripheral blood at pre-treatment, week 4, 8, and 10. HbF analysis pre-treatment, week 2, 4, 8, 10 and 12. |
| **Sample Size:** 25 subjects |
| **Human Subjects:** There is a risk of neutropenia, thrombocytosis, and teratogenicity. Patients must take contraceptive precautions to avoid pregnancy during treatment. |

**STUDY SCHEMA**
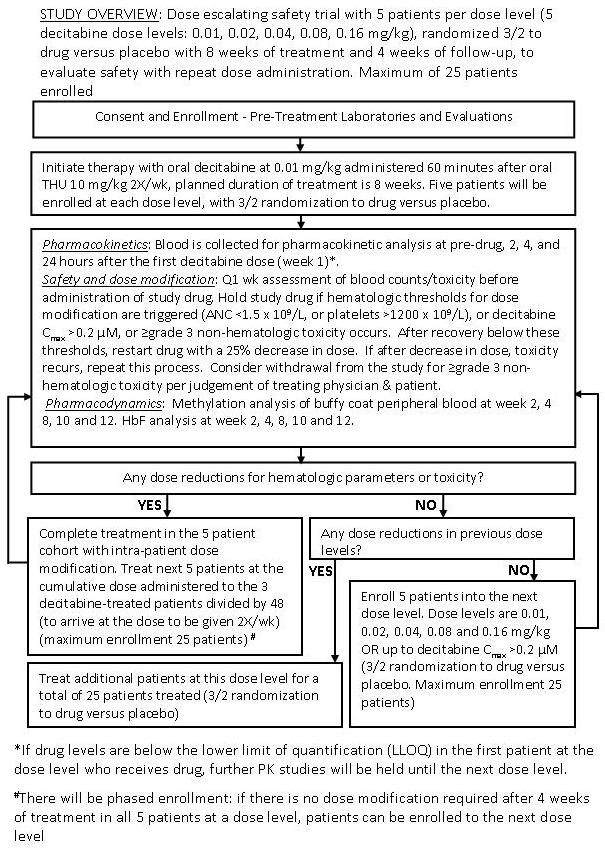


Table of Contents

[**1 synopsis 1**](#__RefHeading___Toc312748359)

Table of Contents 4

2 Abbreviations 6

3 Background, SIGNIFICANCE and Rationale [8](#__RefHeading___Toc312748363)

3.1 Pathophysiology of SCD. 8

[3.2 Principles of Clinical Management 8](#__RefHeading___Toc312748365)

[3.3 SCD and Fetal Hemoglobin (HbF) 8](#__RefHeading___Toc312748366)

[3.4 Mechanism of Action of 5-azacytidine (5-aza-C) and Decitabine in HbF Induction 1](#__RefHeading___Toc312748367)0

[3.5 Thrombocytosis and Other Side-Effects of Decitabine Therapy. 1](#__RefHeading___Toc312748368)0

[3.6 Carcinogencic Risks of Decitabine and Standard of Care HU. 1](#__RefHeading___Toc312748369)1

[3.7 Teratogenic and Reproductive Toxicity Risks of Decitabine and Standard of Care HU 1](#__RefHeading___Toc312748370)3

[3.8 Evidence used to infer safety of starting dose of oral tetrahydrouridine and decitabine 1](#__RefHeading___Toc312748371)3

[3.9 Summary 1](#__RefHeading___Toc312748372)5

[4 Objectives 1](#__RefHeading___Toc312748373)6

[4.1 Objectives 1](#__RefHeading___Toc312748374)6

[5 Study design and METHODS 1](#__RefHeading___Toc312748375)7

[5.1 Study Schema](#__RefHeading___Toc312748376) 18

[5.2 Study End-Points and Hypothesis 19](#__RefHeading___Toc312748377)

[5.3 Selection and Withdrawal of Subjects 19](#__RefHeading___Toc312748378)

[5.4 Inclusion Criteria 19](#__RefHeading___Toc312748379)

[5.5 Exclusion Criteria 19](#__RefHeading___Toc312748380)

[5.6 Stopping Rules/Subject Discontinuation 20](#__RefHeading___Toc312748381)

[5.7 Replacement of study subjects 21](#__RefHeading___Toc312748382)

[5.8 Drug Handling and Administration 21](#__RefHeading___Toc312748383)

[5.9 Timing between oral tetrahydrouridine and oral decitabine administration 22](#__RefHeading___Toc312748384)

[5.10 Timing between drug administrations each week 23](#__RefHeading___Toc312748385)

[5.11 Randomization procedures 23](#__RefHeading___Toc312748386)

[5.12 Starting dose and dose escalation 23](#__RefHeading___Toc312748387)

[5.13 Dose modification of oral decitabine within a cohort 23](#__RefHeading___Toc312748388)

[5.14 Prior and Concomitant Therapy 25](#__RefHeading___Toc312748389)

[5.15 Subject Compliance 26](#__RefHeading___Toc312748390)

[5.16 Pre-Treatment Evaluations 2](#__RefHeading___Toc312748391)6

[5.17 Schedule of On-Study and Follow-up Evaluations and Events 2](#__RefHeading___Toc312748392)7

[5.18 Pharmacokinetic (PK) measurements 29](#__RefHeading___Toc312748393)

[5.19 Safety Evaluations 3](#__RefHeading___Toc312748394)0

[5.20 Efficacy, Pharmacodynamic (PD), and Scientific Evaluations 3](#__RefHeading___Toc312748395)1

[6 Statistical Analysis 3](#__RefHeading___Toc312748396)3

[7 Data MANAGEMENT 3](#__RefHeading___Toc312748397)592012r 17, 2013patient remains.lidayshe 2 day window after screening to treat patient. estigator. Katie will send training slid

[7.1 CRF and Source Documentation 3](#__RefHeading___Toc312748398)4

[7.2 Data Management 3](#__RefHeading___Toc312748399)5

[7.3 Staff Training 3](#__RefHeading___Toc312748400)5

[8 Safety monitoring Plan 3](#__RefHeading___Toc312748401)5

[8.1 Safety Assessments Overview 3](#__RefHeading___Toc312748402)5

[8.2 Adverse Events Definitions 3](#__RefHeading___Toc312748403)5

[8.3 Adverse Event Severity and Relatedness to Treatment 3](#__RefHeading___Toc312748404)6

[8.4 Assessment of Adverse Event Outcome 3](#__RefHeading___Toc312748405)8

[8.5 Serious Adverse Event Reporting; Suspension Guidelines 3](#__RefHeading___Toc312748406)8

[8.6 Subject Discontinuation Due to Adverse Event(s) 4](#__RefHeading___Toc312748407)0

[8.7 Pregnancy Reporting 4](#__RefHeading___Toc312748408)0

[8.8 Protocol Violations 4](#__RefHeading___Toc312748410)1

[9 Subject Compensation 4](#__RefHeading___Toc312748411)1

[10 Protocol Signature Page 4](#__RefHeading___Toc312748412)2

[APPENDIX A: BLOOD SAMPLES FOR GLOBAL METHYLATION, DNMT1, CDA genotype and CDA enZyme activity ANALYSIS (DR. YOGEN SAUNTHARARAJAH Laboratory) 5](#__RefHeading___Toc312748413)0

APPENDIX B: BLOOD SAMPLES FOR PHARMACOKINETIC ANALYSIS…………………...……..51

Appendix C: Blood Samples for MRNA Extraction ………………………………..………52

Appendix D: Clinical Studies with 5-azacytidine in Severe Sickle Cell Disease and β‑Thalassemia 53

[Phase 1/2 Studies of Decitabine in SCD 5](#__RefHeading___Toc312748415)3

[A pilot study of subcutaneous decitabine therapy in patients with β-thalassemia intermedia 5](#__RefHeading___Toc312748416)7

[Chronic Administration of Decitabine in Sickle Cell and Other Diseases 5](#__RefHeading___Toc312748417)8

1. Abbreviations

| 5-azaC | 5-azacytidine |
| --- | --- |
| AE | Adverse event |
| ACS | Acute chest syndrome |
| ALT | Alanine aminotransferase |
| ANC | Absolute neutrophil count |
| ARC | Absolute reticulocyte count |
| AST | Aspartate aminotransferase |
| BSA | Body surface area |
| BUN | Blood urea nitrogen |
| Ca | Calcium |
| CBC | Complete blood count |
| CE | Cloning efficiency |
| CGH | Comparative genomic hybridization |
| CHORI | Childrens Hospital Oakland Research Institute |
| CI | Confidence Interval |
| CpG | Cytosine preceding guanine in genomic DNA |
| CRF | Case report form |
| CRP | C-reactive protein |
| CSSCD | Cooperative Study of Sickle Cell Disease |
| CT | Computerized Axial Tomography |
| CTC | Common Toxicity Criteria |
| CXR | Chest X-ray |
| DNA | Deoxyribonucleic acid |
| DNMT | DNA methyltransferase enzymes |
| EC | Endothelial cell |
| ECM | Extracellular matrix |
| ECOG | Eastern Co-operative Oncology Group |
| EDC | Electronic Data Capture System |
| EKG | Electrocardiogram |
| ER | Emergency room |
| ETOH | Alcohol |
| F1+2 | Prothrombin fragments 1 and 2 |
| FDA | Food and Drug Administration |
| FET | Fisher’s Exact Test |
| GCP | Good Clinical Practice |
| Hb | Hemoglobin |
| Hb SS | Hemoglobin SS Disease |
| HbF | Fetal hemoglobin |
| HbS | Sickle hemoglobin |
| HCO_3_ | Bicarbonate |
| HIV | Human immunodeficiency virus |
| HPFH | Hereditary persistence of fetal hemoglobin |
| HPRT | Hypoxanthine phosphoribosyl transferase |
| HU | HU |
| ICH | International Conference on Harmonisation |
| IND | Investigational New Drug |
| IRB | Institutional Review Board |
| IU | International Units |
| IV | Intravenous |
| K | Potassium |
| kg | Kilogram |
| LDH | Lactic dehydrogenase |
| MCH | Mean corpuscular hemoglobin |
| MCV | Mean corpuscular/cell volume |
| MDS | Myelodysplastic syndrome |
| MedDRA | Medical Dictionary for Regulatory Activities |
| Mg | Magnesium |
| mL | Milliliter |
| MSH | Multi-Center Study of HU |
| Na | Sodium |
| NCE | Normochromic Erythrocytes |
| NCI | National Cancer Institute |
| NHIC | Nursing and Healthcare Informatics Core |
| NHLBI | National Heart, Lung, and Blood Institute |
| NOS | Nitric oxide synthase |
| NYHA | New York Heart Association |
| PBMC | Peripheral blood mononuclear cells |
| PCE | Polychromatic erythrocytes |
| PCR | Polymerase chain reaction |
| PCV | Packed cell volume |
| PI | Principal Investigator |
| PO_4_ | Phosphate ion |
| PRC | Protocol Review Committee |
| RBC | Red blood cell |
| RNA | Ribonucleic acid |
| SAE | Serious adverse event |
| SAP | Statistical Analysis Plan |
| SCD | Sickle cell disease |
| SDMC | Statistics and Data Management Center |
| SF-36 | Quality of Life questionnaire |
| sVCAM | Soluble vascular cell adhesion molecule |
| TAT | Thrombin-antithrombin |
| TCR | T-cell receptor |
| TF | Tissue factor |
| tHb | Total hemoglobin |
| TSP | Thrombospondin |
| UIC | University of Illinois, Chicago |
| VDJ | Region of immunoglobulin gene that is rearranged during B cell development |
| VO | Vaso-occlusion |
| VWFpp | von Willebrand factor propeptide |
| WBC | White blood cell |
|  |  |

1. Background, SIGNIFICANCE and Rationale
   1. Pathophysiology of SCD.

SCD is a genetic disease in which a point mutation at codon 6 of the *β-globin* gene results in a substitution of hydrophobic valine for hydrophilic glutamine in the β-chain of hemoglobin. The abnormal hemoglobin is called sickle hemoglobin (HbS). The less soluble HbS can polymerize in deoxygenated regions of the circulation resulting in red-cell rigidity, red-cell adhesion to endothelium, subsequent inflammatory and coagulation pathway activation and vaso-occlusion — each of these pathophysiological manifestations of SCD ultimately attributable to HbS polymerization. The cardinal clinical manifestations of these pathophysiological processes are anemia, hemolysis and vaso-occlusion that causes recurrent painful episodes and chronic organ damage. A comprehensive approach directed at decreasing hemolysis, increasing hemoglobin and preventing vaso-occlusion while effectively managing acute complications that do occur, offers the best method of management.

**Figure 1: The Pathophysiological Cascade in SCD and points at which therapeutic agents are likely to act (green boxes)**

- 1. Principles of Clinical Management

Disease modification to prevent crises, chronic organ damage and early mortality, and prompt, effective and safe relief of acute crises including pain episodes are the twin pillars of SCD care. As shown in **figure 1**, some treatments address only one aspect of pathophysiology whereas others may have a broader impact. An intervention that may have broad impact is effective induction of HbF to decrease HbS polymerization.

- 1. SCD and Fetal Hemoglobin (HbF)

*Effect of HbF on sickle hemoglobin (HbS) solubility and sickling*: HbF is produced during fetal development and for a short time after birth, although some HbF may persist throughout life. The level of HbF in erythrocytes plays a critical role in determining SCD subject outcomes. The first indication that HbF interferes with complications of the disease was the observation that infants with sickle cell anemia do not begin to develop symptoms until HbF levels decrease to those seen in normal adults ^55^. Then, reports emerged of individuals, mostly in India and Saudi Arabia, who are compound heterozygotes for sickle cell and hereditary persistence of fetal hemoglobin (HPFH). These individuals have 70% HbS in their red blood cells (RBC), but are neither anemic nor symptomatic ^56-58^. The uniform distribution of HbF among their red cells interferes with HbS polymerization, increases its solubility and prevents red cell sickling ^3;4^. Even at lower levels of HbF seen in subjects without HPFH, crisis rate and mortality are inversely proportional to HbF level ^1;8;54;59^. These observations provided the rationale for drug-induced augmentation of HbF to treat SCD.

**Figure 5.1.2: Pathophysiology of vaso-occlusion**

*DNA methylation and HbF expression*: One of the epigenetic changes involved in post-natal γ-globin gene (*HBG*) silencing is DNA methylation. DNA CpG sites in the promoters of β-cluster genes are unmethylated at developmental stages associated with expression and methylated at stages at which they are not expressed, and in non-erythroid cells^13^. In studies of a transgenic human β-cluster, methylation at the *HBG* promoters correlated with post-natal suppression of γ-globin synthesis^60^. The non-human primate Papio Anubis (baboon) demonstrates developmental globin switching almost identical to human: in erythroid cells purified from baboon fetuses of varying gestational ages and from baboon adult bone marrow, DNA methylation changes at the ε-globin genes (*HBE*) and *HBG* gene promoters inversely correlated with gene expression^52^. In an evaluation of human *HBG* and *HBB* promoter methylation in primary human fetal liver and adult bone marrow erythroid cells, promoter methylation and gene expression were inversely related^52^. Domains of DNA hypomethylation spanning thousands of base pairs were observed with developmental stage specific gene activation at the β-globin gene cluster^61^.

*Pharmacologic induction of HbF*: Based on the above observations, there has been pre-clinical and clinical evaluation of drugs that inhibit DNA methyl-transferase 1 (DNMT1) and/or other chromatin modifying enzymes as potential *HBG* activators. A major limitation in the conduct and interpretation of such studies has been the complex or multi-faceted mechanism of action of available drugs. The earliest studies used the cytosine analogue 5-azacytidine, which after partial conversion in vivo into 5-aza-2’-deoxycytidine (decitabine), can incorporate into DNA and bind DNMT1, culminating in the depletion of this key chromatin-modifying enzyme. 5-azacytidine was evaluated in non-human primates, then in humans, for its ability to activate *HBG*, with very promising results^62;63^. However, 5-azacytidine has other actions besides DNMT1 depletion, including anti-metabolite effects from RNA incorporation, and the potential for DNA damage induction and cytotoxicity at high concentrations. These additional actions of the drug gave rise to the suggestion that the observed *HBG* activation could be related to anti-metabolite effects. Hence, it was proposed that drugs not directed at chromatin modifying enzymes, but primarily anti-metabolite in action, could be sufficient to produce clinically important *HBG* reactivation. Furthermore, some pre-clinical studies suggested that 5-azacytidine could increase the risk for cancer^64^, deterring further studies with this compound. Therefore, the ribonucleotide reductase inhibitor hydroxyurea (HU), which was available for oral administration, was evaluated for its ability to activate *HBG*. To some extent validating the idea that anti-metabolite effects were sufficient, these studies were successful and definitive in SCD, with important benefits demonstrated in a randomized controlled trial^2^.

*Limitations of HU for pharmacologic reactivation of HbF*: Although the original Multi-Center Study of HU did not provide definitive evidence that improved outcomes with HU treatment resulted from elevated HbF levels, a long term follow-up study demonstrated that improved survival in these patients correlated with HbF levels ^54^. However, indirect, cytostatic/cytotoxic induction of HbF by HU is inefficient: approximately 50% of the subjects in the pivotal multi-center trial did not respond to this therapy, failing to demonstrate elevated HbF levels after two years of treatment ^5^. Therefore, even with compliance, there can be intrinsic resistance to HbF induction by HU ^65^ ^5-7^. Even in HU-responders, the increase in HbF is often small, a significant limitation since there is a continuous inverse relationship between HbF and pain crisis ^8^, early death ^1^, and possibly stroke and pulmonary hypertension ^7^. Clinical evidence for an effect of HU on stroke and pulmonary hypertension sequelae of SCD is lacking, possibly because the HbF elevations produced are not high enough ^66^ . Furthermore, HU is used at doses that are genotoxic, teratogenic and testicular toxic, and can compound the bone marrow damage that contributes to early death in SCD ^5;9-12^. This may limit the feasibility and effectiveness of chronic myelosuppressive doses of HU ^5;9^ ^5;9;67-69^.

- 1. Mechanism of Action of 5-azacytidine (5-aza-C) and Decitabine in HbF Induction

The cytosine analogs 5-azacytidine (5-aza-C) and decitabine inhibit DNA methylation in mammalian cells. The inhibition is due to the incorporation of these analogs into DNA followed by their covalent binding to and depletion of DNMT1. Decitabine, unlike 5-azacytidine, does not incorporate into RNA and inhibit protein synthesis. Therefore, decitabine is more directly targeted towards the molecular objective of DNMT1 depletion, produces the desired biologic effects at about 1/10^th^ of the dose of 5-azacytidine, and could have fewer side-effects. Unlike cytosine analogues such as cytarabine or gemcitabine, the sugar back-bone of decitabine is physiologic. Therefore, at low concentrations, decitabine does not terminate DNA chain synthesis^17;18^, and can deplete DNMT1 without causing significant DNA damage or cytotoxicity, both *in vitro* and *in vivo*^15;17-21^. Although local hypomethylation at the *HBG* promoter may be contributory, it may not be the only basis for increased HbF with 5-azacytidine or decitabine treatment. Epigenetic changes (DNA methylation and histone alterations) are a feature of hematopoietic differentiation. Therefore, it is not surprising that global DNA hypomethylation by decitabine alters hematopoietic differentiation. In the trials in SCD and *in vitro* conducted with low doses of decitabine, low non-cytotoxic concentrations have been demonstrated to favor erythroid and megakaryocyte production over granulocyte and monocyte production ^70-72^. It is posible that HbF induction by decitabine may be in part due to the favoring of erythroid differentiation, and not only from *HBG* promoter hypomethylation ^73^.

- 1. Thrombocytosis and Other Side-Effects of Decitabine Therapy.

There is substantial information regarding the toxicity of decitabine in humans from clinical trials in patients with relapsed or poor prognosis leukemia and myelodysplastic syndromes. This information is summarized in the package insert for this FDA approved drug. In these studies, the patients received doses much higher than the decitabine exposure planned in this study. Leucopoenia was a major toxicity and nausea, or vomiting were common non-hematologic toxicities. In the pilot studies of decitabine for SCD and β-thalassemia intermedia there were no episodes of NCI/CTEP grade II or higher toxicity. In these SCD and β-thalassemia trials, consistent with a non-cytotoxic mechanism of action, the main side-effect was an increase in the platelet count (thrombocytopenia would be the expected side-effect if the mechanism of action was cytotoxic). Additional assays used to evaluate for cytoxicity and genotoxicity were bone marrow morphological examination, bone marrow DNA content analysis, VDJ recombination assay and erythrocyte micronucleus assay. These assays did not reveal evidence of DNA damage or cytotoxicity. The increase in the platelet count was not associated with any clinical adverse events. Notably, in a SCD study multiple markers of coagulation pathway activity improved^15^. In other words, it is possible that decitabine induced improvements in RBC characteristics could reduce the risk of thrombosis despite concurrent increases in platelets. The exposure levels of decitabine produced by the subcutaneous regimens used to treat SCD and β-thalassemia trials are being used as a benchmark to guide the decitabine dose in combination with THU to be used in this Phase 1 trial.

Thrombocytosis and decitabine: Non-cytotoxic concentrations of decitabine alter hematopoietic differentiation to favor megakaryopoiesis and erythropoiesis. Since patients with sickle cell disease can have thrombo-hemorrhagic complications such as stroke, this side-effect is a concern. Although it is intuitive to expect an association between platelet count and thrombosis risk however, this has not been evident in a number of studies, whereas qualitative RBC defects (or qualitative platelet defects in myeloproliferative disease) from the underlying hemoglobinopathy are highly likely to have a role. Accordingly, in a sickle cell study using subcutaneous administration of decitabine, improvement in multiple indices of RBC pathology was accompanied by improvement in multiple markers of coagulation pathway activity, despite platelet count increases to ~ 1000 x 10^9^/L. Thus, it is possible that decitabine-induced improvements in RBC phenotype could reduce thrombophilia despite concurrent platelet count increases. As such, an overly conservative platelet count threshold for dose modification may hinder optimal decitabine-induced improvement in RBC phenotype with the attendant potential benefits, without necessarily contributing to safety. As such, a platelet count threshold of 1200 x 10^9^/L has been selected as the threshold for dose modification, since this threshold is within the range seen in patients with hemoglobinopathies such as sickle cell disease and thalassemia at baseline, and in individuals post-splenectomy, without a demonstrated cause-effect relationship with thrombosis.

- 1. Carcinogencic Risks of Decitabine and Standard of Care HU.

*Mutagenesis, chromosome instability, and gene expression changes associated with decitabine*: In 10T1/2 cells and V79 cells, decitabine either did not show mutagenicity or was weakly mutagenic. Landolph and Jones (1982) ^74^ concluded that decitabine could be considered negligibly mutagenic. In L5178Y mouse lymphoma cells, decitabine did not produce mutations as measured by 6TG resistance but did induce mutations as measured by tri-fluorothymidine resistance (mutation at the thymidine kinase locus) ^75^. In the Ames Salmonella Microsome test, decitabine was not mutagenic (RCC, 1991). In colonic cells from mice transgenic for E. Coli lac I, analysis of the transgene demonstrated mutations predominantly at CpG nucleotides. Toxicity appeared to be the result of DNA methyl-transferase (DNMT) trapping rather than DNA hypomethylation ^76^ ^77^. It was shown that after incorporation into DNA, decitabine binds covalently to DNMT. In a Salmonella typhimurium tester strain TA100, decitabine acted as a weak mutagen ^78^.

Profound hypomethylation of repetitive element DNA produced by knocking out the DNMT genes causes chromosome instability and tumorigenesis in mouse models ^79;80^. However, the DNA hypomethylation induced by short-term treatment with decitabine is considerably less than that seen in the mouse DNMT knock-outs ^81^. In subjects with myelodysplastic syndrome (MDS) treated with decitabine, no unusual patterns of cytogenetic instabilities were found that would suggest that the drug was exacerbating chromosome instability ^82^. Unlike murine knock-out models, the DNMT1 depletion produced by decitabine is temporary.

Microarrays were used to identify genes activated by decitabine treatment of tumor cell lines. Increased expression was limited to relatively few genes (0.67% of 25 940 genes analyzed) ^83^. Since decitabine is a global DNA hypomethylating agent, decitabine treatment could, in theory, reactivate genes that may favor cellular transformation. In practice, however, decitabine has been noted to reactivate expression of a range of tumor suppressor genes that counter cellular transformation (reviewed in^84^). This reactivation of tumor suppressor genes may partly explain the chemopreventive effect of decitabine in a number of animal models of cancer. Hypomethylation at the local gene promoter may not be the only mechanism by which decitabine results in the expression of a gene. Hypomethylation in the local promoter may be insufficient for gene expression, since the relevant transcription factors must also be present ^83^.

The histone deacetylase inhibitors are another group of agents that act at the epigenetic level to alter gene expression. This has been confirmed with microarray analysis of genes up- or down-regulated by histone deacetylase inhibitor treatment of tumor cell-lines, with between 2-10% of analyzed genes up-regulated ^85-87^. Members of this class of agent, such as butyric acid and sodium valproate, have been used for many decades in the therapy of subjects with central nervous system disorders and inherited metabolic diseases, without reported data to suggest that they cause secondary malignancies.

*Possible mechanism by which hypomethylating agents prevent or treat cancer:* Tumor suppressor genes are genes which inhibit tumor growth. These genes are silenced or removed in cancer cells. Up regulation of tumor suppressor genes may be useful in inhibiting neoplastic transformation. A growing number of cancer genes are being recognized that demonstrate dense methylation in normally unmethylated promoter CpG islands. In other words, DNA methylation is an important method by which tumor suppressor genes are silenced during neoplastic evolution ^88;89^. Treating tumor cells with hypermethylated p16 promoters with decitabine restored function of this tumor suppressor gene ^88^, suggesting that decitabine might have a role in the treatment or prevention of cancer. Indeed, as described below, decitabine has been chemopreventive in a number of animal models of cancer and has been used clinically to successfully treat myelodysplastic syndrome, a disease which is characterized by cytogenetic instability.

*Carcinogenesis by decitabine in animal models:* There are six publications which we know of in which decitabine decreased the incidence to cancer in animal models:

Mice carrying the Min gene develop multiple intestinal adenomas. When treated with decitabine, these mice do not develop tumors ^90^. In a primary mouse lung tumor model, decitabine produced a 23% reduction in tumor incidence and a 42% reduction in tumor multiplicity induced by 4-methyl-nitrosamino-1-3-pyridyl-1-butanone exposure ^91^. In the Fisher rat testicular cancer model, decitabine decreased testicular cancer occurrence to 0% compared to a 20% incidence in controls ^92^. Decitabine treatment reversed the immortal phenotype in an early oral cancer model ^93^. In mice with a mutation in a tumor suppressor gene, decitabine decreased the incidence of cancers by 30%. A histone deacetylases inhibitor alone did not reduce the cancer incidence ^94^.

There is one publication and one abstract that we know of in which decitabine increased the incidence of cancer in an animal model:

Decitabine promoted goitrogen induced, but not radiation induced, thyroid carcinoma in a mouse model ^95^. In female Sprague Dawley rats, decitabine increased the rate of malignant tumor formation from 30-40% in controls to between 74-94% in rats treated with 6mg/kg of decitabine (Berger MR**.** Proc Am Ass Cancer Res 1997;38:599 (abstract)).

In over 1,000 patients treated with decitabine in clinical trials for either hematologic or solid tumors, there have been no reports of secondary malignancies.

*Chromosome Instability Secondary to DNA Hypomethylation:* Profound hypomethylation of repetitive element DNA produced by knocking out the DNA methyl-transferase (DNMT) genes causes chromosome instability and carcinogenesis in mouse models ^80;80^. However, the DNA hypomethylation induced by short-term treatment with decitabine is considerably smaller in magnitude than that seen in the mouse DNMT knock-outs ^81^. Futhermore, the DNMT1 depletion and DNA hypomethylation produced is temporary. In patients with myelodysplastic syndrome treated with decitabine, an examination for unusual patterns of cytogenetic instability was negative ^82^.

*Carcinogenic Risks of standard of care with HU:* HU is genotoxic in a wide range of test systems and is thus presumed to be a human carcinogen. In patients receiving long-term HU for myeloproliferative disorders, such as polycythemia vera and essential thrombocythemia, secondary leukemia has been reported. It is unknown whether this leukemogenic effect is secondary to HU or is associated with the patients' underlying disease. Skin cancer has also been reported in patients receiving long-term HU. Conventional long-term studies to evaluate the carcinogenic potential of HU have not been performed. However, intraperitoneal administration of 125-250 mg/kg HU (about 0.6-1.2 times the maximum recommended human oral daily dose on a mg/m2 basis) thrice weekly for 6 months to female rats increased the incidence of mammary tumors in rats surviving to 18 months compared to control. HU is mutagenic in vitro to bacteria, fungi, protozoa, and mammalian cells. HU is clastogenic in vitro (hamster cells, human lymphoblasts) and in vivo (SCE assay in rodents, mouse micronucleus assay). HU causes the transformation of rodent embryo cells to a tumorigenic phenotype. The mutagenic and teratogenic potential of HU is described in the drug package insert and is well-known and therefore is not reviewed in detail here. In SCD, HU is used at doses that cause acute DNA damage and cytotoxicity. In contrast, the proposed dose and schedule of decitabine in this study is intended to be non-DNA damaging and non-cytotoxic.

- 1. Teratogenic and Reproductive Toxicity Risks of Decitabine and Standard of Care HU

Both HU and decitabine are classified as pregnancy category D. Therefore, a number of measures are taken in this proposal to prevent conception or pregnancy during treatment with decitabine.

A concern with chronic administration of decitabine to patients with sickle cell disease is that just like HU (the current standard of care for patients with symptomatic sickle cell disease), decitabine may be teratogenic and have effects on spermatogenesis. This is a special concern since many of these patients will be of reproductive age. These concerns are identical for HU, a known teratogen with effects on spermatogenesis, which is the current standard of care for the targeted group.

In preclinical studies decitabine was a teratogen with one of its teratogenic effects being affected reproductive behavior and performance in off-spring. When administered to pregnant mice, decitabine resulted in increased fetal defects that are both related to dose and gestation day of treatment. Fetal defects included axial and cranial malformations, digital abnormalities and effects on long bones ^96^. In rat embryos, decitabine toxicity was characterized by fetal resorption and fetal defects generally similar to those seen in mice ^97^. Additionally, in utero exposure to decitabine resulted not only in teratogenesis and reduced fetal size, but also continued weight suppression in animals born after decitabine administration. This effect appeared more prominent in male than female offspring and may be related to the changes in hormone levels. In utero exposure to decitabine resulted in decreased fertility in off-spring, primarily in males^98^. Effects in off-spring exposed in-utero included testicular atrophy and altered reproductive behavior.

In mice, decitabine produced a dose-dependent decrease in testicular weight, decline in sperm counts and reduced fertility ^99^.

In view of the teratogenic and other reproductive effects of decitabine in rodents, decitabine should not be used in pregnant women or women with childbearing potential, as well as in men whose partners are of childbearing potential. In addition, men are advised not to father a child while receiving treatment with decitabine and for 2 month afterwards.

Based on the teratogenic risks of decitabine, this study has strict rules regarding contraception for both males and females, frequent pregnancy tests and recommendations for avoiding reproduction for two months after discontinuation of study drug. Reproductive concerns are described in the consent form.

HU was approved by the FDA in 1967 and more information on HU is available in the package insert.

Decitabine was approved by the FDA in 2006 and more information is included with the package insert.

- 1. Evidence used to infer safety of starting dose of oral tetrahydrouridine and decitabine

**3.8.1. Fixed dose of THU**: The proposed starting dose of oral THU is 10 mg/kg (~400 mg/m^2^) without dose escalation (fixed THU dose). This THU dose is based on non-human primate studies, in which the effect of THU dose on decitabine pharmacokinetics were examined in baboons, and results from multiple studies of THU administration in humans^25;37-42^ ^43^ ^44^(summarized in Investigators Brochure). Numerous pre-clinical and clinical studies with THU have not identified any toxic side-effects of this drug^25;37-42^ ^43^ ^44^ ^24^ ^45^.

**3.8.2. Starting dose of decitabine**: The proposed starting dose of decitabine is 0.01 mg/kg (0.35 mg/m^2^). The safety of this proposed decitabine starting dose is inferred from the following:

**(i)** *Clinical trial data*: Administration of decitabine to SCD and β-thalassemia patients by the SC route at 0.2 mg/kg (7.5 mg/m^2^) 2-3X/week for 12 weeks or longer was not cytotoxic as measured by peripheral blood counts, bone marrow cellularity, bone marrow flow cytometry for sub-G1 DNA content, erythrocyte micronucleus assay and VDJ recombination assay in two clinical trials (n=13), one of which was a multi-center trial^72;100^. These studies in humans provide a benchmark for exposure to decitabine that is non-cytotoxic. The proposed starting oral decitabine dose of 0.01 mg/kg is a 20-fold reduction from this SC decitabine dose, to provide the following safety-margin: even if co-administration of THU produces 100% oral bioavailability* to mimic SC or IV administration of 0.01 mg/kg, and in addition, increases AUC_last_ compared to decitabine 0.01 mg/kg alone by 10-fold^#^, the resulting decitabine exposure with this starting oral dose would approximate administration of decitabine 0.1 mg/kg SC, 2-fold less than the current SC decitabine regimens.

*Cytosine analogue oral bioavailability with THU compared to the same dose administered IV has been examined in mice: THU co-administration increased oral bioavailability of gemcitabine from ~10 to ~40%**^26^.**

**^#^**I**n both baboons and mice, THU co-administration increased AUC_last_ approximately 9-fold compared to oral decitabine alone [Investigators Brochure]).**

**(ii)** *Concentration range of decitabine that depletes DNMT1 without causing cytotoxicity*: *In vitro*, concentrations of decitabine below 0.2-0.5 µM (half-life 5-16 hours**^27;101^**) have been demonstrated to deplete DNMT1 without causing measurable DNA damage or cytotoxicity: (a) single exposure to decitabine 0.4 µM decreased DNA methylation but did not decrease viability of Friend erythroleukemia cells^48^; (b) Daily treatments for 2-3 days with decitabine 0.3 µM profoundly decreased DNMT1 protein expression but did not decrease the viability of KG1a or THP1 acute myeloid leukemia (AML) cells. This treatment did not increase phosphorylation of H2AX in THP1 cells (a marker of DNA damage), although in KG1a cells, an increase in phospho-H2AX was noted with 3 days of treatment^102^. (c) Two daily treatments with decitabine 0.2 µM did not cause DNA damage in 8 melanoma cell lines, measured by multiple assays: no phosphorylation of H2AX, CHK1 or p53, no BAX or GADD45 induction, no positive comet assay^21^. (d) Three daily treatments with decitabine 0.1 µM (the highest concentration used by these investigators) was not cytotoxic to normal CD34+ hematopoietic stem and progenitor cells^103^. (e) Two daily treatments of normal CD34+ hematopoietic stem and progenitor cells with 0.5 µM of decitabine substantially depleted DNMT1 but did not increase phospho-H2AX, DNA scission or apoptosis (Investigators Brochure).

**(iii)** *Toxicity study in CD-1 mice (no NOAEL)*: To determine pharmacokinetic-toxicology relationships during repeat dose administration, a toxicokinetic study was conducted in CD-1 mice administered oral decitabine in combination with THU by oral gavage 2X/week for 9 weeks, with a 28 day recovery period. The dose of THU was fixed at 167 mg/kg (400 mg/m^2^), decitabine dose was 0.2, 0.4 or 1.0 mg/kg. There was a linear correlation between dose of decitabine, C_max_ and AUC _cal,0→180_. These pharmacokinetic values were higher in females than males. There was no increase in decitabine C_max_ and AUC _cal,0→180_ on day 58 (week 9) compared to day 1 (there was a decrease of ~30%). The most sensitive tissues were bone marrow, lymphoid and testicular tissue. At the low and mid-dose decitabine levels (0.2 and 0.4 mg/kg), which produced C_max_ 1.5 and 2.9 µM respectively, there was reversible toxicity in these sensitive organs. At the decitabine high dose level (1.0 mg/kg), which produced C_max_ 8.6 µM in female mice, there was lethal toxicity in eight females and one male. Most deaths occurred between study days 32 and 36, cause of death was septicemia secondary to bone marrow suppression. Hematologic parameters correlated with bone marrow hypocellularity. Only testicular findings were observed at the end of the recovery period, with evidence of recovery towards resolution. In brief, the toxicities seen with combination oral THU-decitabine resembled the toxicities seen with exposure to decitabine alone at higher doses ^104^, and no unexpected toxicities were observed with combination therapy.

**(iv)** *Pharmacokinetic and pharmacodynamic studies in non-human primates*: The murine studies enabled more comprehensive analyses of the effects of THU on decitabine pharmacokinetics, and are useful for examining relationships between pharmacokinetics, pharmacodynamics and toxicity (toxikinetics). However, dose-exposure extrapolation by body surface area scaling from mice to humans is not useful, since there is more than 100-fold greater decitabine exposure in rodents versus human dose for dose^105^. Similarly, there is more than 100-fold greater exposure of the cytosine analogue cytarabine in mice versus monkey dose for dose^106^. The reasons for this log-scale increase in cytosine analogue exposure in rodents compared to primates are unknown^24;25;39;107^(Investigators Brochure). Therefore, baboon pharmacokinetic studies were conducted to provide guidance for human equivalent dose by body surface area scaling, since such scaling has been clinically safe and useful in clinical trials of combination oral 5-azacytidine/THU and decitabine IV and SC to treat SCD^25;39;49-53;72^. In baboons, oral decitabine 100 mg/m^2^ (human equivalent dose 2.7 mg/kg) in combination with oral THU (400 mg/m^2^) produced decitabine C_max_ ~0.2 µM in 1 of 7 animals. There was a linear correlation between decitabine dose and C_max_. Therefore, if baboon decitabine-THU pharmacokinetic data can be scaled to humans, the human starting dose of oral decitabine 0.01 mg/kg in combination with THU is expected to produce C_max_ up to 0.00074 µM. This C_max_ is 1350-fold lower than the level (>1.0 µM) that causes measurable DNA damage and apoptosis in normal hematopoietic stem and progenitor cells, is 11,486-fold lower than C_max_ 8.6 µM that was lethal with repeat exposure in murine toxicology studies (Investigators Brochure) and 3,920-fold lower than C_max_ 2.9 µM that produced tolerated, non-lethal, reversible toxicity with repeat exposure 2X/week for 8 weeks in murine toxicology studies (Investigators Brochure).

If unexpectedly oral decitabine/THU dose-pharmacokinetic correlations scale from mice to human by body surface area, then oral decitabine 0.016 mg/kg co-administered with THU in humans might produce C_max_ ~1.5 µM. To ensure safety in case of this possibility, decitabine pharmacokinetics are measured after single dose oral administration of decitabine and THU. If C_max_ is >0.2 µM with the proposed starting dose of oral decitabine 0.01 mg/kg, the next dose will be reduced by 10-fold to 0.001 mg/kg. Supporting the safety of this approach, C_max_ up to 2.7 µM is produced repeatedly on a routine basis by human regimens approved by the FDA to treat myelodysplastic syndrome (15 mg/m^2^ [0.4 mg/kg] infused IV over 3 hours Q8 hrs D1-3 repeated every 6 weeks or 20 mg/m^2^ infused IV over 1 hour daily D1-5 repeated every 28 days^105;108;109^) and in mice, repeat exposure to oral decitabine/THU at the mid-dose level 2X/week for 8 weeks with C_max_ 2.9 µM produced non-lethal reversible cytotoxicity. The treated patient group is not naïve to cytotoxic exposure, since they would have received cytotoxic standard of care.

The monitorable and sentinel toxicities identified in toxicology studies will be closely followed. Therapy will be held if toxicity is detected. In baboon studies, oral decitabine 5-10 mg/m^2^ in combination with oral THU 400 mg/m^2^ administered 2-3X/week for up to 8 weeks did not produce hematologic or other evidence of cytotoxicity (Investigators Brochure).

- 1. Summary

Since 1995, the standard of care for patients with symptomatic SCD has been HU used at DNA damaging and cytotoxic dose and schedule. HU is also pregnancy category D. Peer-reviewed published pre-clinical and clinical data suggests that decitabine could be considerably more efficacious, and safer, than HU for HbF elevation, and has the potential to be a superior agent for chronic disease modification of SCD. Surrogate clinical endpoints such as HbF levels, red-cell adhesion, coagulation system activation and endothelial damage, have improved in all patients treated in early phase clinical trials. HbF levels of >30% may be necessary to prevent or treat pulmonary hypertension and the risk of stroke in subjects with sickle cell disease ^110^. Decitabine, with chronic weekly dosing to produce cumulative increases in HbF, has the potential to produce such elevations, more so than HU (**Figure 2**). Additionally, the off-label experience in patients too severely ill to qualify for protocol therapy is a strong indicator of tolerability and likely effectiveness ^16^.

Unfortunately, in its current formulation, decitabine must be administered by the subcutaneous or intravenous (IV) routes in the clinic (hydrolysis of decitabine after reconstitution precludes self-injection at home). These logistics prevent access or practical treatment for most SCD patients, especially for long-term disease modification. **Furthermore, oral administration could have safety and efficacy advantages over parenteral administration of decitabine, since the desired pharmacologic profile is to avoid high peak drug levels (>1.0 µM) that can cause DNA damage and cytotoxicity but increased time-above-threshold concentration of decitabine (~0.005 µM) for depleting DNMT1 ^15;17-22;48^. For the purposes of oral administration, we propose to combine decitabine with tetrahydrouridine (THU), a competitive inhibitor of the enzyme cytidine deaminase (**CDA) ^23;111;112^. This combination is proposed for the following reasons: **(i) CDA severely limits oral bioavailability of cytosine analogues ^24;25^ ^23^**. **(ii) CDA severely abbreviates decitabine half-life to <20 minutes ^27;28^; since DNMT1 depletion by decitabine can occur at very low drug levels, but depends on exposure time ^22;46-48^, this short *in vivo* half-life impedes the clinical translation of promising *in vitro* results. (iii)** Non-synonymous single nucleotide polymorphisms in *CDA* produce major person to person variability in CDA enzyme activity ^29-32^, and consequently, clinically significant variation in cytosine analogue pharmacokinetics, toxicity and efficacy ^30;33-36^. **(iv)** THU has been extensively studied pre-clinically and clinically, and no toxic side-effects have been identified ^25;37-42^ ^43^ ^44^ ^24^ ^45^.

Repeat dose, instead of single dose administration, is used to assess safety, increasing the likelihood that the dose that is identified for further studies in this phase 1 study will be safe in phase 2. To enhance safety during conduct of this study, there is intra-patient dose modification, with interruption of study drug administration for laboratory value thresholds that should precede clinical events. Furthermore, information from the preceding dose level is used to select the next dose, hence immediately and efficiently incorporating lessons learned. At each dose level, five patients are enrolled, with a 3/2 randomization to drug versus placebo, enabling more rigorous statistical and scientific interpretation of results.

The question of decitabine use in SCD relates to its risk-benefit ratio in this population. The unknown risks of treating SCD with decitabine must be balanced against the known large risks of early mortality and chronic morbidity ^1^ which may be ameliorated with decitabine treatment. HU has been a great advance for our patients. Decitabine offers the possibility of even greater and more wide-spread benefit, possibly with a similar or better toxicity profile. This can only be determined through the conduct of clinical studies.

Figure 2: Decitabine (DAC) has produced marked elevations in fetal hemoglobin (HbF) levels in all SCD subjects treated thus far. Subject numbers 1-6 had minimal or no response to HU (HU) with compliance documented by drug levels. Subjects 8-13 responded to HU with HbF elevations. Subjects 6 and 8 received lower daily and cumulative doses of DAC than the other subjects. HbF levels in subject 7 were not measured during HU therapy.

1. Objectives
   1. Objectives

The primary objective is identify the dose of oral decitabine that can be safely co-administered with oral THU in a subsequent Phase 2 study. This is defined as the dose of oral decitabine, that when administered together with oral THU on a weekly basis over 8 weeks, has a <30% chance of requiring dose modification. The overall purpose is to develop disease modifying treatment for SCD that is less cytotoxic than the current standard of care, and which can directly and more efficiently reactivate HbF expression by epigenetic mechanisms.

The secondary objectives are to characterize the pharmacodynamic effects of THU and decitabine with regards to cytidine deaminase enzyme activity, DNMT1 depletion, DNA methylation and fetal hemoglobin activation, and effects of therapy on efficacy parameters such as sickle cell crisis frequency, coagulation pathway activity and inflammatory pathway activity.

1. Study design and METHODS

This is a single arm single-blind phase 1 clinical trial that proceeds through a maximum of five dose-levels of oral decitabine with a fixed dose of oral THU.

- 1. Study Schema


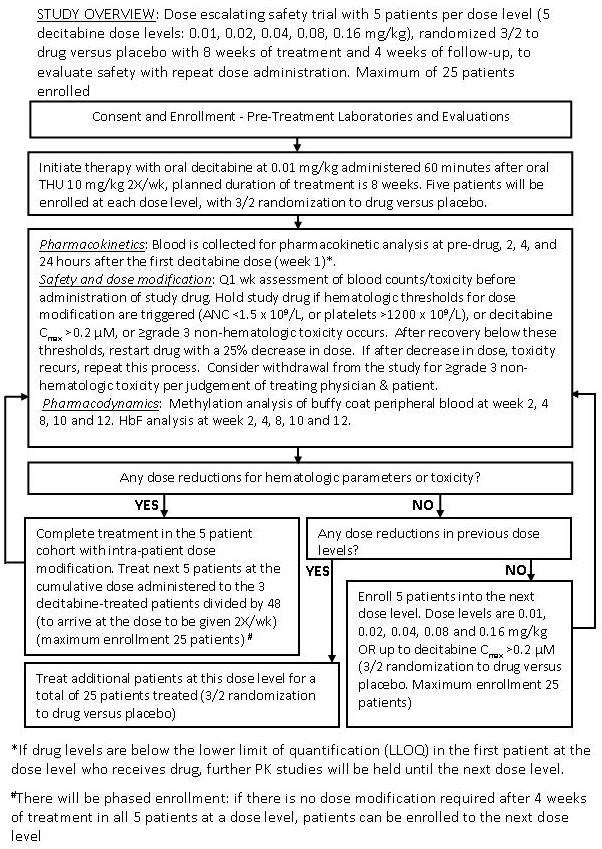


- 1. Study End-Points and Hypothesis

The primary end-point is ≥ grade 3 non-hematologic toxicity. Our hypothesis is that patients in the treatment groups receiving oral THU-decitabine 2X/week over 8 weeks (n=15) will be equivalent to placebo group (n=10) with regards to the chance of ≥ grade 3 non-hematologic toxicity.

Secondary end-points to evaluate pharmacodynamics (PD) and efficacy are **(i)** sickle cell crisis frequency (efficacy); **(ii)** coagulation (D-dimer) and inflammatory (CRP) pathway activity (efficacy); **(iii)** Fetal hemoglobin (HbF) levels measured by HPLC (efficacy and PD of decitabine); **(iv)** DNA methylation levels at repetitive elements in buffy coat DNA (PD of decitabine); **(v)** DNMT1 levels in buffy coat cells (PD of decitabine); **(vi)** Cytidine deaminase (CDA) genotype (pharmacogenetic variable that influences CDA activity); **(vii)** CDA functional activity in serum (PD of THU).

- 1. Selection and Withdrawal of Subjects

. SCD is an orphan disease. Moreover, this clinical trial administers placebo or study drug to the subset of this orphan population who are at high risk of early death. The disease process in these at-risk subjects causes a high rate of adverse events and hospital admissions. This activity and acuity of disease further limits the possibility of clinical trial participation. To facilitate answering the scientific and clinical question that pertains to this highly restricted and at need patient population efficiently without compromising safety or scientific integrity, patients who complete placebo treatment at a dose level are eligible to be screened and randomized to either placebo or study drug in a subsequent dose level. This cross-over feature also offers the opportunity for placebo patients to potentially receive the investigational product. Potential enrollees will be identified by investigators at the clinical sites per the inclusion and exclusion criteria. Patients who are re-enrolled must have been previously enrolled and randomized to a placebo arm of the study.

- 1. Inclusion Criteria

The treatment population are adult SCD patients who remain at risk of early death as defined by published criteria,^54^ despite standard of care HU. Since such patients are not benefiting from HU, participation in this study will not interrupt potentially effective standard of care:

Subjects who meet all of the following criteria are eligible for enrollment into the study:

1. Age 18 years or older.

2. Written, informed consent provided by the subject before study entry.

3. Confirmed SCD (SS, S-β^0^-thalassemia, S-β^+^-thalassemia or SC on hemoglobin electrophoresis),

4. Symptomatic SCD while on 6 months of HU OR symptomatic SCD and intolerant of HU (unable or unwilling to take HU due to hematological or other toxicities). Symptomatic SCD is defined as having one of following:

• HbF <5%, OR

• 3 or more pain episodes per year requiring parenteral narcotics, OR

• 1 or more acute chest syndrome episodes, OR

• Hemoglobin <9 g/dL and absolute reticulocyte count <250,000/mm^3^.

5. Subject is in his/her steady state and not amidst any acute complication due to SCD (i.e., hospitalization, acute pain, or acute chest syndrome in the past 14 days).

6. Regular compliance with comprehensive care and previous therapy.

- 1. Exclusion Criteria

Subjects who meet any of the following criteria are disqualified from enrollment in the study:

1. Inability to give informed consent.
2. Experienced severe sepsis or septic shock within the previous 12 weeks.
3. Last HU dose was ingested within the previous 4 weeks.
4. Currently pregnant or breast-feeding.
5. ALT ≥ 2X the upper limit of normal or albumin <2.0 mg/dL or direct (conjugated) bilirubin ≥ 1.5 mg/dl.*
6. Serum creatinine >2.9 mg/dL and calculated creatinine clearance <30 mL/min.^#^ *
7. Platelet count >800 x 10^9^/L.*
8. Absolute neutrophil count <1.5 x 10^9^/L.*
9. Female of active childbearing potential^$^ who is unwilling to use at least one of the two following forms of birth control: **(i)** not having heterosexual sexual contact beginning at the screening visit and continuing until 4 weeks after the last dose of THU-decitabine OR **(ii)** intrauterine device (IUD).
10. Sexually active male who is unwilling to use a condom when engaging in any sexual contact with a female with child-bearing potential^$^, beginning at the screening visit and continuing until 4 weeks after taking the last dose of THU-decitabine. This requirement applies also to males who have had a successful vasectomy.
11. Altered mental status or recurrent seizures requiring anti-seizure medications.
12. Moribund or any concurrent disease (e.g., hepatic, renal, cardiac, metabolic) of such severity that death within 24 weeks is likely.
13. Concurrent diagnosis of malignancy including MDS, leukemia, or an abnormal karyotype.
14. Vitamin-B12, folate, or iron deficient (until corrected) *.
15. New York Heart Association (NYHA) class III/IV status.
16. Eastern Co-operative Oncology Group (ECOG) performance status ≥3.
17. Participant is on chronic transfusion therapy (e.g., for history of TIA or stroke) and medically contraindicated to discontinue transfusions (unless multiple allo-antibodies prevent the patient from getting transfusions as scheduled).
18. Known history of illicit drug or alcohol abuse within the past 12 months.
19. Other experimental or investigational drug therapy in the past 28 days.

*Based on screening laboratory values.

# Formula for calculating creatinine clearance (Cockcroft-Gault formula): Male GFR = (140 – age) x (weight) / (sCr x 72); Female GFR = (140 – age) x (weight) x 0.85 / (sCr x 72). Annotation – GFR is Glomerular Filtration Rate is ml/min; age is in years; Weight is Lean Body Mass in kilograms; sCr = Serum Creatinine in mg/dl.

^$^ Definition of female of active child bearing potential: all females except those: **(i)** who are post menopausal (appropriate age and no period for more than 12 months) OR **(ii)** have had a hysterectomy and/or bilateral oopherectomy OR **(iii)** have been taking the oral contraceptive pill (OCP) for at least 12 weeks and will continue to do so (in other words, the OCP is a pre-existing component of routine care in the subject) OR **(iv)** have been receiving depo-provera (medroxyprogesterone acetate) contraception for at least 12 weeks (in other words, depo-provera is a pre-existing component of routine care in the subject). Females who are already on the OCP or depo-provera must continue their birth control until 4 weeks after the last dose of THU-decitabine OR **(v)** have had a bilateral tubal ligation (undergoing this procedure will not be suggested to patients being screened for inclusion but will rather be a matter recorded as part of the medical history query).

- 1. Stopping Rules/Subject Discontinuation

- Subjects may decide to discontinue participation at any time during the study.

- Investigators may discontinue any subject at their discretion if, in their professional opinion, the subject’s health, safety, and/or well-being is threatened by continued participation in the study. In the event of this scenario, it may be unsafe to expose additional subjects to the risk of study participation. However, severe adverse events are common in patients with sickle cell disease (constituting part of the rationale for inclusion of placebo in the clinical trial). To provide additional judgment as to whether this scenario constitutes a safety signal warranting termination of the trial, versus an adverse event from underlying SCD, this scenario will result in a hold on study enrollment until review of the data by the Independent Safety Officers. Only after review and written permission from the head of the Independent Safety Officers, will enrollment be allowed to proceed.

- Pregnancy will require discontinuation of affected subjects. Note that good instruction of patients, and all precautions, should be taken to avoid pregnancy while on study.

- Unexpected serious adverse events (SAEs) require discontinuation of affected subjects

- If 3 of the first 5 patients in the study to receive study drug experience ≥grade 3 non-hematologic toxicity attributed to study drug, the study will be terminated.

By using conservative thresholds of the platelet count and neutrophil count that are not associated with clinical toxicity as sentinels for recommending dose modifications (absolute neutrophil count (ANC) <1.5 x 10^9^/L or platelets >1200 x 10^9^/L), the objective is to constrain biological activity of decitabine within set limits to maximize safety. In addition, clinical adverse events (AE), defined as ≥ grade 3 non-hematologic toxicity, trigger dose modification. All AEs will be classified according to the NCI CTCAE v 4.0 and monitored closely throughout the study.

- 1. Replacement of study subjects

- Subjects who discontinue prematurely from the study for any reason will be encouraged to complete, at a minimum, all safety assessments collected at the follow-up visit.

- Subjects who discontinue prior to their Week 4 visit will be replaced, unless the discontinuation is because of side-effects or toxicity that is likely or probably from study drug in the judgment of the treating investigators, in which case the subject will be included in the safety analysis (please also see Stopping Rules Section 5.6).

- Subjects who miss >4 drug administrations because of non-compliance, unless the non-compliance is because of side-effects or toxicity that is likely or probably from study drug in the judgment of the treating investigators, in which case the subject will be included in the safety analysis (please also see Stopping Rules Section 5.6).

- 1. Drug Handling and Administration

**5.8.1 Decitabine**

Packaging and Labeling of Decitabine Study Drug

The decitabine to be used is manufactured by ASH Stevens (Detroit, MI, USA) per Federal Good Manufacturing Practice guidelines. Containers should be tightly closed and stored in the freezer at -20^O^C. After randomization of a patient, 160 mg of decitabine is weighed out into 16 separate glass vials (10 mg of dry powder in each vial for the 16 dosages to be administered to the patient over the 8 week treatment period). After the products are removed from the bulk supply for an individual subject, they are re-packaged into vials; a label, which includes the following:  the (i) name of the drug; (ii) quantity of drug; (iii) date of packaging; (iv) recommended storage temperature; (v) volume of water to be used for reconstitution (vi) lot number of bulk drug used to generate the vials, will be placed on the packet that contains the vials for the particular subject. In addition, the individual vials will have a label with the drug abbreviation (DEC).

Packaging of Decitabine Study Drug

Decitabine is packaged at the University of Illinois at Chicago Hospital Pharmacy.

Reconstitution of Decitabine for Oral Administration

Prior to administration, a glass bottle containing decitabine will be brought to room temperature, and dissolved in 2ml water to create a 5 mg/ml decitabine solution. This decitabine solution can be further diluted into a total volume of 20 ml of water to create a 0.5 mg/ml decitabine solution to facilitate withdrawal of the appropriate volume for administration: a volume of solution containing the appropriate dose will be removed, diluted further with water to a total volume of 30 ml and given by mouth. The time between reconstitution of drug and oral administration should not exceed 60 minutes, and remaining solution is to be discarded. Owing to instability in solution, the decitabine dosing solution should be prepared individually for each patient and “immediately” consumed orally.

Storage and Stability of Reconstituted Decitabine

The glass bottles containing decitabine should be stored at -20^O^C. The drug substance has been tested after 18 months of storage at -20^O^C and no change in appearance, assay or impurities was observed. Decitabine is stable as a dry powder, however, degrades in a pH dependent manner in aqueous solution (optimal pH for stability is 7.0). Decitabine is hydrolyzed in aqueous solution at pH 7.4 at a rate of approximately 2.5% every 7 hours at 4^O^C.

**5.8.2 Tetrahydrouridine (THU)**

Packaging and Labeling of THU Study Drug

The THU to be used is manufactured by ASH Stevens (Detroit, MI, USA) per Federal Good Manufacturing Practice guidelines. Containers should be tightly closed and stored in the freezer at -20^O^C. After randomization of a patient, an amount of tetrahydrouridine equivalent to the amount to be administered during the 8 week treatment period, with a little extra in anticipation of some wastage during transfers between containers, is divided into 16 glass vials/bottles. After the products are removed from the bulk supply for an individual subject, they are re-packaged into vials; a label, which includes the following:  the (i) name of the drug; (ii) quantity of drug; (iii) date of packaging; (iv) recommended storage temperature; (v) volume of water to be used for reconstitution (vi) lot number of bulk drug used to generate the vials, will be placed on the packet that contains the vials for the particular subject. In addition, the individual vials will have a label with the drug abbreviation (THU) and a subject identifier.

Packaging of THU Study Drug

THU is packaged at the University of Illinois at Chicago Hospital Pharmacy.

Reconstitution of THU for Oral Administration

Prior to administration, a glass bottle containing THU will be brought to room temperature, and dissolved in 2 ml water with a further dilution to a final volume of 10 ml, to create a solution with a final concentration that is determined by the amount of drug in the glass vial (THU is soluble to at least 100 mg/ml in water). Using this final concentration, a volume of solution containing the appropriate dose will be removed, diluted further with water to a total volume of 30ml and given by mouth. The time between reconstitution of drug and oral administration should not exceed 60 minutes, and remaining solution is to be discarded.

Storage and Stability of Reconstituted THU

A 1 year stability study on the bulk drug at two temperatures as well as re test data on two lots stored in the freezer for about 4 years have been completed that demonstrate THU stability when stored at -20°C.

**5.8.3 Placebo**

The placebo is plain water which will be dispensed at similar volume and in the same containers as study drug. The water placebo has similar appearance and taste to study drug, since study drug is highly diluted in water.

**5.8.4 Return and Destruction of Study Drug**

Each site will return or destroy any remaining study drug according to their site’s standard operating procedures.

- 1. Timing between oral tetrahydrouridine and oral decitabine administration

Oral tetrahydrouridine at a dose of 10 mg/kg is administered a minimum of 60 ± 15 minutes before administration of oral decitabine. The maximum acceptable interval between oral THU and oral decitabine administration is 2 hours. The timing of THU and decitabine administration will be recorded in the chart and captured in case report forms.

- 1. Timing between drug administrations each week

The planned schedule is for the 2X/week doses to be administered on two consecutive days each week. However, there is a window of ± 2 days for administration of each dose of study drug.

- 1. Randomization procedures

Randomization is done by the University of Illinois at Chicago by Dr. Michael Pacini using a randomization table created on [www.randomization.com](http://www.randomization.com/). Subjects are enrolled to the next available treatment. Subjects, but not investigators, are blinded as to the assigned treatment. The experimental treatment is highly diluted in water. Therefore, the method of subject blinding is administration of water placebo, with similar appearance, volume and taste as study drug.

Study investigators are not blinded to assigned treatment to avoid a mistaken conclusion that drug levels are below the limit of quantification based on analysis of pharmacokinetic data from a patient assigned to placebo.

- 1. Starting dose and dose escalation

The 0.01 mg/kg starting dose of decitabine will be escalated through the following dose levels: 0.02, 0.04, 0.08 to a maximum of 0.16 mg/kg (~5 mg/m^2^) OR until C_max_ 0.2 µM, whichever comes first. This dose escalation is modified as follows if any of the 5 patients in a treatment cohort requires a dose modification: the dose to be used in the next dose level is the cumulative decitabine dose administered over 8 weeks to the three decitabine-treated patients in the previous cohort divided by 48 (to arrive at the dose to be given 2X/wk). There are a maximum of 5 dose levels. If C_max_ is > 0.2 µM with the first dose, the second dose is decreased 10-fold to 0.001 mg/kg and two-fold dose escalation is performed to a maximum of 0.016 mg/kg OR until C_max_ 0.2 µM, whichever comes first.

- 1. Dose modification of oral decitabine within a cohort

Based on our clinical studies with SQ and IV decitabine to treat SCD, an increase in platelets and a decrease in neutrophils are reliable biological indicators of non-cytotoxic DNMT depletion and differentiation modification by decitabine. By using conservative thresholds of the platelet count and neutrophil count that are not associated with clinical toxicity as sentinels for recommending dose modifications (absolute neutrophil count (ANC) <1.5 x 10^9^/L or platelets >1200 x 10^9^/L), the biological activity of decitabine can be constrained within set limits to maximize safety; using this approach, there have been no clinical adverse events attributed to decitabine in the SCD and β-thalassemia studies to date (13 patients with SCD, and 5 patients with β-thalassemia intermedia have been treated with subcutaneous decitabine for between 12-36 weeks in clinical trials).

*Safety of platelet count threshold used to trigger dose modification*: The platelet count threshold selected to trigger dose modification is based on the following clinical data, which suggests that this threshold is well within clinical ranges that are not associated with and increased risk for thrombotic events. Decitabine therapy can alter cellular differentiation. In the hematopoietic compartment, these shifts include increased erythropoiesis and megakaryopoiesis^15;67^. Therefore, increases in platelet counts are expected. Observational studies in thalassemia have examined for an association between platelet counts and thrombotic events: post-splenectomy platelet counts as high as 1,600x10^9^/L were not associated with an increased risk for thrombotic events in two studies^113;114^, but were associated with an increased risk for thrombotic events in another^115^. In this latter study, another identified association was between nucleated RBC numbers and thrombosis^115^. An important question is whether these associations reflect cause-effect relationships. To answer this question, data from patients without hemoglobinopathies may also be informative. In 5766 older individuals followed longitudinally for more than a decade, a higher platelet count was not associated with an increased incidence of thrombo-hemorrhagic vascular events^116^. In the blood cancer essential thrombocytosis, the risk of thrombosis appears to be a function of malignant platelet quality defects rather than the absolute platelet count (reviewed in^117^). In contrast, a number of hemoglobinopathy studies have suggested that RBC pathology is an underlying cause of thrombosis^118-120^. Hence, although it is intuitive to expect an association between platelet count and thrombosis risk, this has not been evident in a number of studies, whereas qualitative RBC defects (or qualitative platelet defects) likely have a role. Notably, in a SCD study using a similar regimen of decitabine, multiple indices of RBC pathology improved accompanied by improvement in multiple markers of coagulation pathway activity, despite platelet count increases to > 800 x 10^9^/L^15^. Thus, it is possible that decitabine induced improvements in RBC characteristics could reduce the risk of thrombosis despite concurrent increases in platelets.

Clinical adverse events (AE), defined as ≥ grade 3 non-hematologic toxicity, also trigger dose modification. All AEs will be classified according to the NCI CTCAE v 4.0 and monitored closely throughout the study.

Patients are monitored weekly. Study drug is held if hematologic thresholds for dose modification are triggered (ANC <1.5 x 10^9^/L, or platelets >1200 x 10^9^/L), or ≥grade 3 non-hematologic toxicity occurs. After recovery below these thresholds, drug is restarted with a 25% decrease in dose. If after decrease in dose, toxicity recurs, repeat this process.

*Hematopoietic side-effects and toxicity*: The anticipated side-effects are thrombocytosis (an increase in the platelet count) concurrent with neutropenia (a decrease in the neutrophil count). **Table 1** describes the dose modifications that should be performed for changes in blood counts.

Table 1: Blood Count Parameters and Laboratory Values Requiring Dose Modification

| **Measurement** | **Trigger Value for Dose Modification** | **Dose Modification*** |
| --- | --- | --- |
| ANC | <1.5x10^9^/L | Withhold until ANC > 1.5x10^9^/L*, then restart with dose decreased in size by 25%. |
| Platelets | >1,200x10^9^/L OR >400x10^9^/L over base-line platelet counts (whichever is higher) | Withhold until platelets <trigger value*, then restart with dose decreased in size by 25%. |
| Hemoglobin | >12g/dl | Withold until hemoglobin <12g/dl*, then restart with dose decreased in size by 25%. |
| Serum Creatinine | ≥1.5 mg/dL (if pre-treatment value <0.5 mg/dL)  ≥1.7 mg/dL (if pre-treatment value 0.6-0.8 mg/dL)  Doubling of creatinine (if pre-treatment value 0.9-2.9 mg/dL) | Withhold until creatinine within 10% of trigger value*, then restart with dose decreased in size by 25%. |
| ALT | ALT≥ 2X base-line value in patient | Withhold until ALT returns to below trigger value* then restart with dose decreased in size by 25%. |

*CBC checks as per study schedule and additional if clinically indicated. The duration of the treatment course or timing of doses will not be altered to compensate for any missed doses.

*Treatment of patients with renal insufficiency or liver function test abnormalities*: Decitabine is rapidly inactivated *in vivo* by ubiquitously expressed CDA and by hydrolysis. Hence, it is acceptable to administer decitabine to subjects with renal and/or liver function test abnormalities, unless these organ dysfunctions are so severe that death is imminent. Renal excretion accounts for ~50% of THU elimination. Therefore, renal insufficiency is anticipated to increase THU half-life. However, THU has a benign toxicity profile at much higher doses than administered in this study. Furthermore, renal and liver function test abnormalities secondary to the underlying disease process are frequent in sickle cell disease, and the overall objective of this clinical trial is to develop a disease modifying intervention for such patients that might consequently improve renal and liver function test abnormalities.

*Gastrointestinal system toxicity:* In addition to monitoring laboratory values, at each clinic visit, the subject will be questioned about the occurrence of nausea, vomiting, diarrhea, stomatitis, neuropathies, fever, and any other unusual complaints. If a complaint cannot be explained by other reasons, study drug will be withheld until the event resolves. If >grade 2 symptoms persist for more than 2 weeks, the subject will be discontinued from the study.

*Nausea/vomiting*: Mild nausea/vomiting have been reported with the relatively high dose decitabine used to treat malignant disease. Antiemetics will be used to control this event, if needed. If antiemetics are unable to control the subject’s nausea/vomiting, study drug will be withheld until the event resolves. Study drug then be resumed with a decrease in dose by 25%. If in the judgment of the treating investigator, the drug is not tolerated, the subject will be discontinued from the study. The duration of the treatment course or timing of doses will not be altered to compensate for any missed doses.

*Diarrhea:* If a subject develops grade 2 or higher diarrhea, study drug will be withheld until resolution of the symptoms. Study drug will then be resumed with a decrease in dose by 25%. If in the judgment of the treating investigator, the drug is not tolerated, the subject will be discontinued from the study. In all instances, the subjects will be encouraged to increase their fluid intake. If the subject shows any signs or symptoms of dehydration, he/she will be admitted for rehydration. The duration of the treatment course or timing of doses will not be altered to compensate for any missed doses.

*Stomatitis:* If a subject develops grade 2 or higher stomatitis, he/she will not receive any study drug until the event has resolved. Study drug will then be resumed with a decrease in dose by 25%. If in the judgment of the investigator, the drug is not tolerated, the subject will be discontinued from the study. Local therapy (i.e., topical therapy applied to the affected site) will be used at the discretion of the subject’s physician. The duration of the treatment course or timing of doses will not be altered to compensate for any missed doses.

*Fever:* All subjects with fevers >38.5˚C will be aggressively investigated for the etiology and treated appropriately. If the subject is experiencing a common, uncomplicated viral syndrome and the ANC is >1.5x10^9^/L, treatment with study drug will continue. However, if the subject develops any other infection, study drug will be withheld until it has been adequately treated, that is, THU-decitabine will be withheld until in the judgment of the treating physician the infection is controlled. Treatment will then resume at the same dose of decitabine unless the ANC was <1.5x10^9^/L in which case the recommendations for ANC <1.5x10^9^/L will be followed. The duration of the treatment course or timing of doses will not be altered to compensate for any missed doses.

*Infection:* If the subject is experiencing a common, uncomplicated viral syndrome and the ANC is >1.5x10^9^/L, treatment with study drug will continue. However, if the subject develops any other infection, study drug will be withheld until it has been adequately treated, that is, decitabine will be withheld until in the judgment of the treating physician the infection is controlled. Treatment will then resume at the same dose of decitabine unless the ANC was <1.5x10^9^/L in which case the recommendations for ANC <1.5x10^9^/L will be followed. The duration of the treatment course or timing of doses will not be altered to compensate for any missed doses.

- 1. Prior and Concomitant Therapy

In general, medications and care consistent with the standard of care for subjects with SCD will be allowed in this study. However, there are specific constraints regarding HU use. With regards to HU there are mechanistic reasons to expect efficacy antagonism and additive toxicity. Therefore, for HU, there is a required wash-out period of 4 weeks prior to enrollment (see **Table 2**).

*Allowed Medications:* Analgesics, as required, for the management of pain crises. Medications consistent with standard of care.

*Prohibited Medications:* HU or any experimental agent other than decitabine.

Table 2 Medications Requiring a Washout Prior to Study Drug Dosing

| **Medication Requiring a Washout Period Prior to Study Drug Dosing** | **Washout Period** |
| --- | --- |
| HU | 28 days |
| Experimental Therapy | 28 days |

- 1. Subject Compliance

Since doses are administered in the clinic, study personnel will ensure that the full, appropriate dose is administered. There is a window of ± 2 days for administration of each dose of study drug. If the visit is outside this window, then the subject will not receive the intended dose for that week. Compliance will be recorded on the CRF and any missed doses will be recorded as protocol violations. The duration of the treatment course or timing of doses will not be altered to compensate for any missed doses. If more than 4 doses are missed from non-compliance, the patient will be taken off study and replaced (if non-compliance or study withdrawal is because of side-effects that in the judgment of the treating physician are likely or probably related to study drug, the subject will not be replaced). There is also a window of ± 2 days for follow-up visits.

- 1. Pre-Treatment Evaluations

Pre-Treatment Evaluations at the Screening Visit, Week –2 or -1

The pre-treatment period is divided into a screening study visit and an enrollment study visit. Prior to the performance of any study-related procedures, subjects must sign the informed consent form at or before the screening study visit.

Week -2 or Week -1, Screening Visit:

- - Complete medical history from the preceding year, major complications of sickle cell disease from birth (for example, stroke, acute chest syndrome etc.), and major non-sickle cell related diagnoses from birth (for example, cancer diagnoses)
  - For females: last menstrual period, history of hysterectomy and/or oopherectomy, history of bilateral tubal ligation, history of oral contraceptive or depo-provera (medroxyprogesterone acetate) use.
  - Crisis frequency and descriptions (over the preceding 1-year)
  - Physical examination, ECOG, and vital signs, including weight
  - Blood and urine will be collected for the following laboratory studies:

- Hemoglobin electrophoresis

- Erythropoietin level

- Urinalysis

- CBC with differential and reticulocyte count

- Complete metabolic panel

- Pregnancy test (serum or urine) in females with child-bearing potential^$^

- LDH

- Coagulation and inflammatory pathway activation measures (D-Dimers and CRP)

- %HbF

Pre-Treatment Evaluations at the Enrollment Visit, Week 1 (just before starting study drug):

- - Blood and urine will be collected for the following laboratory studies:

- CBC with differential and reticulocyte count

- Pregnancy test (serum or urine) in females with child-bearing potential^$^

- LDH

- Coagulation and inflammatory pathway activation measures (D-Dimers and CRP)

- %HbF

- Scientific measurements of DNMT1 and DNA methylation

- Scientific measurement of cytidine deaminase (CDA) genotype and expression

- Scientific measurement of CDA enzyme activity in serum

(some laboratory evaluations are repeated at the screening and enrollment visits to provide a more robust base-line)

- 1. Schedule of On-Study and Follow-up Evaluations and Events

Subjects will start study drug during Week 1. The maximum time interval between screening visit and initiation of study drug (enrollment visit) should be no longer than 2 weeks. Subjects will come to the clinic 2X/wk for study drug administration (drug administration will be scheduled ~24 hours apart, although there is a ±2 days window for drug administration and for study assessments, to accommodate patient practical needs).

Evaluations to be performed after administration of first dose of decitabine:

- - Blood for PK analysis is collected at 0, 2, 4, and 24 hours after administration of the single dose of oral decitabine.

Weeks 1 to 12 (summarized in **Table 3):**

Evaluations to be performed every week (first visit of the week – that is, once a week) prior to administration of decitabine in weeks 1-8, then during follow-up in weeks 9-12:

- - Weight, vital signs, ECOG, interim medical history including determination of birth control measures, and directed physical exam.
  - Adverse events and crisis occurrence and concommitant medications documentation (concomitant medications to include medications started within 30 days of first dose of study drug).
  - Blood will be collected for the following laboratory studies:

- CBC with differential and reticulocyte count

Evaluations to be performed every 2 weeks (first visit of the week, that is, once in that week) prior to administration of study drug in weeks 1-8, then in week 10 and 12:

- - Blood and urine will be collected for the following laboratory studies:

- Complete metabolic panel

- LDH

- Pregnancy test (serum or urine) in females with childbearing potential^$^

- %HbF (except week 6)

Evaluations to be performed every 4 weeks (first visit of the week, that is, once in that week) prior to administration of study drug in weeks 1-8, then in week 12:

- - Blood and urine will be collected for the following laboratory studies:

- D-dimers (also in week 10)

- C-reactive protein (CRP) (also in week 10)

- Buffy coat isolation for DNMT1 and DNA methylation analysis (also in week 10; not performed week 12)

- CDA enzyme activity in serum (also in week 10; not performed week 12)

- Urinalysis
- mRNA extraction (week 1 and week 8 only)

^$^ Definition of female of active child bearing potential: all females except those – (i) who are meno-pausal (appropriate age and no period for more than 12 months) OR (ii) have had a hysterectomy and/or bilateral oopherectomy OR (iii) have been taking the oral contraceptive pill (OCP) for at least 12 weeks and will continue to do so (in other words, the OCP is a pre-existing component of routine care in the subject) OR (iv) have been receiving depo-provera (medroxyprogesterone acetate) contraception for at least 12 weeks (in other words, depo-provera is a pre-existing component of routine care in the subject). Females who are already on the OCP or depo-provera must continue their birth control until 4 weeks after the last dose of THU-decitabine OR **(v)** have had a bilateral tubal ligation (undergoing this procedure will not be suggested to patients being screened for inclusion but will rather be a matter recorded as part of the medical history query).

Table 3: Study Evaluations and Timings

| **Study Evaluations and Timings (timings in week of treatment, first week of treatment is week 1)** | **Pre-Tx*** | **1^#^** | **2** | **3** | **4** | **5** | **6** | **7** | **8** | **9** | **10** | **11** | **12** |
| --- | --- | --- | --- | --- | --- | --- | --- | --- | --- | --- | --- | --- | --- |
| Sign informed consent form | X |  |  |  |  |  |  |  |  |  |  |  |  |
| Complete medical history, physical examination, ECOG and vital signs (including weight) | X |  |  |  |  |  |  |  |  |  |  |  |  |
| Study drug oral administration (2X/wk) |  | X | X | X | X | X | X | X | X |  |  |  |  |
| Measurement of plasma decitabine levels (before, and 2, 4 and 24 hours after administration of the first dose of decitabine) |  | X** |  |  |  |  |  |  |  |  |  |  |  |
| Interim medical history, physical examination, ECOG, weight and vital signs |  | X | X | X | X | X | X | X | X | X | X | X | X |
| Crises and Adverse Event Documentation | X | X | X | X | X | X | X | X | X | X | X | X | X |
| CBC with differential, reticulocytes | X | X | X | X | X | X | X | X | X | X | X | X | X |
| Hb electrophoresis | X |  |  |  |  |  |  |  |  |  |  |  |  |
| Erythropoietin level | X |  |  |  |  |  |  |  |  |  |  |  |  |
| Complete metabolic panel | X |  | X |  | X |  | X |  | X |  | X |  | X |
| LDH | X | X | X |  | X |  | X |  | X |  | X |  | X |
| Pregnancy test (females with childbearing potential) | X | X | X |  | X |  | X |  | X |  | X |  | X |
| %HbF | X | X | X |  | X |  |  |  | X |  | X |  | X |
| D-dimers | X | X |  |  | X |  |  |  | X |  | X |  | X |
| CRP | X | X |  |  | X |  |  |  | X |  | X |  | X |
| Urinalysis | X |  |  |  | X |  |  |  | X |  |  |  | X |
| Concomitant Medications | X | X | X | X | X | X | X | X | X |  |  |  |  |
| Buffy coat DNMT1 and DNA methylation analysis |  | X |  |  | X |  |  |  | X |  | X |  |  |
| Cytidine deaminase (CDA) serum enzyme activity |  | X |  |  | X |  |  |  | X |  | X |  |  |
| *CDA* gene sequencing |  | X |  |  |  |  |  |  |  |  |  |  |  |
| mRNA extraction^ |  | X |  |  |  |  |  |  | X |  |  |  |  |

* screening visit. ^#^ before first dose of decitabine. **24 hour post-dose PK must be drawn before 2^nd^ dose of study drug is administered. ^only at selected sites

- 1. Pharmacokinetic (PK) measurements

PK studies will be performed after the first dose. The primary objective of the PK analysis is to determine peak decitabine level (C_max_), systemic exposure (area under the plasma concentration-versus-time curve, AUC) and other pharmacokinetic parameters (T_max_, T_1/2_, clearance, and volume of distribution, Vd/F) of oral decitabine administered in combination with THU.

Sample collection: Blood samples will be collected in heparin-containing tubes (5 ml blood) before drug administration and at 2, 4 and 24 hours after administration of the single dose of oral decitabine. These timings are based on the concentration-time profile studies in non-human primates, which indicate C_max_ with oral THU-decitabine is likely to occur somewhere between 2-4 hours. Samples will be processed immediately by centrifugation at 3000 × *g* for 5 min in a refrigerated centrifuge. Decitabine is very unstable in plasma due to rapid inactivation by hydrolysis and by cytidine deaminase. To increase stability, tetrahydrouridine (THU), a cytidine deaminase inhibitor, will be added to the plasma supernatant at a final concentration of 100 μM immediately after centrifugation. Plasma (300 μL) will be then aliquoted into two tubes with the remaining plasma in a third tube. The plasma will be immediately frozen at −80 °C until analysis. All samples will be sent to Jiang Wang, PhASR at The Ohio State University for processing (appendix B).

Determination of plasma drug levels: Plasma decitabine concentrations will be analyzed utilizing a high performance liquid chromatograph (Waters LC 2795) interfaced to a tandem mass spectrometer (Micromass Quattro micro API, Waters Corporation). The chromatographic separation will be performed on a 250 mm × 2.0 mm i.d., 4.0 μm, C_18_ column (YMC Co. Ltd., Kyoto, Japan) and a 20 mm × 2.1 mm i.d., 3.5 μm, C_18_ guard column (Waters Xterra RP18, Waters Corporation, Milford, MA, USA) kept at room temperature. The mobile phase used for chromatographic separation will be composed of 2 mM ammonium acetate containing 0.1% formic acid and methanol, and is delivered using a gradient flow. The mass spectrometer is equipped with an electrospray interface, which will be operated in a positive mode, and controlled by the completely validated Masslynx software (Waters Corporation, Milford, MA, USA). Identification will be performed through selective reaction monitoring mode at *m*/*z*+ 229 → 113.0 for decitabine and *m*/*z*+ 242.0 → 126.0 for the internal standard. The analytical method is currently under development and validation according to the ICH Guideline (ICH Harmonized Tripartite Guideline. Validation of Analytical Procedures: Text and Methodology. Q2(R1). Incorporated in November, 2005) in the laboratory of Dr. Lyubimov (Toxicology Research Laboratory, TRL). Method specificity, linearity, range, accuracy, precision and detection limit will be established and validated. In addition, short-term, long-term and freezing/towing stability will be determined. All sample collection, handling and analysis procedures will be performed according to GLP and GCP requirements. All data and the report will be audited by the Quality Assurance Officer at TRL.

PK analysis: PK analysis will be performed using the Winnonlin software (version 5.0.1, Pharsight NC) and will include the determination of maximum plasma concentration (*C*_max_), time to maximum plasma concentration (*T*_max_), area under the plasma concentration-versus-time curve from time zero to last measurable plasma concentration (*AUC*_0-t_). The terminal rate constant (*
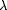
*_z_) will be determined from the slope of the terminal phase of the plasma concentration-time curve using uniform weight. The terminal half-life (T_1/2_) will be calculated as 0.693 divided by *
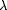
*_z_. Area under the concentration-time curve (AUC) will be calculated using the log/linear trapezoidal rule. The AUC will be extrapolated to infinity (AUC0–
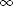
) by using the equation, AUC0–
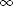
= AUC_0–t_ + C_last_/*
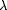
*_z_, where C_last_ will be the final quantifiable concentration.

Plasma levels below the limit of quantification: The anticipated maximum plasma concentrations of decitabine with this study are <<0.2 µM, most likely <0.005 µM, and therefore, there is a very high likelihood that drug levels could be below the lower limit of quantification (LLOQ; the LLOQ by the state of the art LC/MSMS methods to be used in this study is approximately 1 ng/mL (~0.004 µM). If decitabine is not detected by PK analysis in the first patient treated with study drug at a dose level, PK analyses will not be performed in the remaining patients who receive study drug at that dose level (to avoid unnecessary and uninformative patient burden and expense).

- 1. Safety Evaluations

Medical History

At the Pre-Treatment screening visit, the subject’s complete medical history from the past year will be recorded including: previous treatment for SCD and response, previous acute chest syndromes, previous strokes, previous pneumonias, pain crises frequency and usual management, history of avascular necrosis, history of priapism, previous transfusions and reactions, transfusion requirements, complications and therapy of iron overload, previous infections and treatment, allergies, medications, history of previous chemotherapy, radiation, cancer, leukemia or lymphoma therapy, and tobacco, alcohol, and illicit drug use.

The subject’s interim medical history will be recorded every week during treatment, focusing on emergency room (ER) visits, hospitalizations, self-medicated crises, and analgesic usage.

Vital Signs

Vital signs will be recorded at the Pre-Treatment screening visit and every week during treatment. Vital signs measurements include weight, diastolic and systolic blood pressure, heart rate, and temperature.

Physical Examination

A complete physical examination will be performed at the Pre-Treatment screening visit and directed physical examinations are performed every week during treatment. ECOG will be recorded at all study visits.

At the Pre-Treatment screening visit, particular attention will be taken of the neurological, respiratory, cardiac, musculoskeletal, liver and endocrine systems.

At the follow-up clinic study visits, particular attention will be as clinically indicated (directed physical examination).

Pharmacokinetic measurements

Pharmacokinetic measurements are performed to confirm that peak decitabine levels (C_max_) do not exceed 0.2 µM after single dose administration.

Clinical Laboratory Tests

Clinical laboratory tests (including the pregnancy test, if applicable) will be performed on blood samples collected at the Pre-Treatment study visits and every week during treatment. Hematologic parameters will include Hb level, mean cell volume (MCV), reticulocyte counts, white blood cell (WBC) count, platelet count, absolute neutrophil count (ANC) and absolute lymphocyte count. Renal function tests as part of the complete metabolic panel will include potassium (K), sodium (Na), chloride (Cl), bicarbonate (HCO_3_), blood urea nitrogen (BUN), and creatinine. Liver function tests as part of the complete metabolic panel will include AST, ALT, albumin, total protein, total bilirubin, direct bilirubin, indirect bilirubin, and alkaline phosphatase. Urinalysis will be performed on urine samples collected at the Pre-Treatment screening visit and every 4 weeks during treatment.

Post-treatment evaluations

For a more complete safety profile, evaluations as above will continue weekly for 4 weeks after completion of experimental drug therapy.

- 1. Efficacy, Pharmacodynamic (PD), and Scientific Evaluations

SCD-Related Crises

SCD-related crises will be measured at baseline (screening visit) (retrospective estimation for the 1-year period preceding enrollment) and documented every week.

Definitions:

- - *Crisis frequency* is defined as the number of crises divided by the length of time in years over which the number of crises is calculated.
  - *Crisis*, for purposes of analysis, is defined as a pain crisis, pneumonia, acute chest syndrome, priapism, hepatic sequestration, stroke, or initiation of chronic transfusion therapy (as defined below). Pain due to chronic complications of SCD, such as aseptic necrosis, osteomyelitis, or leg ulcers will not be considered as crises.
  - A *pain crisis* is defined as the occurrence of pain in the extremities, back, abdomen, chest, or head that lasts at least 2 hours; requires a visit to a medical facility; and that has no evident cause other than SCD-related VO. Pain due to chronic complications of SCD, such as aseptic necrosis, osteomyelitis, or leg ulcers will not be considered as crises.
  - *Pneumonia* is defined as chest infiltrates on chest X-ray or chest CT scan associated with fever and an identified infectious etiology.
  - *Acute Chest Syndrome* is defined as a new infiltrate on chest X-ray or chest CT scan associated with one or more new symptoms: fever, chest pain, cough, sputum production, dyspnea or hypoxia.
  - *Hepatic sequestration* is defined as a sudden increase in liver size of <2 cm for children and <5 cm for adults associated with pain in the right upper quadrant, a decrease in the Hb concentration of at least 2 g/dL with reticulocytosis, and increased abnormality in liver function tests that cannot be attributed to gall stone disease.
  - *Priapism* is defined as a painful persistent penile erection lasting 30 minutes or more.
- Stroke is defined as a thrombotic or hemorrhagic event involving the central nervous system (as demonstrated by hemorrhage or infarction on CT scan or MRI of the brain or persistently hemorrhagic spinal fluid on lumbar puncture) that results in focal neurologic deficits.
  - *Chronic transfusion therapy* is defined as blood transfusions occurring 1 or more times per month for 3 consecutive months.

Information on the occurrence each of these sickle cell related complications will be collected every study visit and site personnel will be instructed to report each event as an adverse event or serious adverse event as appropriate.

Coagulation and Inflammatory Pathway Activation Measures

Measures of coagulation and inflammatory pathway activation (D-dimers and CRP) will be performed at the screening visit, enrollment visit and at weeks 4, 8, 10 and 12.

Percentage of HbF

The percentage of HbF as a proportion of total hemoglobin (%HbF) will be measured by high performance liquid chromatography. HbF% is measured at the screening and enrollment visits, at weeks 4, 8, 10 and 12.

Global DNA methylation assay

The anticipated effect of this regimen is to produce a plateau (not progressive) level of DNA hypomethylation. This will be confirmed by pyrosequencing of repetitive DNA elements in bisulphite treated DNA extracted from buffy-coat white blood cells in the patient samples. Since cells are not selected for a particular phenotype, a non-specific locus (repetitive DNA elements) is used for assessment of DNA methylation. Bisulfite modification of DNA and pyrosequencing of repetitive element DNA is performed as per standard protocols ^121^ in the laboratory of Dr. Yogen Saunthararajah.

DNMT1 protein level assay

The proximal pharmacodynamic effect of decitabine that precedes DNA hypomethylation is depletion of the DNA methylating enzyme DNMT1. DNMT1 not only methylates DNA but is also an essential component of histone modifying multi-protein complexes. Therefore, the epigenetic consequences of DNMT1 depletion include removal of histone marks (eg., H3K27 trimethylation) associated with transcription repression. Since a spectrum of epigenetic effects are produced downstream of DNMT1 depletion, DNMT1 levels may be a more reliable pharmacodynamic index of the biological consequences of decitabine on transcription regulation than DNA methylation analysis. Therefore, DNMT1 levels (measured by a quantitative immuno-fluorescence assay of peripheral blood nucleated cells) will be correlated with global DNA methylation, HbF levels, and platelet counts.

Cytidine deaminase (*CDA*) genotype at position 79 (evaluation for A79C single nucleotide polymorphism)

Functional studies and prospective clinical data have identified the non-synonymous A79C SNP (RS2072671) in *CDA* as one of particular interest. This SNP produces an A to C transition in the ancestral allele of *CDA* at base position 79, changes lysine to glutamine at amino-acid position 27, and decreases CDA activity by as much as 3-fold ^12,13^, and influences the clinical activity of cytosine analogues. Therefore, CDA genotype could be a pertinent variable that influences safety and efficacy of decitabine therapy ^8-11^.

CDA enzyme activity in the serum

To complement the CDA sequencing analysis, and to understand the time-course effect of THU on CDA enzyme activity, CDA enzyme activity will be measured in the serum using an HPLC based assay. Measurement of CDA expression will complement the enzyme activity assay.

mRNA extraction

mRNA samples will be collected at selected sites to examine the mechanism of action of the study drug and to evalute for factors that my influence response, e.g., CDA and DCK expression levels.

1. Statistical Analysis

***6.1 Sample size.*** The study employs a randomized trial to identify an oral dose of decitabine that can be administered with oral THU over an 8 week period without requiring dose modification. Patients at each dose level of oral decitabine will be monitored weekly with treatment hold for laboratory end-points that should presage any clinical adverse events (in previous clinical trials, as well as in murine toxicology studies, the most sensitive indices of decitabine biologic activity are an increase in the platelet count and a decrease in the absolute neutrophils count). By using conservative thresholds of these indices that are not associated with clinical toxic events as sentinels for holding treatment followed by dose reduction, the biological activity of decitabine can be constrained within set limits of safety; using this approach, there have been no clinically significant toxicity in the SCD or β-thalassemia studies thus far. Therefore, the trigger for dose modification is defined as ANC <1.5 x 10^9^/L or platelets >1200 x 10^9^/L. Clinical adverse events, defined as ≥ grade 3 non-hematologic toxicity also trigger dose modification. The objective of this study is to define the dose of decitabine, that when administered together with oral THU on a 2X/week basis for 8 weeks, has a <30% chance of requiring dose modification.

In each dose level of oral decitabine (starting at 0.01 kg/mg), the objective is to identify a dose of oral decitabine combined with THU that when administered 2X/week for 8 weeks does not require dose modification. Treatment is held and intra-patient dose de-escalation is peformed when dose modification is triggered. If dose modification is required in one or more of the three patients who receive study drug in a dose-level cohort (two patients in each dose level cohort receive placebo), we will accrue another cohort of five new patients (with 3/2 randomization to drug vs. placebo) with the treatment dose based on the cumulative dose administered to patients receiving study drug in the preceding cohort. This process will be repeated until no patient requires dose modification in the three-patient drug treatment cohort.

This is the dose that will be used for any additional patients accumulated to up to a total of 15 patients treated (10 in placebo). If the cumulative rate at which dose modification is required (obtained by time to event analysis) is greater than 30% at any time during the trial, then the Data Toxicity and Safety Committee with input from the PI and key study personnel will determine whether the dose level should be closed. A total sample size of 25 (15 in treatment and 10 in placebo) will have a statistical power of .81 to conclude with at least 90% confidence that there is no difference in ≥ grade 3 non-hematologic toxicity between treatment and placebo groups if the requirement for dose modification in combined treatment groups is less than 30% (assuming no patient with ≥ grade 3 non-hematologic toxicity in placebo group).

***6.2 Statistical Analysis***

Analysis Populations: The Safety Population will include all enrolled subjects who receive at least one dose of decitabine. The Intent-to-Treat (ITT) Population will include all enrolled subjects who receive at least one dose of decitabine and who provide any post-baseline HbF data.

Demographics, Population, and Baseline Characteristics: Subject disposition, demographics, and other relevant baseline information will be summarized for the Safety and ITT Populations.

Sample size and dose escalation: The study employs a phased randomized trial design to identify an oral dose that can be administered over an 8 week period without causing toxicity in patients with SCD. There will be a total of 25 patients with 3/2 randomization to drug vs. placebo.

Safety Data: Incidence of AEs will be tabulated overall and by severity and relationship to oral THU-decitabine. AEs will be coded using the MedDRA dictionary and will be summarized by System Organ Class and MedDRA preferred term. Incidence of AEs will also be tabulated by dose and dose frequency at the time of AE onset. Serious AEs will be described in detail. If the cumulative incidence (obtained by time to event analysis) of combined dose modification rate is greater than 30% at any time during the trial then the Executive Committee consisting of PI and key study personnel will determine whether the dose level should be closed.

Primary End-Point Analysis: Chi-square test will be employed to examine the difference in number of patients with ≥ grade 3 non-hematologic toxicity between treatment and placebo groups. All analyses will be conducted using SAS 9.2 (SAS Inc., Cary, NC) and p-value <0.05 will be considered statistically significant.

Secondary End-Point Analysis - HbF induction: HbF analysis will be performed using the ITT Population. The effect of decitabine on HbF levels over time will be explored using a general linear mixed model with change from baseline as the outcome and with weeks on treatment (as a class variable), baseline HbF level, and a categorical variable indicating dose modification included as fixed effects. The categorical variable indicating dose modification will be defined at the time of each HbF measurement as follows: =1 if the cumulative weekly dose decreased since the previous HbF measurement; =2 if the cumulative weekly dose increased since the previous HbF measurement; and =3 if the cumulative weekly dose did not change since the previous HbF measurement. The general linear mixed model allows exploration of general trends over time while controlling for correlations between repeated measurements made on each subject. Using the MIXED procedure in SAS, 2 models for the covariance structure will be fit, one that assumes a compound symmetric covariance structure (i.e., equivalent correlation between repeated measurements regardless of the timing) and one that assumes an unstructured covariance matrix. If the likelihood ratio test comparing these 2 models is not significant at the 0.05 level, then the simpler compound symmetric structure will be assumed; otherwise, a more complex unstructured covariance matrix will be allowed. Once the final covariance structure is chosen, an appropriate contrast statement will be used to estimate the mean change from baseline to the average HbF level attained from week 8-10 along with a 95% confidence interval.

Additional statistical procedures may be used if necessary (e.g., data transformations). In general, data will be summarized by age group using univariate statistics (e.g., N, mean, standard deviation, median, minimum, and maximum) or frequency (e.g., N, percentage), as appropriate.

Unmasked Interim Analysis and Masked Interim Data Monitoring: No interim analyses will be performed in this study.

1. Data MANAGEMENT
   1. CRF and Source Documentation

The site study coordinator will complete a set of case report forms (CRFs) for each subject. A CRF manual will be provided to each site to assist in correct CRF completion. CRF data must be currently maintained and up-to-date.

Study participants must not be identified by name on any study documents. Subjects will be assigned and identified by subject numbers assigned at enrollment.

- 1. Data Management

The OnCore Database will be utilized, to provide data collection for both accrual entry and trial data management.  OnCore is a Clinical Trials Management System housed on secure servers maintained at Case Western Reserve University.  OnCore properly used is compliant with Title 21 CFR Part 11. Access to data through OnCore is restricted by user accounts and assigned roles. Once logged into the OnCore system with a user ID and password, OnCore defines roles for each user which limits access to appropriate data. User information and password can be obtained by contacting the OnCore Administrator at oncore-registration@case.edu.

OnCore is designed with the capability for study setup, activation, tracking, reporting, data monitoring and review, and eligibility verification. This study will utilize electronic Case Report Form completion in the OnCore database. A calendar of events and required forms are available in OnCore.

- 1. Staff Training

Prior to the onset of enrollment, clinical study coordinators and data coordinators will be centrally trained to ensure adherence to the protocol and assure the highest possible data quality. Training will be led by investigators. Training presentations will address informed consent procedures, study operations and protocol requirements, data collection procedures, maintenance of source documentation, CRF completion and review, routine reporting requirements, data entry and management, and NHLBI policies and procedures.

1. Safety monitoring Plan
   1. Safety Assessments Overview

Subjects enrolled in this trial will receive all therapy and monitoring that is considered standard of care for subjects with SCD, in addition to the study specific monitoring designed to identify changes in laboratory parameters that can trigger dose modifications to decrease the possibility of clinical adverse events. Subjects will be asked about signs and symptoms at each clinical encounter. Study personnel will collect information on ER visits and hospitalizations, clinic visits for pain, sickle cell related complications, and pain medication use. Information on the occurrence of sickle cell related complications will be collected every visit and site personnel will be instructed to report each event as an adverse event or serious adverse event as appropriate. All clinical information will be collected using CRFs.

- 1. Adverse Events Definitions

All adverse events will be classified relative to their seriousness, severity, and relatedness.

The following definitions have been adapted from FDA regulations and ICH guidelines for use in this study:

- An AE is defined as any untoward medical occurrence in a study subject that does not necessarily have a causal relationship with treatment administered in the study. An AE can be any unfavorable and unintended sign (including an abnormal laboratory finding), symptom, or disease temporally associated with the study. Adverse Event data are recorded on the Case Report Form (CRF).
- A *serious* SAE is defined as an AE that results in death, is life-threatening (i.e., an event in which the subject was at risk of death at the time of the event, it does not refer to an event which hypothetically might have caused death if it were more severe), requires inpatient hospitalization or prolongation of existing hospitalization, or results in persistent or significant disability/incapacity; results in a congenital anomaly/birth defect. In the opinion of the investigator, important medical events that may not be immediately life-threatening or result in death or hospitalization, but may jeopardize the subject or may require intervention to prevent one of the other outcomes listed above, may be considered serious. Examples of such events are intensive treatment in an emergency room or at home for allergic bronchospasm, blood dyscrasias or convulsions that do not result in hospitalization, or development of drug dependency or drug abuse.
- An *adverse clinical laboratory trend* is a shift, in an adverse direction, in the mean (or median) change from baseline of a clinical laboratory parameter that is more adverse in an active treatment group than in a control group. For example: mean ALT change from baseline increases significantly more in the active treatment group than in the control group.
- For the purposes of this study, an expected SAE is an AE that is serious, expected, and likely related to the subject’s underlying disease process. For this study, the AEs listed in **table 4** are expected SAEs.
- For the purposes of this study, an unexpected SAE is an AE that is serious, unexpected, and potentially related to the study drug / therapy. For this study, unexpected SAEs are AEs that are not listed in **table 4**. This also includes events that are anticipated within the drug class but not specifically mentioned as occurring with the particular drug under investigation.

Table 4: List of Expected Adverse Events (AEs)

| Acute chest syndrome (ACS) | Elevated urinary urobilinogen | Pain, abdominal |
| --- | --- | --- |
| Anemia | Fatigue | Priapism |
| Aplastic crisis/anemia | Fever | Proteinuria/albuminuria |
| Arthralgia | Hand-foot syndrome | Pulmonary embolus |
|  | Hematuria | Pulmonary hypertension |
| Avascular necrosis of femoral head, hip, or shoulder | Hemiplegia | Pulmonary parenchymal infiltrates  on chest x-ray |
|  | Hemolysis | Pyelonephritis |
| Bone infarction | Hepatic sequestration | Renal insufficiency |
| Cardiomegaly | Hepatosplenomegaly | Renal failure |
| Cerebrovascular accident | Hyperplastic bone marrow | Renal papillary necrosis |
| Cholecystitis | Hyposthenuria | Reticulocytosis (>10%) |
| Cranial nerve palsy | Hypoxemia (pO2 < 65 mmHg) | Retinal hemorrhage or retinal disease |
| Constipation | Infection | Rhabdomyolysis |
| Cough | Jaundice/Liver function test abnormalities | Sepsis |
| Congestive heart failure | Leukocytosis or Leukopenia | Skin ulcers |
| Decreased kidney function | Meningitis | Splenic sequestration |
| Decreased lung function | Nausea | Thrombocytosis |
| Diarrhea | Neutropenia | Thrombosis |
|  | Pain, bone or joint |  |

8.3 Adverse Event Severity and Relatedness to Treatment

Investigators will “grade” the severity of all AEs using the following scale found in CTCAE v 4.0 (**table 5**); the results will be recorded on the CRF and SAE form, if necessary.

Table 5: Severity Grading of AEs

| Severity Grade | Description |
| --- | --- |
| 1 | **Mild**. Awareness of sign, symptom, or event, but easily tolerated; does not interfere with usual daily activities or tasks. |
| 2 | **Moderate**. Discomfort enough to cause interference with usual daily activity; may warrant therapeutic intervention. |
| 3 | **Severe**. Incapacitating; inability to perform usual activities and daily tasks; significantly affects clinical status; requires therapeutic intervention. |
| 4 | **Life-threatening**. Adverse event is life-threatening. |
| 5 | **Death**. Adverse event causes death. |

The nomenclature for assessing the causal relationship between an AE and the study drug is listed in **table 6**. The investigator will determine the category that overall best “fits” the relationship between the AE and the study drug and record the evaluation on the CRF and SAE form, if necessary.

Table 6: Adverse Event Relatedness

| **Relatedness** | **Description** |
| --- | --- |
| Unrelated | - No temporal association to study drug. - An alternate etiology has been established. - The event does not follow the known pattern of response to study drug. - The event does not reappear or worsen with re-challenge. |
| Probably not related / remotely related | - No temporal association to study drug. - Event could readily be produced by clinical state, environmental or other interventions. - The event does not follow the known pattern of response to study drug. - The event does not reappear or worsen with re-challenge. |
| Possibly related | - Reasonable temporal relationship to study drug. - The event is not readily produced by clinical state, environmental, or other interventions. - The event follows a known pattern of response to the study drug or as yet unknown pattern of response. |
| Probably related | - There is a reasonable temporal association with the study drug. - The event is not readily produced by clinical state, environmental, or other interventions. - The event follows a known pattern of response to the study drug. - The event decreases with de-challenge. |
| Definitely related | - There is a reasonable temporal relationship to the study drug. - The event is not readily produced by clinical state, environmental, or other interventions. - The event follows a known pattern of response to the study drug. - The event decreases with de-challenge and recurs with re-challenge. |

8.4 Assessment of Adverse Event Outcome

The clinical investigator will follow every AE to a satisfactory outcome or stabilization of the event, even when this requires a time period beyond the scope of the study. This is particularly applicable to SAEs.

The investigator will record each AE outcome on the CRF. The terms used to describe AE outcome (i.e., outcome of reaction/event at the time of last observation) are as follows:

- Ongoing,
- Resolved without sequelae,
- Resolved with sequelae, or
- Death.

Actions taken in response to an AE and follow-up results (including lab results) will be recorded in the subject’s medical record, in accordance with local procedure. Any treatment administered for the AE must be recorded in the subject’s CRF. When subjects are discontinued from the study due to an AE, relevant clinical assessments and laboratory tests will be repeated as necessary until final resolution or stabilization occurs.

8.5 Serious Adverse Event Reporting; Suspension Guidelines

This study will be reviewed for Data Safety by independent safety officers, Dr. Anjali Advani, MD and Dr. Christy Samaras, DO. Dr. Advani and Dr. Samaras are not associated with this research project and thus work independently of the PI, Dr. Yogen Saunthararajah, MD. They are not part of the key personnel involved in this research study and are qualified to review the patient safety data generated by this study because of their expertise in the area of sickle cell anemia. Serious safety issues that arise in this study will be brought to the attention Drs. Advani and Samaras, and they will make recommendations to the Institutional Review Board (IRB). The IRB will consider the recommendations, determine an appropriate action, and notify the Sponsor. The Sponsor will then notify all participating investigators. This section defines “serious safety issues” and describes procedures for bringing them to the attention of the IRB.

This study is being conducted under an Investigational New Drug (IND) Application. Consequently, U.S. Government regulations require that all “unexpected SAEs” (defined in section 8.2) must be reported to the FDA within either 7 calendar days (fatal or life‑threatening events) or 15 calendar days (non-fatal or non-life-threatening). The FDA has the authority to direct the investigators to suspend or terminate the study, or take other actions.

The PI and study investigators will make the following types of reports that can alert the IRB to a potential safety issue:

- Ad hoc reports of unexpected SAEs that are made within 7 or 15 calendar days, as specified in subsequent paragraphs.

Reporting of Unexpected Serious Adverse Events

SAEs that arise after the Informed Consent is signed until 30 days after the last dose of study drug must be reported. **Table 7** summarizes the procedures and timing for alerting the Independent Safety Officers of unexpected SAEs. Within 24 hours of being informed of the occurrence of an unexpected SAE the investigator must make an initial report to:

- the Sponsor/IND holder
- the institution regulatory specialist

All SAEs must be reported to the sponsor on the Crises and Adverse Event Report Form; this report form should be completed for both initial and follow up SAEs.

The initial SAE report received should include the following minimum information: subject initials, subject study ID,; study drug; an identifiable reporting source; and an event or outcome that can be identified as serious.

The investigator must also report the unexpected SAE to the site’s Institutional Review Board (IRB) in accordance with that IRB’s regulations or procedures.

The investigator, the study coordinator and the institution regulatory specialist will collaborate to prepare a FDA Form 3500A (MEDWATCH) for the unexpected SAE. This report should describe the event as fully as possible.

Supporting documentation (e.g., CRF pages, lab reports, summary notes, autopsy reports) should accompany the report.

A fatal or life-threatening unexpected SAE must be reported to the FDA no later than 7 calendar days after the sponsor’s/ regulatory specialist’s receipt of information. A non-fatal, non-life-threatening unexpected SAE must be reported to the FDA No later than 15 calendar days after the sponsor’s/ regulatory specialist’s receipt of information.

The investigator will follow the progress of a subject who experiences an unexpected SAE until the SAE is resolved or considered stable. When the unexpected SAE has not resolved by the report deadline, the investigator will make follow-up reports in accordance with directions from the Independent Safety Officers, the FDA, and/or the site’s IRB.

Table 7: Summary of Procedures and Timing for Alerting the IRB and NHLBI Project Officer of Unexpected SAEs

| Situation or Event | Summary of Procedure (See text for details.) | Critical Value for IRB “Alert” |
| --- | --- | --- |
| Unexpected SAEs | 1. Investigator notifies Dr Yogen Saunthararajah within 24 hours. 2. Site investigator, Regulatory Specialist, and Dr Yogen Saunthararajah prepare report using FDA forms and submit report to NHLBI Project Officer, IRBs, study investigators. Report: 3. Fatal or life-threatening events: within 7 calendar days. 4. Otherwise: within 15 calendar days. | Alert all cases. |

Reporting of Expected Serious Adverse Events

Investigators will report all expected SAEs within 3 business days to Dr. Yogen Saunthararajah (tel-216 444 8170; email-[saunthy@ccf.org](mailto:saunthy@ccf.org); fax-216 636 2498). The study coordinator and the Institution Regulatory Specialist will collaborate to prepare a report of the expected SAEs that meet the following criteria (applicable FDA reporting format will be used):.

Events occurring at a higher rate than anticipated that meet the following

- Serious
- Expected (known consequences of the disease under investigation, common known events in the study population, or listed in the protocol / investigator brochure)
- Suspected to be related to the investigational product

Site PIs are responsible for reporting all SAEs to the appropriate IRB or ethics committee in accordance with local laws and regulations.

8.6 Subject Discontinuation Due to Adverse Event(s)

In general, a subject who experiences AEs that can reasonably be tolerated, all factors considered, should be encouraged to remain in the study. The investigator and subject must jointly interpret “can reasonably be tolerated, all factors considered.” One point to consider in making the interpretation is that almost all subjects in this study will experience SCD-related AEs. In most cases, when a subject experiences SCD-related AEs that do not stem from the study intervention or treatment, withdrawal from the study would not resolve the AEs.

8.7 Pregnancy Reporting

Decitabine should not be administered to pregnant women and pregnant subjects will be excluded from the study. Female subjects who are pubertal will be regularly assessed throughout the study. In the event of a test indicating the study subject is pregnant, the subject will be informed of this result and will be immediately discontinued from the study.

8.8 Protocol Violations

The investigator will report each protocol violation or deviation to the pertinent IRB. The form provides space for a description of the event, actions already taken to ameliorate the effects of the violation/deviation, and planned additional corrective actions.

1. Subject Compensation

For the screening visit and for all subsequent scheduled study visits (including visits for drug administration), subjects will be reimbursed $40/visit to defray the costs of transportation including parking fees, gas mileage, bus or train fare. Instead of $40, on the first day of treatment, because of the multi-hour interval pharmacokinetic measurements, patients will be reimbursed $100 for extended parking, meal costs etc. Hence, the anticipated total disbursement per patient is $900 (day 1 -$100, day 2 - $40, week 2-8 $80 per week, week 9-12 $40 per week).

1. Protocol Signature Page

I, _____________________________________________, MD agree to conduct:

“Phase 1 Study of Oral Decitabine and Tetrahydrouridine in Patients with High Risk Sickle Cell Disease”

I understand that no deviations from this protocol, version 10.0 dated March 20, 2015, may be made without the written permission of the Sponsor-Investigator, Yogen Saunthararajah, MD, except where necessary to eliminate immediate hazard(s) to trial subjects, or when the change(s) involve only logistical or administrative aspects of the trial.

Signature: _________________________________________

Date: _____________________________________________

REFERENCES

1. Platt OS, Brambilla DJ, Rosse WF et al. Mortality in sickle cell disease. Life expectancy and risk factors for early death. *N.Engl.J.Med.* 1994;330(23):1639-1644.

2. Charache S, Terrin ML, Moore RD et al. Effect of hydroxyurea on the frequency of painful crises in sickle cell anemia. Investigators of the Multicenter Study of Hydroxyurea in Sickle Cell Anemia. *N.Engl.J.Med.* 1995;332(20):1317-1322.

3. Goldberg MA, Husson MA, Bunn HF. Participation of hemoglobins A and F in polymerization of sickle hemoglobin. *J.Biol.Chem.* 1977;252(10):3414-3421.

4. Nagel RL, Bookchin RM, Johnson J et al. Structural bases of the inhibitory effects of hemoglobin F and hemoglobin A2 on the polymerization of hemoglobin S. *Proc.Natl.Acad.Sci.U.S.A* 1979;76(2):670-672.

5. Steinberg MH, Lu ZH, Barton FB et al. Fetal hemoglobin in sickle cell anemia: determinants of response to hydroxyurea. Multicenter Study of Hydroxyurea. *Blood* 1997;89(3):1078-1088.

6. Steinberg MH, Mitchell TE. Novel approaches to treatment of sickle cell anaemia. *Expert.Opin.Investig.Drugs* 1999;8(11):1823-1836.

7. Atweh GF, Schechter AN. Pharmacologic induction of fetal hemoglobin: raising the therapeutic bar in sickle cell disease. *Curr.Opin.Hematol.* 2001;8(2):123-130.

8. Platt OS, Thorington BD, Brambilla DJ et al. Pain in sickle cell disease. Rates and risk factors. *N.Engl.J.Med.* 1991;325(1):11-16.

9. West MS, Wethers D, Smith J, Steinberg M. Laboratory profile of sickle cell disease: a cross-sectional analysis. The Cooperative Study of Sickle Cell Disease. *J.Clin.Epidemiol.* 1992;45(8):893-909.

10. Jones KM, Niaz MS, Brooks CM et al. Adverse effects of a clinically relevant dose of hydroxyurea used for the treatment of sickle cell disease on male fertility endpoints. *Int.J.Environ.Res.Public Health* 2009;6(3):1124-1144.

11. Lukusa AK, Vermylen C, Vanabelle B et al. Bone marrow transplantation or hydroxyurea for sickle cell anemia: long-term effects on semen variables and hormone profiles. *Pediatr.Hematol.Oncol.* 2009;26(4):186-194.

12. NTP-CERHR Monograph on the Potential Human Reproductive and Developmental Effects of Hydroxyurea. *NTP.CERHR.MON.* 2008;(21):i-III1.

13. van der Ploeg LH, Flavell RA. DNA methylation in the human gamma delta beta-globin locus in erythroid and nonerythroid tissues. *Cell* 1980;19(4):947-958.

14. Mavilio F, Giampaolo A, Care A et al. Molecular mechanisms of human hemoglobin switching: selective undermethylation and expression of globin genes in embryonic, fetal, and adult erythroblasts. *Proc.Natl.Acad.Sci.U.S.A* 1983;80(22):6907-6911.

15. Saunthararajah Y, Hillery CA, Lavelle D et al. Effects of 5-aza-2'-deoxycytidine on fetal hemoglobin levels, red cell adhesion, and hematopoietic differentiation in patients with sickle cell disease. *Blood* 2003;102(12):3865-3870.

16. Saunthararajah Y, Molokie R, Saraf S et al. Clinical effectiveness of decitabine in severe sickle cell disease. *Br.J.Haematol.* 2008;141(1):126-129.

17. Covey JM, D'Incalci M, Tilchen EJ, Zaharko DS, Kohn KW. Differences in DNA damage produced by incorporation of 5-aza-2'-deoxycytidine or 5,6-dihydro-5-azacytidine into DNA of mammalian cells. *Cancer Res.* 1986;46(11):5511-5517.

18. Schermelleh L, Haemmer A, Spada F et al. Dynamics of Dnmt1 interaction with the replication machinery and its role in postreplicative maintenance of DNA methylation. *Nucleic Acids Res.* 2007;35(13):4301-4312.

19. Momparler RL, Goodman J. In vitro cytotoxic and biochemical effects of 5-aza-2'-deoxycytidine. *Cancer Res.* 1977;37(6):1636-1639.

20. Jones PA, Taylor SM. Cellular differentiation, cytidine analogs and DNA methylation. *Cell* 1980;20(1):85-93.

21. Halaban R, Krauthammer M, Pelizzola M et al. Integrative analysis of epigenetic modulation in melanoma cell response to decitabine: clinical implications. *PLoS.ONE.* 2009;4(2):e4563.

22. Patel K, Dickson J, Din S et al. Targeting of 5-aza-2'-deoxycytidine residues by chromatin-associated DNMT1 induces proteasomal degradation of the free enzyme. *Nucleic Acids Res.* 2010;38(13):4313-4324.

23. Camiener GW, Smith CG. Studies of the enzymatic deamination of cytosine arabinoside. I. Enzyme distribution and species specificity. *Biochem.Pharmacol.* 1965;14(10):1405-1416.

24. Neil GL, Moxley TE, Kuentzel SL, Manak RC, Hanka LJ. Enhancement by tetrahydrouridine (NSC-112907) of the oral activity of 5-azacytidine (NSC-102816) in L1210 leukemic mice. *Cancer Chemother.Rep.* 1975;59(3):459-465.

25. Desimone J, Heller P, Molokie RE, Hall L, Zwiers D. Tetrahydrouridine, cytidine analogues, and hemoglobin F. *Am.J.Hematol.* 1985;18(3):283-288.

26. Beumer JH, Eiseman JL, Parise RA et al. Modulation of gemcitabine (2',2'-difluoro-2'-deoxycytidine) pharmacokinetics, metabolism, and bioavailability in mice by 3,4,5,6-tetrahydrouridine. *Clin.Cancer Res.* 2008;14(11):3529-3535.

27. Liu Z, Marcucci G, Byrd JC et al. Characterization of decomposition products and preclinical and low dose clinical pharmacokinetics of decitabine (5-aza-2'-deoxycytidine) by a new liquid chromatography/tandem mass spectrometry quantification method. *Rapid Commun.Mass Spectrom.* 2006;20(7):1117-1126.

28. Liu Z, Liu S, Xie Z et al. Characterization of in vitro and in vivo hypomethylating effects of decitabine in acute myeloid leukemia by a rapid, specific and sensitive LC-MS/MS method. *Nucleic Acids Res.* 2007;35(5):e31.

29. Kirch HC, Schroder J, Hoppe H et al. Recombinant gene products of two natural variants of the human cytidine deaminase gene confer different deamination rates of cytarabine in vitro. *Exp.Hematol.* 1998;26(5):421-425.

30. Yue L, Saikawa Y, Ota K et al. A functional single-nucleotide polymorphism in the human cytidine deaminase gene contributing to ara-C sensitivity. *Pharmacogenetics* 2003;13(1):29-38.

31. Gilbert JA, Salavaggione OE, Ji Y et al. Gemcitabine pharmacogenomics: cytidine deaminase and deoxycytidylate deaminase gene resequencing and functional genomics. *Clin.Cancer Res.* 2006;12(6):1794-1803.

32. Fitzgerald SM, Goyal RK, Osborne WR et al. Identification of functional single nucleotide polymorphism haplotypes in the cytidine deaminase promoter. *Hum.Genet.* 2006;119(3):276-283.

33. Garcia-Manero G, Stoltz ML, Ward MR, Kantarjian H, Sharma S. A pilot pharmacokinetic study of oral azacitidine. *Leukemia* 2008;22(9):1680-1684.

34. Sugiyama E, Kaniwa N, Kim SR et al. Pharmacokinetics of gemcitabine in Japanese cancer patients: the impact of a cytidine deaminase polymorphism. *J.Clin.Oncol.* 2007;25(1):32-42.

35. Kreis W, Chan K, Budman DR et al. Effect of tetrahydrouridine on the clinical pharmacology of 1-beta-D-arabinofuranosylcytosine when both drugs are coinfused over three hours. *Cancer Res.* 1988;48(5):1337-1342.

36. Bhatla D, Gerbing RB, Alonzo TA et al. Cytidine deaminase genotype and toxicity of cytosine arabinoside therapy in children with acute myeloid leukemia. *Br.J.Haematol.* 2009;144(3):388-394.

37. Riccardi R, Chabner B, Glaubiger DL, Wood J, Poplack DG. Influence of tetrahydrouridine on the pharmacokinetics of intrathecally administered 1-beta-D-arabinofuranosylcytosine. *Cancer Res.* 1982;42(5):1736-1739.

38. Kreis W, Budman DR, Chan K et al. Therapy of refractory/relapsed acute leukemia with cytosine arabinoside plus tetrahydrouridine (an inhibitor of cytidine deaminase)--a pilot study. *Leukemia* 1991;5(11):991-998.

39. Dover GJ, Charache S, Boyer SH, Vogelsang G, Moyer M. 5-Azacytidine increases HbF production and reduces anemia in sickle cell disease: dose-response analysis of subcutaneous and oral dosage regimens. *Blood* 1985;66(3):527-532.

40. Wong PP, Currie VE, Mackey RW et al. Phase I evaluation of tetrahydrouridine combined with cytosine arabinoside. *Cancer Treat.Rep.* 1979;63(8):1245-1249.

41. Ho DH, Bodey GP, Hall SW et al. Clinica, pharmacology of tetrahydrouridine. *J.Clin.Pharmacol.* 1978;18(5-6):259-265.

42. Kreis W, Woodcock TM, Gordon CS, Krakoff IH. Tetrahydrouridine: Physiologic disposition and effect upon deamination of cytosine arabinoside in man. *Cancer Treat.Rep.* 1977;61(7):1347-1353.

43. Beumer JH, Parise RA, Newman EM et al. Concentrations of the DNA methyltransferase inhibitor 5-fluoro-2'-deoxycytidine (FdCyd) and its cytotoxic metabolites in plasma of patients treated with FdCyd and tetrahydrouridine (THU). *Cancer Chemother.Pharmacol.* 2008;62(2):363-368.

44. Marsh JH, Kreis W, Barile B et al. Therapy of refractory/relapsed acute myeloid leukemia and blast crisis of chronic myeloid leukemia with the combination of cytosine arabinoside, tetrahydrouridine, and carboplatin. *Cancer Chemother.Pharmacol.* 1993;31(6):481-484.

45. Goldenthal EI, Cookson KM, Geil RG, Wazeter FX. Preclinical toxicologic evaluation of tetrahydrouridine (NSC-112907) in beagle dogs and rhesus monkeys. *Cancer Chemother.Rep.3* 1974;5(1):15-16.

46. Santi DV, Garrett CE, Barr PJ. On the mechanism of inhibition of DNA-cytosine methyltransferases by cytosine analogs. *Cell* 1983;33(1):9-10.

47. Santi DV, Norment A, Garrett CE. Covalent bond formation between a DNA-cytosine methyltransferase and DNA containing 5-azacytosine. *Proc.Natl.Acad.Sci.U.S.A* 1984;81(22):6993-6997.

48. Creusot F, Acs G, Christman JK. Inhibition of DNA methyltransferase and induction of Friend erythroleukemia cell differentiation by 5-azacytidine and 5-aza-2'-deoxycytidine. *J.Biol.Chem.* 1982;257(4):2041-2048.

49. Koshy M, Dorn L, Bressler L et al. 2-deoxy 5-azacytidine and fetal hemoglobin induction in sickle cell anemia. *Blood* 2000;96(7):2379-2384.

50. Akpan I, Banzon V, Ibanez V et al. Decitabine increases fetal hemoglobin in Papio anubis by increasing gamma-globin gene transcription. *Exp.Hematol.* 2010;38(11):989-993.

51. Chin J, Singh M, Banzon V et al. Transcriptional activation of the gamma-globin gene in baboons treated with decitabine and in cultured erythroid progenitor cells involves different mechanisms. *Exp.Hematol.* 2009;37(10):1131-1142.

52. Lavelle D, Vaitkus K, Hankewych M, Singh M, Desimone J. Effect of 5-aza-2'-deoxycytidine (Dacogen) on covalent histone modifications of chromatin associated with the epsilon-, gamma-, and beta-globin promoters in Papio anubis. *Exp.Hematol.* 2006;34(3):339-347.

53. Lavelle D, DeSimone J, Heller P. Fetal hemoglobin reactivation in baboon and man: a short perspective. *Am.J.Hematol.* 1993;42(1):91-95.

54. Steinberg MH, Barton F, Castro O et al. Effect of hydroxyurea on mortality and morbidity in adult sickle cell anemia: risks and benefits up to 9 years of treatment. *JAMA* 2003;289(13):1645-1651.

55. Watson J. The significance of the paucity of sickle cells in newborn Negro infants. *Am.J.Med.Sci.* 1948;215(4):419-423.

56. CONLEY CL, Weatherall DJ, RICHARDSON SN, SHEPARD MK, Charache S. Hereditary persistence of fetal hemoglobin: a study of 79 affected persons in 15 Negro families in Baltimore. *Blood* 1963;21:261-281.

57. Saunthararajah Y, Nakamura R, Nam JM et al. HLA-DR15 (DR2) is overrepresented in myelodysplastic syndrome and aplastic anemia and predicts a response to immunosuppression in myelodysplastic syndrome. *Blood* 2002;100(5):1570-1574.

58. Perrine RP, Pembrey ME, John P, Perrine S, Shoup F. Natural history of sickle cell anemia in Saudi Arabs. A study of 270 subjects. *Ann.Intern.Med.* 1978;88(1):1-6.

59. Rosse WF, Narla M, Petz LD, Steinberg MH. New Views of Sickle Cell Disease Pathophysiology and Treatment. *Hematology.(Am.Soc.Hematol.Educ.Program.)* 2000;2-17.

60. Goren A, Simchen G, Fibach E et al. Fine tuning of globin gene expression by DNA methylation. *PLoS.One.* 2006;1:e46.

61. Lathrop MJ, Hsu M, Richardson CA et al. Developmentally regulated extended domains of DNA hypomethylation encompass highly transcribed genes of the human beta-globin locus. *Exp.Hematol.* 2009;37(7):807-813.

62. Ley TJ, DeSimone J, Noguchi CT et al. 5-Azacytidine increases gamma-globin synthesis and reduces the proportion of dense cells in patients with sickle cell anemia. *Blood* 1983;62(2):370-380.

63. Lowrey CH, Nienhuis AW. Brief report: treatment with azacitidine of patients with end-stage beta-thalassemia. *N.Engl.J.Med.* 1993;329(12):845-848.

64. Carr BI, Reilly JG, Smith SS, Winberg C, Riggs A. The tumorigenicity of 5-azacytidine in the male Fischer rat. *Carcinogenesis* 1984;5(12):1583-1590.

65. Yang YM, Pace B, Kitchens D et al. BFU-E colony growth in response to hydroxyurea: correlation between in vitro and in vivo fetal hemoglobin induction. *Am.J.Hematol.* 1997;56(4):252-258.

66. Atweh GF, Schechter AN. Pharmacologic induction of fetal hemoglobin: raising the therapeutic bar in sickle cell disease. *Curr.Opin.Hematol.* 2001;8(2):123-130.

67. Milhem M, Mahmud N, Lavelle D et al. Modification of hematopoietic stem cell fate by 5aza 2'deoxycytidine and trichostatin A. *Blood* 2004;103(11):4102-4110.

68. Hu Z, Negrotto S, Gu X et al. Decitabine maintains hematopoietic precursor self-renewal by preventing repression of stem cell genes by a differentiation-inducing stimulus. *Mol.Cancer Ther.* 2010;9(6):1536-1543.

69. de Haan G, Van Zant G. Intrinsic and extrinsic control of hemopoietic stem cell numbers: mapping of a stem cell gene. *J.Exp.Med.* 1997;186(4):529-536.

70. Koshy M, Dorn L, Bressler L et al. 2-deoxy 5-azacytidine and fetal hemoglobin induction in sickle cell anemia. *Blood* 2000;96(7):2379-2384.

71. DeSimone J, Koshy M, Dorn L et al. Maintenance of elevated fetal hemoglobin levels by decitabine during dose interval treatment of sickle cell anemia. *Blood* 2002;99(11):3905-3908.

72. Saunthararajah Y, Hillery CA, Lavelle D et al. Effects of 5-aza-2'-deoxycytidine on fetal hemoglobin levels, red cell adhesion, and hematopoietic differentiation in patients with sickle cell disease. *Blood* 2003;102(12):3865-3870.

73. Milhem M, Mahmud N, Lavelle D et al. Modification of Hematopoietic Stem Cell Fate By 5aza 2'deoxycytidine and Trichostatin A. *Blood* 2004.

74. Landolph JR, Jones PA. Mutagenicity of 5-azacytidine and related nucleosides in C3H/10T 1/2 clone 8 and V79 cells. *Cancer Res.* 1982;42(3):817-823.

75. McGregor DB, Brown AG, Cattanach P et al. TFT and 6TG resistance of mouse lymphoma cells to analogs of azacytidine. *Carcinogenesis* 1989;10(11):2003-2008.

76. Jackson-Grusby L, Laird PW, Magge SN, Moeller BJ, Jaenisch R. Mutagenicity of 5-aza-2'-deoxycytidine is mediated by the mammalian DNA methyltransferase. *Proc.Natl.Acad.Sci.U.S.A* 1997;94(9):4681-4685.

77. Juttermann R, Li E, Jaenisch R. Toxicity of 5-aza-2'-deoxycytidine to mammalian cells is mediated primarily by covalent trapping of DNA methyltransferase rather than DNA demethylation. *Proc.Natl.Acad.Sci.U.S.A* 1994;91(25):11797-11801.

78. Marquardt H, Marquardt H. Induction of malignant transformation and mutagenesis in cell cultures by cancer chemotherapeutic agents. *Cancer* 1977;40(4 Suppl):1930-1934.

79. Ravetto PF, Agarwal R, Chiswick ML et al. Absence of leukaemic fusion gene transcripts in preterm infants exposed to diagnostic x rays. *Arch.Dis.Child Fetal Neonatal Ed* 2003;88(3):F237-F244.

80. Gaudet F, Hodgson JG, Eden A et al. Induction of tumors in mice by genomic hypomethylation. *Science* 2003;300(5618):489-492.

81. Yang AS, Estecio MR, Garcia-Manero G, Kantarjian HM, Issa JP. Comment on "Chromosomal instability and tumors promoted by DNA hypomethylation" and "Induction of tumors in nice by genomic hypomethylation". *Science* 2003;302(5648):1153.

82. Lubbert M, Wijermans P, Kunzmann R et al. Cytogenetic responses in high-risk myelodysplastic syndrome following low-dose treatment with the DNA methylation inhibitor 5-aza-2'-deoxycytidine. *Br.J.Haematol.* 2001;114(2):349-357.

83. Karpf AR, Lasek AW, Ririe TO et al. Limited gene activation in tumor and normal epithelial cells treated with the DNA methyltransferase inhibitor 5-aza-2'-deoxycytidine. *Mol.Pharmacol.* 2004;65(1):18-27.

84. Leone G, Voso MT, Teofili L, Lubbert M. Inhibitors of DNA methylation in the treatment of hematological malignancies and MDS. *Clin.Immunol.* 2003;109(1):89-102.

85. Glaser KB, Staver MJ, Waring JF et al. Gene expression profiling of multiple histone deacetylase (HDAC) inhibitors: defining a common gene set produced by HDAC inhibition in T24 and MDA carcinoma cell lines. *Mol.Cancer Ther.* 2003;2(2):151-163.

86. Chambers AE, Banerjee S, Chaplin T et al. Histone acetylation-mediated regulation of genes in leukaemic cells. *Eur.J.Cancer* 2003;39(8):1165-1175.

87. Van Lint C, Emiliani S, Verdin E. The expression of a small fraction of cellular genes is changed in response to histone hyperacetylation. *Gene Expr.* 1996;5(4-5):245-253.

88. Baylin SB, Herman JG. DNA hypermethylation in tumorigenesis: epigenetics joins genetics. *Trends Genet.* 2000;16(4):168-174.

89. Jiang Y, Dunbar A, Gondek LP et al. Aberrant DNA methylation is a dominant mechanism in MDS progression to AML. *Blood* 2008.

90. Laird PW, Jackson-Grusby L, Fazeli A et al. Suppression of intestinal neoplasia by DNA hypomethylation. *Cell* 1995;81(2):197-205.

91. Lantry LE, Zhang Z, Crist KA et al. 5-Aza-2'-deoxycytidine is chemopreventive in a 4-(methyl-nitrosamino)-1-(3-pyridyl)-1-butanone-induced primary mouse lung tumor model. *Carcinogenesis* 1999;20(2):343-346.

92. Carr BI, Rahbar S, Asmeron Y, Riggs A, Winberg CD. Carcinogenicity and haemoglobin synthesis induction by cytidine analogues. *Br.J.Cancer* 1988;57(4):395-402.

93. McGregor F, Muntoni A, Fleming J et al. Molecular changes associated with oral dysplasia progression and acquisition of immortality: potential for its reversal by 5-azacytidine. *Cancer Res.* 2002;62(16):4757-4766.

94. Belinsky SA, Klinge DM, Stidley CA et al. Inhibition of DNA methylation and histone deacetylation prevents murine lung cancer. *Cancer Res.* 2003;63(21):7089-7093.

95. Thomas GA, Williams ED. Production of thyroid tumours in mice by demethylating agents. *Carcinogenesis* 1992;13(6):1039-1042.

96. Branch S, Francis BM, Brownie CF, Chernoff N. Teratogenic effects of the demethylating agent 5-aza-2'-deoxycytidine in the Swiss Webster mouse. *Toxicology* 1996;112(1):37-43.

97. Branch S, Chernoff N, Brownie C, Francis BM. 5-AZA-2'-deoxycytidine-induced dysmorphogenesis in the rat. *Teratog.Carcinog.Mutagen.* 1999;19(5):329-338.

98. Cisneros FJ, Branch S. 5-AZA-2'-deoxycytidine (5-AZA-CdR): a demethylating agent affecting development and reproductive capacity. *J.Appl.Toxicol.* 2003;23(2):115-120.

99. Kelly TL, Li E, Trasler JM. 5-aza-2'-deoxycytidine induces alterations in murine spermatogenesis and pregnancy outcome. *J.Androl* 2003;24(6):822-830.

100. Olivieri NF, Saunthararajah Y, Thayalasuthan V et al. A pilot study of subcutaneous decitabine in {beta}-thalassemia intermedia. *Blood* 2011.

101. Rogstad DK, Herring JL, Theruvathu JA et al. Chemical decomposition of 5-aza-2'-deoxycytidine (Decitabine): kinetic analyses and identification of products by NMR, HPLC, and mass spectrometry. *Chem.Res.Toxicol.* 2009;22(6):1194-1204.

102. Hollenbach PW, Nguyen AN, Brady H et al. A comparison of azacitidine and decitabine activities in acute myeloid leukemia cell lines. *PLoS.One.* 2010;5(2):e9001.

103. Guo Y, Engelhardt M, Wider D, Abdelkarim M, Lubbert M. Effects of 5-aza-2'-deoxycytidine on proliferation, differentiation and p15/INK4b regulation of human hematopoietic progenitor cells. *Leukemia* 2006;20(1):115-121.

104. Momparler RL, Frith CH. Toxicology in mice of the antileukemic agent 5-aza-2'-deoxycytidine. *Drug Chem.Toxicol.* 1981;4(4):373-381.

105. Liu Z, Marcucci G, Byrd JC et al. Characterization of decomposition products and preclinical and low dose clinical pharmacokinetics of decitabine (5-aza-2'-deoxycytidine) by a new liquid chromatography/tandem mass spectrometry quantification method. *Rapid Commun.Mass Spectrom.* 2006;20(7):1117-1126.

106. Dareer SM, Mulligan LT, Jr., White V et al. Distribution of [3H]cytosine arabinoside and its products in mice, dogs, and monkeys and effect of tetrahydrouridine. *Cancer Treat.Rep.* 1977;61(3):395-407.

107. Holleran JL, Parise RA, Joseph E et al. Plasma pharmacokinetics, oral bioavailability, and interspecies scaling of the DNA methyltransferase inhibitor, zebularine. *Clin.Cancer Res.* 2005;11(10):3862-3868.

108. Blum W, Klisovic RB, Hackanson B et al. Phase I study of decitabine alone or in combination with valproic acid in acute myeloid leukemia. *J.Clin.Oncol.* 2007;25(25):3884-3891.

109. Appleton K, Mackay HJ, Judson I et al. Phase I and pharmacodynamic trial of the DNA methyltransferase inhibitor decitabine and carboplatin in solid tumors. *J.Clin.Oncol.* 2007;25(29):4603-4609.

110. Atweh GF, Schechter AN. Pharmacologic induction of fetal hemoglobin: raising the therapeutic bar in sickle cell disease. *Curr.Opin.Hematol.* 2001;8(2):123-130.

111. Chabner BA, Johns DG, Coleman CN, Drake JC, Evans WH. Purification and properties of cytidine deaminase from normal and leukemic granulocytes. *J.Clin.Invest* 1974;53(3):922-931.

112. Wentworth DF, Wolfenden R. On the interaction of 3,4,5,6-tetrahydrouridine with human liver cytidine deaminase. *Biochemistry* 1975;14(23):5099-5105.

113. Chuncharunee S, Archararit N, Ungkanont A et al. Etiology and incidence of thrombotic and hemorrhagic disorders in Thai patients with extreme thrombocytosis. *J.Med.Assoc.Thai.* 2000;83 Suppl 1:S95-100.

114. Hathirat P, Mahaphan W, Chuansumrit A et al. Platelet counts in thalassemic children before and after splenectomy. *Southeast Asian J.Trop.Med.Public Health* 1993;24 Suppl 1:213-215.

115. Taher AT, Musallam KM, Karimi M et al. Splenectomy and thrombosis: the case of thalassemia intermedia. *J.Thromb.Haemost.* 2010;8(10):2152-2158.

116. van der Bom JG, Heckbert SR, Lumley T et al. Platelet count and the risk for thrombosis and death in the elderly. *J.Thromb.Haemost.* 2009;7(3):399-405.

117. Tefferi A. Platelet count in essential thrombocythemia: the more the better? *Blood* 2008;112(8):3526-3527.

118. Habib A, Kunzelmann C, Shamseddeen W et al. Elevated levels of circulating procoagulant microparticles in patients with beta-thalassemia intermedia. *Haematologica* 2008;93(6):941-942.

119. Gladwin MT, Kato GJ. Hemolysis-associated hypercoagulability in sickle cell disease: the plot (and blood) thickens! *Haematologica* 2008;93(1):1-3.

120. Ataga KI, Moore CG, Hillery CA et al. Coagulation activation and inflammation in sickle cell disease-associated pulmonary hypertension. *Haematologica* 2008;93(1):20-26.

121. Yang AS, Estecio MR, Doshi K et al. A simple method for estimating global DNA methylation using bisulfite PCR of repetitive DNA elements. *Nucleic Acids Res.* 2004;32(3):e38.

122. Ley TJ, DeSimone J, Anagnou NP et al. 5-azacytidine selectively increases gamma-globin synthesis in a patient with beta+ thalassemia. *N.Engl.J.Med.* 1982;307(24):1469-1475.

123. Charache S, Dover G, Smith K et al. Treatment of sickle cell anemia with 5-azacytidine results in increased fetal hemoglobin production and is associated with nonrandom hypomethylation of DNA around the gamma-delta-beta-globin gene complex. *Proc.Natl.Acad.Sci.U.S.A* 1983;80(15):4842-4846.

124. Dover GJ, Humphries RK, Young N et al. Pharmacologic manipulation of fetal hemoglobin synthesis. *Prog.Clin.Biol.Res.* 1985;191:447-454.

125. Carr BI, Rahbar S, Asmeron Y, Riggs A, Winberg CD. Carcinogenicity and haemoglobin synthesis induction by cytidine analogues. *Br.J.Cancer* 1988;57(4):395-402.

126. Cheson BD, Bennett JM, Kantarjian H et al. Report of an international working group to standardize response criteria for myelodysplastic syndromes. *Blood* 2000;96(12):3671-3674.

127. Tomer A, Harker LA, Kasey S, Eckman JR. Thrombogenesis in sickle cell disease. *J.Lab Clin.Med.* 2001;398-407.

128. Francis RB, Jr. Elevated fibrin D-dimer fragment in sickle cell anemia: evidence for activation of coagulation during the steady state as well as in painful crisis. *Haemostasis* 1989;19(2):105-111.

129. Peters M, Plaat BE, ten Cate H et al. Enhanced thrombin generation in children with sickle cell disease. *Thromb.Haemost.* 1994;71(2):169-172.

130. Desimone J, Koshy M, Dorn L et al. Maintenance of elevated fetal hemoglobin levels by decitabine during dose interval treatment of sickle cell anemia. *Blood* 2002;99(11):3905-3908.

131. Wijermans PW, Krulder JW, Huijgens PC, Neve P. Continuous infusion of low-dose 5-Aza-2'-deoxycytidine in elderly patients with high-risk myelodysplastic syndrome. *Leukemia* 1997;11 Suppl 1:S19-S23.

132. Lubbert M, Daskalakis M, Kunzmann R et al. Nonclonal neutrophil responses after successful treatment of myelodysplasia with low-dose 5-aza-2'-deoxycytidine (decitabine). *Leuk.Res.* 2004;28(12):1267-1271.

APPENDIX A: BLOOD SAMPLES FOR GLOBAL METHYLATION, DNMT1, CDA genotype and CDA enZyme activity ANALYSIS (DR. YOGEN SAUNTHARARAJAH Laboratory).

Subject samples will be identified with their unique subject identifier, a sample number, and date of specimen collection. Subject names will not be used.

Quality control for DNA methylation and DNMT1 analysis will be maintained using the following mechanisms:

- Samples will be coded to preserve anonymity
- Buffy coat will be prepared and aspirated using standard methods from purple top (EDTA tubes)
- The remaining plasma will be aspirated and frozen at -80^o^C for later analysis of CDA enzyme activity
- Part of buffy coat will be used to prepare microscope slides for DNMT1 quantification by immuno-fluorescence
- DNA will be extracted from remaining buffy coat using standard methods, for CDA sequencing and bisulfate treatment and LINE1 methylation analysis by pyrosequencing..
- HPLC based methods modified from Richards et al in Biomed Chromatogr. 1987; 2(4):148-51 are used to measure CDA enzyme activity in the plasma (Xbridge™ OST C18, 2.5 µm, 4.6x50mm column on systems of waters 2695 separation module [Waters Assoc., Milford, MA] and Dionex UltiMate® 3000 [Sunnyvale, CA].

Requirement: One standard EDTA (purple top vacutainer) tubes, minimum 5 mL total blood volume in each tube. Samples at UIC will be sent to Don Lavelle or Kestis Vaitkus in the Joseph DeSimone laboratory for processing and batching. Samples at Cleveland Clinic will be sent to Reda Mahfouz in the Yogen Saunthararajah laboratory.

DNA for CDA sequencing and bisulphite methylation analysis, slides for DNMT1 immunofluorescence and plasma for CDA enzyme activity, will be batched and shipped to:

Yogen Saunthararajah, MD

Cleveland Clinic Taussig Cancer Institute

9500 Euclid Avenue, R40

Cleveland, OH 44195

Tel: 216 444 8170

Please email shipping notice to [saunthy@ccf.org](mailto:saunthy@ccf.org)

**APPENDIX B: BLOOD SAMPLES FOR PHARMACOKINETIC ANALYSIS**

Blood samples (approx 0.5ml each) will be drawn into tubes pre-loaded with heparin and THU 10µl of 10mg/ml solution (to prevent invitro degradation of decitabine) and kept in ice as soon as they are drawn. These samples are then centrifuged as soon as possible at 600 g for 5 min at 4 ^o^C. After separation, plasma will be transferred in 0.2 ml aliquots@ into pre-frozen vials and stored frozen at -80 ^o^C until analysis (or shipment). For shipment, send frozen samples in dry ice. The shipment should only be done from Monday to Wednesday by overnight courier. It may be risky to send samples on Thursday or after, as the package may be held up in a receiving area and get thawed out.

Shipment Address:

Jiang Wang, PhASR

460 W 12th Ave

BRT Room 414

Columbus, Ohio 43210

Appendix C: Blood Samples for mRNA Extraction

At selected sites, blood samples (two pink top tubes) will be collected for mRNA extraction. All samples for mRNA extraction will be analyzed in the lab of Dr. Roberto Machado at the University of Illinois at Chicago. Samples will be delivered to:

Dr. Roberto Machado

University of Illinois at Chicago

Institute for Personalized Respiratory Medicine

909 South Wolcott

COMRB Room 3168

Chicago, IL 60612

Appendix D: Clinical Studies with 5-azacytidine in Severe Sickle Cell Disease and β‑Thalassemia

5-azacytidine has been used in a number of clinical studies in subjects with sickle cell anemia or β-thalassemia ^62;122-124^. Sickle cell anemia subjects treated with 5-azacytidine at a dose of 2mg/kg/day for only 5 days showed marked increases in their HbF levels up to 22%, and did not experience substantial toxicities ^62^. F-cells, measured by the low sensitivity Kleihauer-Betke procedure increased up to 80%. In another study, 4 subjects with SCD were treated for 500, 200, 100, and 30 days with subcutaneous or oral 5-azacytidine. No marrow toxicity was observed on any of the regimens. For 3 subjects, the highest average F reticulocyte level was observed on a 3 consecutive day a week regimen. Oral 5-aza, given with tetrahydrouridine, produced a comparable F reticulocyte response. In the 2 subjects treated for more than 100 days, hemoglobin (Hb) levels increased from 8.5 to 12 g/dL and 8.0 to 9.2 g/dL, mean cell volume (MCV) and mean corpuscular hemoglobin (MCH) increased by 25%, and lysate HbF levels peaked at 12% and 20%. These early studies also demonstrated that a brief course (3 days per week) of 5-azacytidine caused increased γ-globin synthesis and was of therapeutic benefit to all but one sickle cell anemia subject treated, and even in this subject, modest HbF elevations were produced ^123;124^. These benefits included decreased irreversibly sickled cells, decreased dense cells, and decreased indirect bilirubin. Although there were insufficient subject data for inferential statistical analyses, the frequency of vaso-occlusive crises appeared to decrease in both subjects followed for more than 100 days ^39^. A common side effect seen in these studies was a dose-dependent decrease in absolute neutrophil count. A report by Lowery and Nienhuis (1993) summarized the results of 5-azacytidine treatment in β-thalassemia subjects with end stage disease for whom continued transfusion therapy was no longer beneficial. Two subjects treated for 30 months (1-2 mg/kg/day for 4 days/week once a month) showed improvement in their clinical status and QOL, with no long-term adverse affects. Hemoglobin increased 2-3 g/dL and was maintained without transfusion throughout the 30 months of the study. The mechanism of action and the overall experience using 5-azacytidine was summarized by Humphries et al, who concluded that the mechanism did not involve cytotoxicity; in 9 patients with SCD and thalassemia treated with 5-azacytidine, 8 of 9 had significant and one patient a modest increase in HbF ^124^.

Phase 1/2 Studies of Decitabine in SCD

Based on the earlier studies of 5-azacytidine in humans with SCD ^62^, the experience with decitabine in the primate model, and the literature showing that decitabine was not carcinogenic ^125^ and indeed had a tumor suppressor effect ^90;91^, a phase I/II trial was conducted of low dose IV administration of decitabine in HU refractory subjects.

*Phase 1/2 Study of Low Dose IV Administration of Decitabine in SCD*: The subjects treated in this initial study were 5 non-responders, 2 low responders, and 1 subject who was ineligible for HU treatment in the MSH study. These subjects were initially treated in a phase I/II, dose‑escalation study of decitabine ^70^. Decitabine was given intravenously at doses ranging from 0.15 mg/kg to 0.30 mg/kg for 5 days/week for 2 weeks. The average γ-globin synthesis relative to non-α-globin synthesis prior to therapy was 3.19% ± 1.43% and increased to 13.66% ± 4.35% after treatment. For all subjects, the average HbF increased from 3.55% ± 2.47% to 13.45% ± 3.69%. In the patients classified as HU non-responders, HU treatment increased HbF levels from a base-line of 2.28% ± 1.61% to 2.6% ± 2.15%. However, with decitabine treatment HbF levels increased to 12.7% ± 1.81% and F-cells increased from a baseline of 21%± 14.8% to 55% ± 13.5%. The HbF/F-cell ratio increased from 17% to 24%. Total Hb increased by 1 g/dL in 6 of 8 subjects. Platelet counts averaged a 2.2-fold increase at the 6^th^ week and returned toward baseline by week 7. This increase in platelets was mirrored by a transient 3‑fold decline in neutrophils, reaching a nadir at 6 weeks and returning toward baseline by week 7. Maximum HbF was attained within 4 weeks of treatment and remained at least 90% above maximum for 2 weeks.

Based on these encouraging results in HU non-responders, with no clinically significant toxicity, a second long-term trial of 36 weeks duration was conducted to identify the toxicity and effectiveness of repeated decitabine dosing over a 9-month period ^71^. All 7 subjects had been enrolled in the previous trial ^70^; 5 of the 7 were HU non-responders, and one was S-β‑thal. Decitabine was administered by IV infusion at 0.3 mg/kg/day, 5 consecutive days per week for 2 weeks. This treatment was followed by a 4-week observation period. If the absolute neutrophil count (ANC) dropped below 1000, the dose was reduced by 0.05 g/kg/day in the next 6-week cycle. An optimal drug dose was obtained for each subject, and resulted in an elevated HbF without neutropenia (ANC nadir >1500) or evidence of cumulative toxicity.

| Subject | Pre | HbF (%) | | Total Hemoglobin (g/dL) | | |
| --- | --- | --- | --- | --- | --- | --- |
|  |  | Avg | Max | Pre | Avg | Max |
| 1 | 0.8 | 12.40 ± 1.25 | 14.4 | 6.2 | 9.05 ± 0.48 | 9.6 |
| 2 | 6.8 | 14.55 ± 1.32 | 16.3 | 8.2 | 9.37 ± 0.60 | 10.3 |
| 3 | 1.4 | 12.75 ± 2.28 | 17.2 | 6.0 | 8.34 ± 0.55 | 9.5 |
| 4 | 0.6 | 10.80 ± 2.05 | 14.4 | 7.2 | 8.28 ± 0.52 | 9.0 |
| 5 | 2.9 | 16.42 ± 2.81 | 24.6 | 8.0 | 8.91 ± 0.57 | 9.6 |
| 7 | 6.2 | 16.70 ± 2.55 | 23.2 | 7.8 | 8.92 ± 0.79 | 10.4 |
| Mean ±SD | 3.12 ± 2.75 | 13.93 ± 2.35 | 18.35 ± 4.46 | 7.32 ± 0.94 | 8.81 ± 0.42 | 9.73 ± 0.53 |

**Table B1: Hemoglobin and HbF levels before and during the last 20 of 36 weeks of treatment with decitabine**

The average HbF and average maximal HbF levels attained during the last twenty weeks of treatment for the 7 SS subjects were 13.93 ± 2.35% and 18.35% ± 4.46%, respectively (from a base-line of 3.12 % ± 2.75%). The average and average maximal hemoglobin values were 8.81 ± 0.42 g/dL and 9.7 ± 0.53 g/dL, respectively (from a base-line of 7.23 ±2.35 g/dL)(**Table B1**). Individual maximal F-cell numbers during the trial ranged from 58 - 87% (ie, an average over all 7 subjects of 69 ± 10.12%). Despite periodic depressions in ANCs, which occurred 5 to 6 weeks after beginning each treatment cycle, the average ANC during the last 20 weeks of treatment (4200 ± 1350) was not significantly different from the pretreatment average (4600 ± 1560). The ANCs of 2 HU non-responder subjects never fell below 2000 and the nadirs, which occurred at 5-6 weeks of each cycle, generally remained above 3000. As in the previous study, decreases in ANC were probably caused by preferential differentiation into the megakaryocytic pathway at the expense of neutrophils: a peak in the platelet count that mirrored the ANC nadir was observed. Others ^126^ have also observed the increase in platelet count. No other toxicities were noted, and the subjects tolerated the treatment well.

Subjects treated with decitabine experienced only a 1-3 day period of neutropenia, and had a mirrored increase of their platelet counts with a small decrease of reticulocyte number. This is in contrast to those treated with HU, where hematologic toxicities are pancellular, with neutrophil recovery taking up to 2 weeks (AHFS Drug Information®, 2000). These observations suggested that decitabine may not be cytotoxic, but may induce changes in cellular differentiation ^71^.

*Phase I/II Study of Subcutaneous Administration of Decitabine in SCD:* To build on the above experience, we initiated a phase I/II study using decitabine given by the subcutaneous route. The objectives of this study were to assess the safety of decitabine given by the subcutaneous route, to produce cumulative increases in fetal and total hemoglobin through weekly administration and to explore the mechanism by which decitabine increases HbF ^72^. Between November 2001 to February 2002, eight subjects were enrolled into the study with informed consent. There were five females and three males, average age was 38 yrs with an age range between 22-61 years. All subjects had multiple clinically significant complications of SCD. Three subjects (Unit Patient number [UPN] 1, 3, 5) had been on HU for >1 year in the MSH and failed to demonstrate a >0.5g/dl increase in HbF or decreased symptoms. Compliance had been documented by HU blood levels and pill counts. The remaining subjects demonstrated increases in HbF and decreased frequency of their painful crises with HU. HU was discontinued for the following reasons: UPN2 and 4 developed lichen planus, which resolved upon discontinuation of HU; UPN6, 7 and 8 had leg ulcers that progressed on HU.

Toxicity. National Cancer Institute (NCI) Toxicity Criteria were used to assess toxicity. No local toxicity was documented at SQ injection sites. No subjects described nausea, vomiting, diarrhea, constipation, or decreased appetite. One patient had NCI grade 4 neutropenia (nadir ANC 0.4 x 10^3^/μl), two had grade 3 neutropenia (nadir ANC 0.8 x 10^3^/μl). All subjects recovered within a week. Neutropenic fever did not occur.

Increase in HbF. Ideally, any increase in HbF should be distributed in a pancellular fashion. Therefore the primary efficacy endpoint was > 80% F cells containing >20% HbF per cell. All subjects demonstrated statistically significant increases in %F-cells. HU non-responders (UPN 1, 3, 5) had lower %F-cells at baseline but demonstrated a rate of increase similar to that seen in HU responders (**figure B1**). The %F-cells increased by the second week after initiation and decreased by the second week after discontinuation of treatment. The primary endpoint of 80% F-cells was achieved by UPN8 during cycle 1 and UPN2, 4 and 7 during cycle 2 (**figure B1**).

The increase in the level of HbF as a percentage of total Hb (%HbF) mirrored the increase in %F-cells

***PatientsPre-tx F-cell% Max F-cell% p-value Pre-tx HbF%Max HbF%p-value*HU non-responders (n=3)**16.2 ± 10.8 55.1 ± 11.10.0022.4 ± 1.614.5 ± 2.20.0006**HU responders (n=5)**51.3 ± 6.381.2 ± 4.40.00019.0 ± 2.523.9 ± 3.1<0.0001**All Patients (n=8)**38.1 ± 7.671.4 ± 6.5<0.00016.5 ± 1.420.4 ± 2.0<0.0001Key: values are mean ± SD, p values are for 2-tailed paired t-test

**Figure B1: Decitabine increased HbF levels in all treated patients. A:** Change in HbF as a percentage of total hemoglobin with treatment. The HbF percentage was determined by alkali denaturation. **B:** Table shows changes in F-Cells and HbF in HU responders and non-responders. **C.** The peak HbF values with decitabine treatment were higher than those measured during previous treatment with HU for between 4-36 months. Peak HbF levels were not measured in UPN2 during HU treatment.

**A**

**C**

**B**

F/F Cell increased from 17% to 24%.

Peak %HbF levels during decitabine treatment were higher than peak levels measured during previous HU treatment (**figure B1C**). Peak levels were measured after 4-36 months treatment with HU.

Increase in Total Hemoglobin (tHB) and Decreased Hemolysis. Total Hb increased from 7.6 ± 2 to 9.6 ± 1.8 (mean ± 2SD of pre-treatment to peak Hb, paired t-test p <0.001)(**figure B2**).

Both the absolute reticulocyte count (ARC) (p=0.0006) and total bilirubin (p=0.01, 2-tailed paired t-test) decreased during treatment. The ARC correlated inversely with tHb (p<0.0001), suggesting that the ARC decrease resulted from decreased hemolysis (presumably due to selection of F cells, which have an increased life span resulting in increased hemoglobin).

**Figure B2: Increase in total hemoglobin during decitabine treatment**

Decreased RBC adhesion and correlation with hematological parameters. RBC adhesion to both TSP and laminin decreased following cycle 1 (p<0.005). In multivariate analysis, a significant association was noted between %F-cells and RBC adhesion to TSP and laminin (p=0.046 and p=0.004, respectively).

Changes in markers of coagulation activation, endothelial damage and inflammation and correlation with hematological parameters (Table B2). In SCD, abnormal exposure of molecules such as phoshphatidyl-serine on the RBC surface and adhesion of RBC to endothelial cells/endothelial damage can trigger coagulation and inflammatory pathways. In agreement with previous reports ^127-129^, increased levels in markers of active coagulation, Thrombin-antithrombin (TAT), F1+2 and D-dimers, were noted at baseline. Treatment decreased D-dimer levels, a measure of fibrinolysis of crosslinked fibrin (p<0.04), while markers of thrombin generation, TAT and F1+2, decreased but not to a statistically significant extent. The adhesion molecule soluble VCAM (sVCAM-1) and von Willebrand factor peptide (VWFpp) are released from damaged endothelial cells, levels of both molecules decreased with treatment (p<0.05). C-reactive protein (CRP), a marker of inflammation, was elevated at baseline and although there was a downward trend with therapy, it was not statistically significant (p=0.18).

For those markers that decreased significantly with treatment (D-dimers, sVCAM, VWFpp), we looked for correlations with hematological parameters (%F-cells, total hb, ARC and ANC). sVCAM levels inversely correlated with total hemoglobin (p=0.002), VWFpp levels correlated with the ARC (p<0.0001). There was no significant correlation between D-dimers and the hematological parameters. These correlations suggest the importance of increasing total Hb and decreasing the ARC (the most adhesive fraction of RBCs) in SCD.

Table B2. Changes in Indices of RBC adhesion, coagulation pathway activation, inflammation and endothelial damage during decitabine therapy. Values are mean ± SE; paired 2-tailed t-test. P* = significance of change from pre-therapy to post-cycle 1. P** = change from pre-therapy to post-cycle 2.

|  |  | Pretherapy | Post Cycle 1 | P* | Post Cycle 2 | P** | Normal Range |
| --- | --- | --- | --- | --- | --- | --- | --- |
| Measures of RBC adhesion to endothelium | Adhesion to TSP (RBCs/mm^2^) | 1570 ± 170 | 690 ± 150 | <0.001 | 910 ± 160 | <0.001 | < 60 |
|  | Adh. to  laminin  (RBCs/mm^2^) | 3470 ± 500 | 1950 ± 300 | 0.004 | 1570 ± 210 | <0.001 | < 250 |
| Measures of thrombin generation and fibrinolysis | D-Dimer (ng/mL) | 490 ± 90 | 320 ± 50 | 0.02 | 300 ± 50 | 0.03 | < 400 |
|  | TAT (ug/L) | 7.0 ± 1.7 | 8.6 ± 2.3 | 0.15 | 5.2 ± 0.9 | 0.11 | 1.0 - 4.1 |
|  | F1+2 (nmol/L) | 1.75 ± 0.22 | 1.56 ± 0.16 | 0.23 | 1.41 ± 0.15 | 0.051 | 0.04-1.1 |
| Measure of inflammation | CRP (mg/dL) | 1.25 ± 0.27 | 1.19 ± 0.34 | 0.80 | 0.82 ± 0.26 | 0.18 | < 0.7 |
| Measures of endothelial cell damage | sVCAM (ng/mL) | 1170 ± 140 | 930 ± 100 | 0.01 | 840 ± 100 | 0.02 | 395-714 |
|  | VWFpp (u/dL) | 196 ± 26 | 156 ± 28 | 0.015 | 144 ± 13 | 0.049 | 74-153 |

Increase in Platelet Counts. Platelet counts increased in all subjects during treatment. The highest platelet count was 877 000 x 10^9^/L. There was an inverse relationship between platelet and neutrophil counts (**figure B3**).

Change in Bone Marrow Morphology. Serial bone marrow aspirates were obtained in four subjects who consented to this procedure at enrollment. There was no appreciable decrease in aspirate spicule cellularity with treatment. Treatment decreased the myeloid: erythroid ratio (i.e., increased the proportion of erythroid cells) and increased megakaryocyte numbers in all subjects.

It is clear from the presented studies that decitabine is a very potent inducer of HbF. The induction of hypomethylation (**figure B3**) appears to be the major mechanism of action. Both local DNA hypomethylation at the *γ-globin* gene and altered hematopoietic differentiation are likely to contribute to the increase in F-cells.

**Figure B3:** **Decitabine at low-doses has a non-cytotoxic mechanism of action**. **A.** Platelet counts increase during periods of drug administration with rapid decreases off therapy (week 7-9). **B.** Neutrophil counts and platelet counts demonstrate an inverse relationship. **C.** Marrow cellularity did not decrease. **D.** CpGs in the γ-globin promoter became hypomethylated (green) during therapy.

**A**

**B**

**D**

**C**

A pilot study of subcutaneous decitabine therapy in patients with β-thalassemia intermedia

In five patients with β-thalassemia intermedia, a dose and schedule of decitabine intended to deplete DNMT1 without causing significant cytotoxicity (0.2 mg/kg subcutaneous 2x/week for 12 weeks) increased total hemoglobin (Hb) from a baseline of 7.88 ± 0.88 g/dL to a peak of 9.04 ± 0.77 g/dL (p = 0.004). Increases in Hb of ≥1.5 g/dL were achieved in two of the five patients. Absolute fetal Hb increased from a baseline of 3.64 ± 1.13 g/dL to a peak of 4.29 ± 1.13 g/dL (p = 0.003). Significant favorable changes were also noted in indices of red blood cell densitometry and hemolysis (**Table B3 and B4**).

**Table B3: Subcutaneous decitabine treatment significantly improved total hemoglobin, HbF levels, and RBC volume distribution, and significantly decreased bilirubin levels, in 5 β-thalassemia intermedia patients. Favorable trends were also noted in the other parameters measured. *=p<0.05, **p<0.01**

| Pt # | Hemoglobin  (g/dL) | | | HbF  (g/dL) | | | Indirect Bilirubin  (µmol/L) | | | Serum LDH  (U/L) | | | EPO  (mIU/ml) | | |
| --- | --- | --- | --- | --- | --- | --- | --- | --- | --- | --- | --- | --- | --- | --- | --- |
|  | B/L | Peak** | Wk 12* | B/L | Peak** | Wk 12* | B/L | Nadir* | Wk 12 | B/L | Nadir | Wk 12 | B/L | Nadir | Wk 12 |
| 1 | 8.8 | 9.4 | 9.4 | 7 | 7.5 | 7.1 | 25.7 | 27.4 | 30.8 | 893 | 660 | 660 | 197.8 | 117.2 | 117.2 |
| 2 | 10.7 | 11.8 | 11.8 | 0.1 | 0.7 | 0.7 | 104.3 | 83.8 | 83.8 | 596 | 541 | 556 | 25 | 17.1 | 24.4 |
| 3+ | 8 | - | - | 1.7 | - | - | 47.9 | - | - | 543 | - | - | 54.8 | - | - |
| 4 | 6.1 | 7.7 | 6.4 | 2.8 | 3.5 | 3.4 | 89 | 48 | 48 | 458 | 260 | 324 | 241.4 | 131.4 | 238.5 |
| 5 | 7.8 | 8.7 | 8.7 | 4.8 | 5.6 | 5.6 | 33 | 18 | 33 | 192 | 149 | 167 | 186 | 180.7 | 340.5 |
| 6 | 6 | 7.6 | 7.5 | 3.6 | 4.1 | 3.6 | 20 | 8 | 10 | 258 | 204 | 204 | 86.6 | 71.5 | 71.5 |

| Pt # | Deformability Index (AU) | | | Normochromic Normovolumetric Cells (%) | | | Annexin-V RBC  (%) | | |
| --- | --- | --- | --- | --- | --- | --- | --- | --- | --- |
|  | B/L | Peak | Wk 12 | B/L | Peak* | Wk 12* | B/L | Nadir | Wk 12 |
| 1 | 0.38 | 0.4 | 0.39 | 36 | 45.9 | 45.9 | 0 | 0 | 0.5 |
| 2 | 0.47 | 0.52 | 0.42 | 67.4 | 70 | 69 | 2 | 0 | 0.7 |
| 3+ | 0.58 | - | - | 45.5 | - | - | 0.8 | - | - |
| 4 | 0.47 | 0.53 | 0.5 | 65.4 | 70.9 | 70.5 | 0.6 | 0.1 | 0.1 |
| 5 | 0.34 | 0.63 | 0.58 | 63.9 | 70 | 66.4 | 3.4 | 0.4 | 2.6 |
| 6 | 0.51 | 0.56 | 0.46 | 61.2 | 72.4 | 68.1 | 0.8 | 0 | 1.1 |

Table B4: Changes in the other laboratory parameters in the treated β-thalassemia patients. *=p<0.05, **p<0.01

| Pt # | Absolute Retic count  (x 109/L) | | | Neutrophil count  (x 109/L) | | | Platelet count  (x 109/L) | | |
| --- | --- | --- | --- | --- | --- | --- | --- | --- | --- |
|  | B/L | Nadir* | Wk 12* | B/L | Nadir | Wk 12 | B/L | Peak** | Wk 12* |
| 1 | 165 | 135 | 135 | 5.57 | 2.82 | 4.62 | 233 | 296 | 281 |
| 2 | 155 | 160 | 160 | 5 | 2.2 | 2.2 | 625 | 1038 | 1038 |
| 3+ | 165 | - | - | 5.4 | - | - | 536 | - | - |
| 4 | 163 | 116 | 116 | 5.6 | 5.2 | 7.9 | 720 | 1265 | 1265 |
| 5 | 196 | 125 | 145 | 5.1 | 2.1 | 10.3 | 627 | 801 | 756 |
| 6 | 106 | 79 | 79 | 11.3 | 4.5 | 5.7 | 721 | 1301 | 1301 |


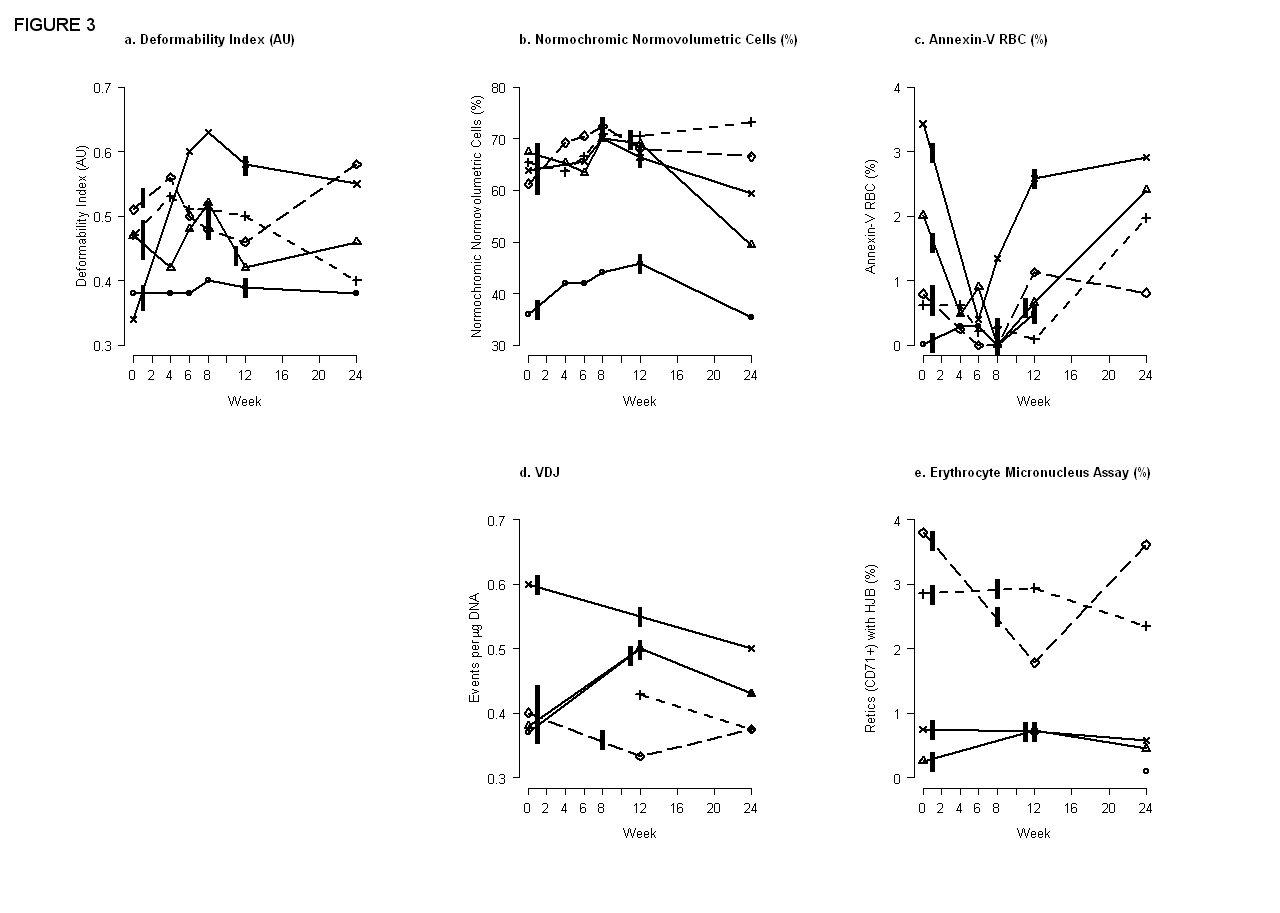


**Figure B4: Changes in RBC deformability, hb content & volume, phosphatidyl-serine exposure, VDJ locus recombination and erythrocyte micronucleus assay during and after treatment**. The vertical black lines indicate the time of first and last decitabine injections in each patient. Some patients were treated for <12 weeks because platelet counts increased above apriori demarcated levels.

Consistent with a non-cytotoxic, differentiation altering mechanism of action, the major side-effect was an asymptomatic increase in platelet counts (**Table B4**) without erythrocyte micronucleus or VDJ recombination assay evidence of genotoxicity (**Figure B4**). This first and multi-center pilot experience with decitabine in β-thalassemia provides reiterated the clinical safety and efficacy advantages that may be possible with direct epigenetic therapy with decitabine.

Chronic Administration of Decitabine in Sickle Cell and Other Diseases

There has been human experience with chronic administration of decitabine, in SCD and malignant hematologic disorders, in some cases for periods of time and with cumulative doses that exceed those proposed for this study

Chronic decitabine administration in SCD ^16^: Previous studies of decitabine as a potential disease-modifying agent for sickle cell disease (SCD) involved relatively short-term drug administration, limiting ability to demonstrate clinical effectiveness. In four SCD patients with severe acute illness on a background of chronic clinical deterioration over the preceding years or months, decitabine (0.1-0.2mg/kg 1-2X/week) was administered for periods beyond 12 months. Hemoglobin increases of >1.5g/dl occurred within 2-4 weeks with maximum hemoglobin increases of 3.5-5g/dl. Hemoglobin increased through a direct, differentiation-mediated increase in reticulocytes and an increase in fetal hemoglobin. Durable symptom and performance status improvement during 4-12 months of follow-up contrasted with severe and deteriorating pre-decitabine trends.

Generally, reticulocyte counts increased during the first 2-8 weeks of therapy. Reticulocyte trends reversed after hemoglobin levels >9g/dl, presumably because of negative feed-back on endogenous erythropoietin production. This was most obvious in Patient A who was not receiving exogenous erythropoietin. The clinical status of the patients for 12 months before decitabine and during decitabine therapy is summarized in **table B5**.

Although this was an off-label, non-protocol experience, it is suggestive of clinical effectiveness for the following reasons: all patients had severe acute illness on a background of chronic deterioration and progressive anemia over the preceding years or months; the follow-up period ranging from 4-12 months allowed documentation of durable clinical improvement that contrasted convincingly with clinical status and trends in the preceding months; although 3 of the 4 patients were on concurrent erythropoietin, it had been administered at stable doses for more than 6 months with progressive anemia and recurrent severe anemia exarcebations; although 2 of the 4 patients received transfusions during decitabine therapy, these do not explain the durable increases in hemoglobin and eventual transfusion independence.

Decitabine is approved by the US FDA for the treatment of myelodysplastic syndrome. The above off-label use of decitabine in SCD was to provide direct benefit to these patients and not for research. Decitabine was considered because of clinical deterioration and life-threatening complications despite HU therapy, erythropoietin for relative reticulocytopenia (hemoglobin <9g/dl & reticulocytes ≤250x10^9^/L), decreased availability and increased transfusion risks from ≥ 5 red blood cell (RBC) allo-antibodies and autoantibodies, and ineligibility for available protocol therapy. The severe and complicated clinical circumstance in these patients is not typically represented in clinical trials. Therefore, this description can complement the clinical studies and provide additional guidance regarding dose, schedule, anticipated toxicities and inclusion criteria.

A previous trial examined whether decitabine could be administered repeatedly to subjects with SCD and maintain elevated HbF levels for an extended period of time (36 weeks = 252 days) without toxicity ^130^. Seven subjects were treated and the maximum cumulative dose administered to a subject was 1080 mg. No cumulative hematologic or non-hematologic toxicities were indicated (**Table B1**).

*Chronic decitabine administration in myelodysplastic syndrome*: Chronic administration of decitabine has been studied in two large-scale clinical trials in subjects with myelodysplastic syndrome (MDS). Initially, decitabine was chronically administered to 124 subjects with MDS. The doses used were between 40‑50 mg/m^2^ 3X/wk with courses repeated every 6-8 weeks for up to seven courses (for an individual with a body surface area (BSA) of 1.8, the cumulative dose at 45 mg/m^2^ would be 1701 mg over a period of 294 days). The agent was well tolerated with major cytogenetic responses noted in 31% of those subjects with abnormal cytogenetics at diagnosis ^82^. Fourteen subjects with high-risk MDS were treated for up to 1310 days with a maximum cumulative dose of 3369 mg ^131^. In a follow-up study, the investigators re-treated 22 of these subjects upon relapse of their disease with a median of three additional courses of therapy. Again, therapy was well tolerated in these subjects with a median age of 71 years ^132^. The investigators in both of these studies have concluded that decitabine is a drug with rather mild toxicity that was well tolerated even in older subjects with high-risk MDS.

In the second study, 80 of 160 subjects were randomized to receive decitabine. Subjects received decitabine at 45 mg/m^2^/day for 3 days approximately every 6 weeks with up to 10 cycles of decitabine administration. For an individual with a BSA of 1.8 (weight 70 kg, height 170 cm), the maximum amount of decitabine received was 2430 mg over a period of 420 days. In these mostly elderly subjects with a hematological malignancy, the investigators concluded that decitabine was tolerated well by most subjects; there were no treatment related deaths (Saba et al, 2004).

**Table B5: Clinical Status of Patients before and during decitabine therapy**

|  | **Significant Clinical Events and Overall Clinical Status** | |
| --- | --- | --- |
|  | **In 12m before decitabine administration** | **During decitabine administration** |
| **Patient A:** 22y, female, HbSS | >16 ER visits, >290 hospital in-patient days | (12m follow-up)  7 ER visits, <30 hospital in-patient days |
|  | Life-threatening hyperhemolysis  Life-threatening hepatic crisis, severe pain on long-acting opiates and regular IV opiates | No life-threatening crises, mostly out-patient pain-management without need for long-acting opiates |
|  | Transfusion x7 | No transfusions |
|  | ECOG PS 3* | ECOG PS 1-2 |
| **Patient B:** 36y, female, HbSS | >11 ER visits, >150 hospital in-patient days in 180 day period prior to decitabine initiation | (8m follow-up)  3 ER visits, >60d at home without ER visits, longest period without admission in preceding 20 months |
|  | CHF, severe pain requiring IV narcotics | No CHF, out-patient pain management with oral meds |
|  | Transfusion x4 | No transfusions |
|  | ECOG PS 3* | ECOG PS 1-2 |
| **Patient C:**63y, female, HbSS | 89 day hospital admission for intractable and life-threatening CHF | (7m follow-up)  Resolution of CHF and discharge to home |
|  | Transfusion x4 | Transfusion x4 (triggered by attempts to discontinue decitabine or darbopoietin and a GI bleed)(no transfusion required in latest 8 week period) |
|  | ECOG PS 4* | ECOG PS 2 |
| **Patient D:** 22y, female, HbSS | >10 ER visits, 111 hospital in-patient days | (4m follow-up)  >60d at home without ER visits, longest period without admission in preceding 16 months |
|  | Life-threatening CHF and severe pain | Stabilization and improvement in CHF and pain allowing discharge to home |
|  | Transfusion x5 | Transfusion x6 (no transfusion required in latest 8 week period) |
|  | ECOG PS 3-4* | ECOG PS 2 |
